# Resorbable conductive materials for optimally interfacing medical devices with the living

**DOI:** 10.3389/fbioe.2024.1294238

**Published:** 2024-02-21

**Authors:** Marta Sacchi, Fabien Sauter-Starace, Pascal Mailley, Isabelle Texier

**Affiliations:** ^1^ Université Grenoble Alpes, CEA, LETI-DTIS (Département des Technologies pour l’Innovation en Santé), Grenoble, France; ^2^ Université Paris-Saclay, CEA, JACOB-SEPIA, Fontenay-aux-Roses, France

**Keywords:** conductive, resorbable, biopolymer, conducting polymers, bioelectronics, implanted sensors, wearable sensors, tissue engineering

## Abstract

Implantable and wearable bioelectronic systems are arising growing interest in the medical field. Linking the microelectronic (electronic conductivity) and biological (ionic conductivity) worlds, the biocompatible conductive materials at the electrode/tissue interface are key components in these systems. We herein focus more particularly on resorbable bioelectronic systems, which can safely degrade in the biological environment once they have completed their purpose, namely, stimulating or sensing biological activity in the tissues. Resorbable conductive materials are also explored in the fields of tissue engineering and 3D cell culture. After a short description of polymer-based substrates and scaffolds, and resorbable electrical conductors, we review how they can be combined to design resorbable conductive materials. Although these materials are still emerging, various medical and biomedical applications are already taking shape that can profoundly modify post-operative and wound healing follow-up. Future challenges and perspectives in the field are proposed.

## 1 Introduction

**FIGURE 1:**
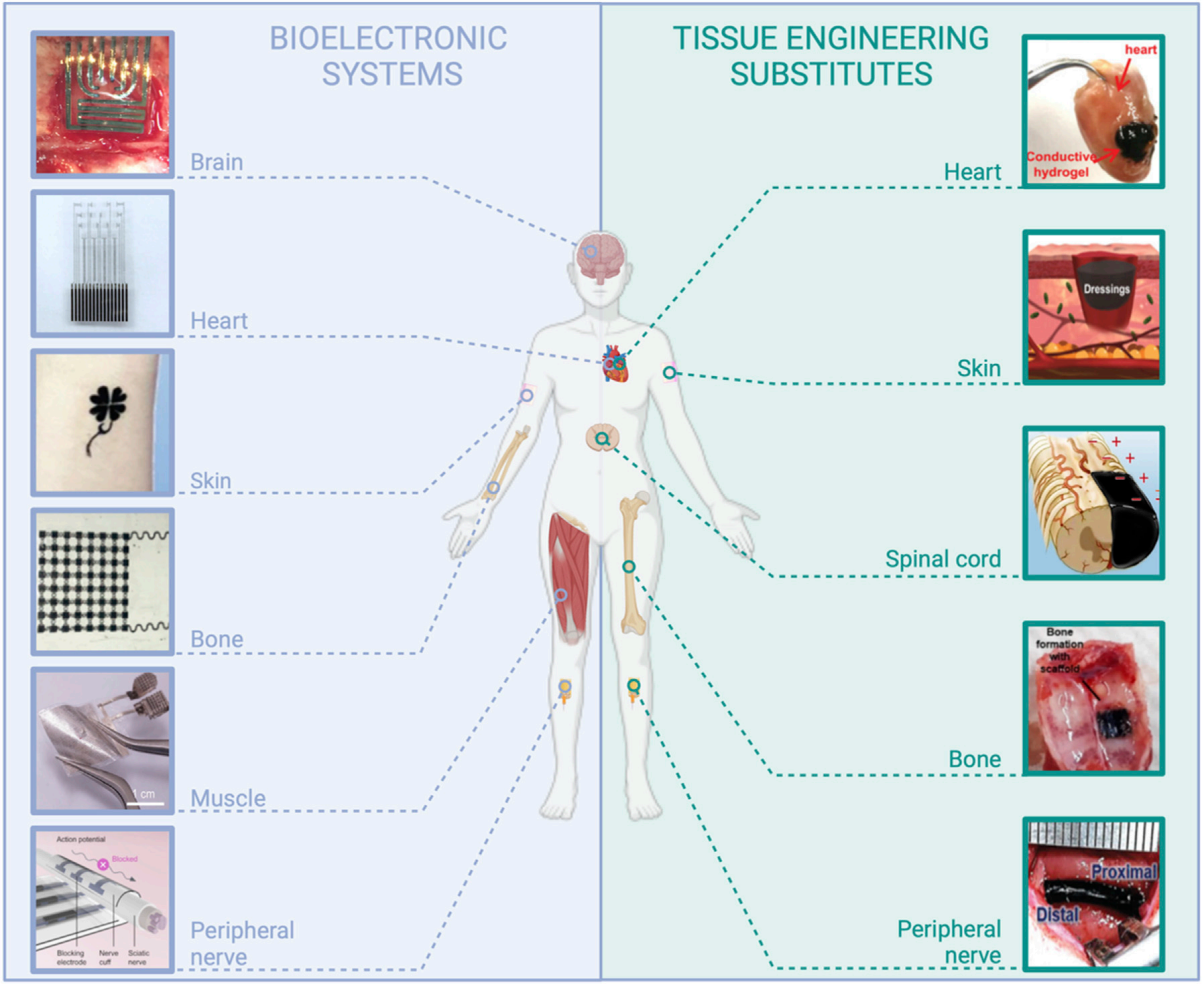
Examples of applications in the field of medical devices requiring the use of resorbable conductive materials. *Bioelectronic systems*: brain: reproduced with permission, Copyright 2019, John Wiley and Sons (Xu K. et al., 2019). Heart: adapted with permission, Copyright 2023, the Authors, published by *Science Advances* (Chen et al., 2023). Skin: reproduced with permission, Copyright 2019, John Wiley and Sons (Wang Q. et al., 2019). Bone: adapted with permission, Copyright 2021, the Authors, published by *PNAS* (Yao et al., 2021). Muscle: adapted with permission from Huang et al. (2022), Copyright 2022, the American Chemical Society. Peripheral nerve: Copyright 2022, the Authors, published by *Science Advances* (Lee et al., 2022). *Tissue engineering substitutes*: Heart: adapted from Xu Y. et al. (2019), Copyright 2019, John Wiley and Sons. Skin: adapted with permission from Huang et al. (2018), Copyright 2018, the American Chemical Society. Spinal cord: adapted from Chen et al. (2022), Copyright 2022, Springer Nature. Bone: reproduced from eSilva et al. (2021), Copyright 2021, Springer Nature. Peripheral nerve: reproduced with permission, Copyright 2020, John Wiley and Sons (Park et al., 2020), created with BioRender.com.

The current landscape of electronic systems in the medical field is diverse, encompassing wearable and implantable devices tailored to various applications, such as drug delivery, occasional or continuous monitoring, or stimulation. These systems primarily rely on traditional materials like metals, semiconductors, and plastics and conventional processes such as patterning and lithography from the realm of microelectronics. In recent years, much effort has been dedicated to conferring these systems with mechanical properties more suitable for *in vivo* use, for instance, with the employment of thinned structures, and the development of processes compatible with flexible and stretchable substrates such as elastomers (Palma et al., 2022; Veletić et al., 2022). However, the large majority of presently used medical bioelectronic systems display a fundamental difference with living tissues: they are not resorbable, meaning they are not composed of materials that progressively dissolve in the body or onto the skin, without inducing toxicity or immunogenicity. Long-term implants are required for a set of medical applications that are presently addressed (e.g., cardiac and neural implants such as deep brain stimulation implants for Parkinson’s disease or vagus nerve stimulation devices), or for which it would be highly desirable to prolong the device lifetime (e.g., continuous glucose monitoring sensors, drug delivery pumps, etc.). However, innovative applications could emerge with the advent of resorbable, wearable, or implanted medical bioelectronic systems (Figure 1). Resorbable bioelectronics can be used for the design of microelectrode arrays for transient neuromodulation (brain, spinal cord, and peripheral nerve), on-skin sensors, and heart, skin, muscle, or bone stimulation to promote healing. More prospectively, resorbable sensors can be dedicated to post-surgical follow-up to alert on infection risks or ensure the success of a graft (tissue anastomosis). Resorbable conductive materials are also intensively sought for tissue engineering, in particular in the case of electro-responsive organs such as the heart, nerves, or skin, and for demanding *in vitro* applications, particularly in the field of 3D cell culture models (Guo et al., 2018; Park et al., 2022b; Tringides et al., 2022).

It is noteworthy that material resorbability in the body or onto the skin is more demanding than biodegradability, which may also encompass degradation in the natural environment under a more general definition. In the body, biodegradable materials degrade in smaller fragments that can eventually diffuse from their implantation site but not necessarily be eliminated. Contrarily, resorbable materials are totally eliminated from the body. These are materials that degrade into safe, smaller components when exposed to physiologically relevant conditions like biological fluids and enzymes. These resulting components and by-products are subsequently removed from the body via either metabolic processes or excretion (Eglin et al., 2008; Grosjean et al., 2023). Material resorbability can occur through different mechanisms, i.e., chemical and enzymatic degradations. The main chemical degradation processes occur through the hydrolysis of the polymer backbone bonds. Notably, the kinetics of degradation of commonly used synthetic polyesters such as poly(lactic acid) (PLA), poly(lactic-co-glycolic acid) (PLGA), and poly(hydroxyalkanoates) (PHA) can be controlled by their molecular composition, polymer molecular weight, and crystallinity (Bano et al., 2018). Natural polymers such as collagen, gelatin, and hyaluronic acid are primarily degraded by proteolytic and glycolytic enzymes.

**FIGURE 2:**
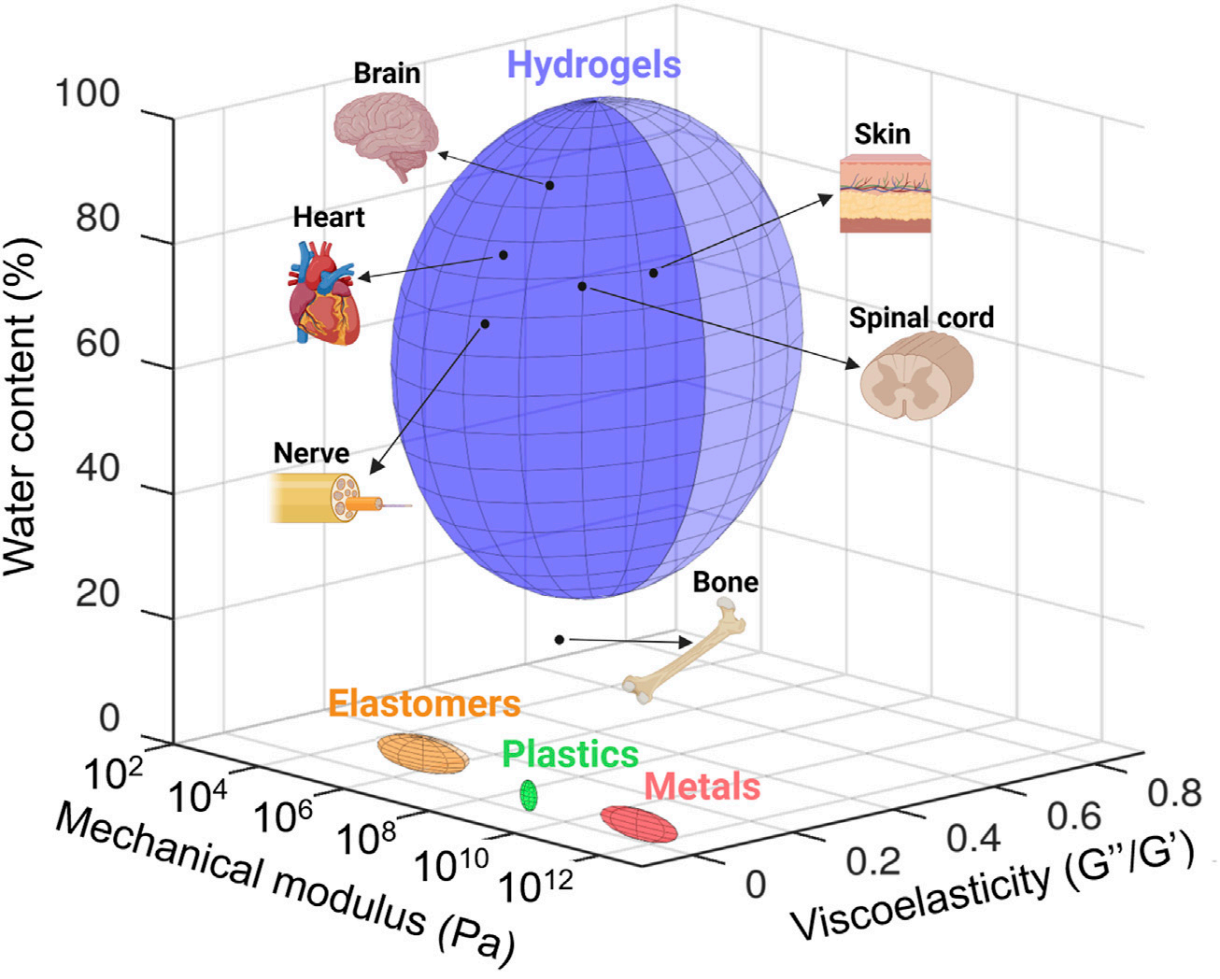
Quantification and comparison of the mechanical modulus (Pascal, Pa), viscoelasticity (loss modulus/storage modulus, G″/G′), and water content (percentage of weight) of tissues with those of materials classically employed in bioelectronic devices and tissue engineering (i.e., metals, plastic, elastomers, and hydrogels). Data taken from literature. Mechanical modulus: brain (Hall et al., 2021), heart (Jacot et al., 2010), spinal cord (Karimi et al., 2017), nerve (Rosso et al., 2019), skin (Kalra et al., 2016), bone (Morgan et al., 2018), and materials classically employed in bioelectronic devices and tissue engineering (Tringides et al., 2022). Viscoelasticity: adapted from Tringides et al. (2022). Water content: bone (Surowiec et al., 2022), nerve (Anand et al., 1988), spinal cord (Mbori et al., 2016), skin (Téllez-Soto et al., 2021), heart (Eitel et al., 2011), and brain (Gottschalk et al., 2021). Created with Octave (software version 8.2.0) and adapted on BioRender.com.

In addition to biocompatibility, materials employed in designing medical devices must fulfill additional requirements due to their intimate interaction with tissues. Indeed, these tissues can display a wide range of mechanical properties. First, bones, tendons, and nerves can be considered very hard and poorly stretchable tissues, with mechanical stiffness quantified by Young’s modulus in the decreasing order of 12 GPa (Keller et al., 1990), 550 MPa (O’Brien et al., 2010), and 580 kPa (Borschel et al., 2003), respectively. They exhibit high elastic moduli, similar to those of plastic materials (Figure 2). The skin is both relatively tough and stretchable (0.5–1 MPa elastic modulus) (Li et al., 2012), whereas very soft tissues like the brain display very high viscoelastic properties (elastic modulus G′ below 10 kPa, elastic-to-viscous moduli ratio G″/G′ > 0.5) (Hall et al., 2021; Tringides et al., 2022). In any case, tissue mechanical properties are quite different from those of rigid metal, plastics, or elastomers, classically employed in electronic devices (Figure 2) (Sunwoo et al., 2021). Such a mechanical mismatch between these materials and tissues at the intimate cellular level can be responsible for undesired effects, ranging from progressive material encapsulation by tissues, therefore inhibiting the correct functioning of the device, to acute inflammatory reaction and pain (Rivnay et al., 2017; Sunwoo et al., 2020). Another remarkable difference between tissues and materials such as metals, semiconductors, and elastomers presently used in bioelectronics consists in their morphological structure. While tissues display a macroporous/microporous structure, most of these synthetic materials are shaped as solid bulk or thin continuous layers without either no or poor porosity. Very importantly, many tissues are intimately exposed to biological fluids like interstitial fluid and blood, even sometimes with shear flow as in the cardiac and circulatory systems, and many of them, in particular soft tissues, are continuously bathed in these fluids making them an integral part of the tissue itself (Figure 2). By comparison, metals, semiconductors, and synthetic elastomers do not comprise water. For tissue engineering applications, material water content is an important hallmark to allow the necessary perfusion of nutrients, oxygen, and biological cues. In these perspectives, hydrogels—3D cross-linked polymer networks that can absorb large quantities of water—are interesting to explore. Their mechanical properties can be tuned across a wide range of Young’s modulus and viscoelasticity to design scaffolds with mechanical properties similar to those of the different types of tissues, from bone to brain (Figure 2) (Yuk et al., 2022). However, they are not intrinsically electronic conductors (though they can be ionic conductors), and consequently, they cannot be used as such to design electrically conductive materials for medical applications that require moderate to high electronic conductivity (10^3^–10^5^ S cm^−1^). Therefore, for all the above reasons, it is clear that beyond resorbability, bioelectronics and tissue engineering applications can advantageously benefit from the use of innovative conductive materials that better display the fundamental characteristics of tissues, i.e., hydrogel-based materials.

Passive (i.e., non-conductive) resorbable polymer-based medical devices have been extensively developed for the short-term or prolonged delivery of active ingredients. These drug-delivery implants are mainly based on synthetic polyesters like PLA, PLGA, poly(caprolactone) (PCL), and PHA that are sometimes also referred to as “bioplastics” (Bano et al., 2018) or on resorbable biopolymer-based hydrogels. Though bioplastics and biopolymer-based hydrogels are both resorbable, these materials differ markedly by their mechanical and swelling properties (“tough” and quite hydrophobic materials for bioplastics and “soft” and wet materials for hydrogels). Resorbable synthetic polyesters, such as PLA, PLGA, and PHA, are interesting because they can act as insulating supports or passivation layers in bioelectronic systems, taking advantage of their rather hydrophobic properties. First resorbable elastomers have also been recently reported (Turner et al., 2022). Hydrogels, from their side, are by definition highly hydrophilic materials and not suitable to act as insulating substrates in the design of bioelectronic systems, but they can be combined with conductive moieties to obtain suitable interfaces (for instance, electrodes) with the tissues or for the development of 3D cell cultures or organoids (Caliari et al., 2016; Kozlowski et al., 2021). Silk fibroin is a resorbable natural polymer combining the interesting features of both bioplastics (i.e., mechanical properties) and hydrogels (i.e., porous biopolymer scaffold).

Concerning electrical conductors, the interest in conducting polymers such as poly(pyrrole) (PPy), poly(aniline) (PANI), and poly(3,4-ethylenedioxythiophene) (PEDOT) to optimize system/tissue bioelectronic interface was underlined several times due to both their ionic and electronic conductivity (Chen et al., 2021; Han et al., 2022). Indeed, Sansinena et al. (1997) and Kim et al. (2000) first pointed out the interest in such conductive materials, used in conjunction with hydrogels, for the design of artificial muscles exhibiting interesting actuation capabilities. However, other conductive materials of high interest are largely employed in bioelectronics and tissue engineering applications, especially when it is necessary to achieve high material conductivities. There exist a few biocompatible and resorbable metals such as molybdenum, tungsten, and iron. Two-dimensional (2D) transition-metal chalcogenides (e.g., MoS_2_ sheets) and carbon-based structures and fillers like carbon nanotubes (CNTs) and graphene derivatives are also commonly employed materials.

In this review, we will describe how resorbable substrates and scaffolds such as elastomers, synthetic polyesters, and biopolymer-based hydrogels can be combined with a variety of electrical conductors like metals, micro- and macro-structured fillers, and conducting polymers, to lead to resorbable conductive materials with a wide range of mechanical properties. Some reviews have already discussed related subjects, such as conductive hydrogels (Rogers et al., 2020; Xu et al., 2020; Chen et al., 2021; Xu et al., 2021; Gao et al., 2022; Zhu et al., 2023a), conducive materials for neural interfaces (Fattahi et al., 2014), and conductive materials for tissue engineering (Min et al., 2018; Mostafavi et al., 2020; Rogers et al., 2020; Zhao et al., 2022b; Gao et al., 2022). However, we herein focus specifically on conductive materials that exhibit resorbability, a topic that has received comparatively less attention, especially for bioelectronic systems. As discussed earlier, the resorbability of conductive materials holds significant relevance for both the fields of bioelectronic devices and tissue engineering. It is worth noting that there is a noticeable gap in the existing literature concerning a comprehensive work that effectively merges the domains of soft bioelectronic systems that interact with living tissues and tissue engineering. This review aims to bridge the gap between these two distinct yet interconnected fields, which both rely on similar polymer and electrical components, as well as process methodologies, especially when it comes to resorbable conductive materials. Thus, we aim to provide readers with a review and thorough analysis of various approaches within the context of bioresorbable materials, allowing for an in-depth exploration of specific challenges and opportunities, encompassing both bioelectronics and tissue engineering applications. Special emphasis will also be placed on resorbable hydrogel-based materials. As highlighted earlier, these materials exhibit exceptional properties for replicating the characteristics of living tissues, making them a natural choice for tissue engineering applications. Furthermore, our extensive literature review has unveiled that the utilization of resorbable hydrogels represents a smart and emerging approach to the design of resorbable bioelectronic devices. While this review encompasses resorbable conductive materials and their applications in a broader sense, we will particularly emphasize the role of hydrogel-based systems in this context. Note that although these are closely related domains, we will not review here electronic textiles and refer the reader to other publications in that field (Kim et al., 2019; Alhashmi Alamer et al., 2022; Gong et al., 2022; Zhang et al., 2022; Wei et al., 2023).

Following the examination of various resorbable substrates and scaffolds, we will review the selection of electrical conductors that present resorbability. Subsequently, we will describe the different processes that can be used to combine them into resorbable conductive materials. These processes can impact the resorbability, conductivity, and mechanical properties of the resultant resorbable devices. These innovative resorbable conductive materials have broad applications, such as the development of novel transient bioelectronic systems for monitoring and stimulation, which will not require retrieval surgery after they have served their purpose. Additionally, they hold promise for on-skin electronic applications, 3D cell culture, and tissue engineering, particularly in the context of electro-sensitive organs like the heart, nerves, brain, and skin. They are therefore expected to address emerging applications in the biomedical field.

## 2 Selecting components for the design of resorbable conductive hydrogels

Resorbable conductive materials rely on the combination of a 2D substrate or a 3D scaffold and an electrical conductor. In this section, we will give a short overview of these materials.

### 2.1 Substrates and scaffolds

**FIGURE 3:**
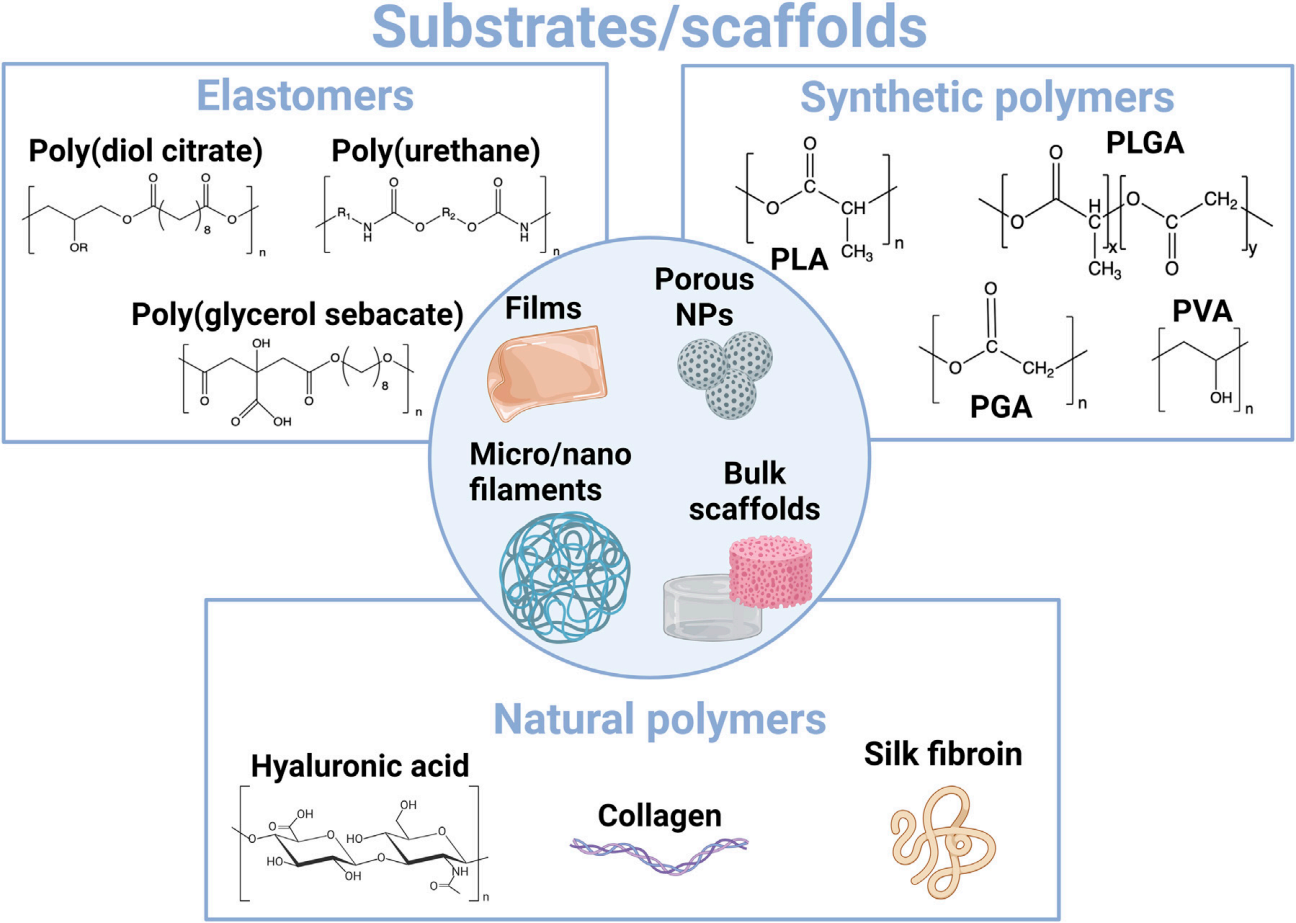
Overview of main materials employed for the construction of resorbable substrates and scaffolds at the interface with tissues.

The different resorbable materials that can be employed as 2D substrates or 3D scaffolds (Figure 3) do not display similar mechanical properties (Young’s modulus and viscoelasticity), the same hydrophobic/hydrophilic properties, or degradation kinetics and mechanisms (Figure 2) (Tringides et al., 2022). Since it is desirable to select a material whose mechanical properties match as much as possible those of the tissue that it is in contact with to limit discomfort and inflammation, the different types of substrates and scaffolds are used for different applications. Classical substrates for the design of wearable or implantable bioelectronic systems are mainly based on elastomers, such as silicone rubber or poly(urethane)-based films. These materials exhibit high chemical stability and can withstand very large strain rates. Therefore, they are particularly well-suited for seamlessly integrating with tissue movements, such as stretching, bending, and torsion, in particular for their application in skin electronics. They are also good candidates for applications involving tubing or highly flexible structures, such as cuff electrodes. However, the design of resorbable elastomers is still an emerging field (Turner et al., 2022). Therefore, bioplastics such as resorbable polyesters have driven much attention, especially considering that some of them, such as PLGA, PLA, and PHAs, are already approved implant materials by regulatory agencies (the Food and Drug Administration and European Medical Agency) (Nair et al., 2007; Ulery et al., 2011; Bano, 2018; Lu et al., 2023a). Elastomers and bioplastics are mainly employed as 2D thin-film substrates that are assembled with electrical conductors to design multilayer electronic systems. When aiming to create more viscoelastic 3D scaffolds, for instance, for 3D cell culture or tissue engineering applications, biopolymer-based hydrogels are more appropriate. From its natural origin, silk can display both hydrogel resorbability and eventual swellability and bioplastic-like mechanical properties. It is therefore a particularly relevant polymer to use as a resorbable substrate and packaging material in bioelectronics.

#### 2.1.1 Resorbable elastomers

Recent advancements in the design, synthesis, and medical uses of resorbable elastomers, mainly polyurethanes (PUs), poly(glycerol sebacate), and poly(diol citrate) (Figure 3), have been recently reviewed (Turner et al., 2022). Polyurethanes are a large class of copolymers where at least two organic monomers react to create a carbamate bond. Typically, PUs are produced by the reaction between a diisocyanate and polyol. A judicious choice of reactants makes it possible to confer degradability to the polymer, for instance, by selecting degradable monomers comprising an ester bond (Christenson et al., 2007). PUs are interesting in medical applications for their biocompatibility, the versatility of their chemical structure, and their high stretchability. The addition of poly(urethane) cross-links in an already resorbable bioplastic material can also confer it with elastomeric properties while maintaining its resorbability (Sharma et al., 2018). For instance, Sharma et al. designed a resorbable elastomeric stent based on a network of poly(glycolide-co-caprolactone) chains cross-linked with short polyurethane segments. The material, initially resorbable and stretchable, but without any deformation reversibility, displayed elastomeric behavior after PU cross-linking.

Poly(glycerol sebacate) (PGS) elastomers are polyesters obtained by the polycondensation of FDA-approved glycerol and sebacic acid. Due to their high biocompatibility, elastomeric properties, and biodegradability, PGS elastomers constitute materials of choice for medical applications (Rai et al., 2012). They have been used mainly in tissue engineering, in particular for neural and cardiac tissues, and wound healing. More recently, PGS elastomers have been combined with different conductive materials for the design of smart textiles that include pressure, strain, and temperature sensors (Vogt et al., 2021). Poly(diol citrate) elastomers are also polyesters derived from the condensation of citric acid with polyols during thermal treatment, with potential for tissue engineering applications. Poly(octamethylene maleate (anhydride)) (POMaC) has been combined with PGS to package resorbable strain and pressure sensors made of PLA and magnesium (Boutry et al., 2018). The resorbable device was intended to follow up tendon repair and self-degrade after its service to avoid second retrieval surgery.

Most of the resorbable elastomers described above are not yet commercial, which has limited their use in resorbable medical devices to proof-of-concept studies until now. However, a variety of chemical structures are possible to tune their mechanical and degradation properties. Depending on the materials, compression moduli, tensile strength, and elongation at break have been reported to range from 0.025 to 400 MPa, 0.2 to 2,500 MPa, and 10% to 2,500%, respectively (Turner et al., 2022). These materials typically degrade in a few weeks in water (Turner et al., 2022), due to the hydrolysis of ester bonds in saline media, accelerated in a basic medium, and by the action of endogenous esterases. Resorbable elastomers can also be combined with other materials such as bioplastics (PLA, PLGA, PCL, etc.) to obtain copolymers with intermediate mechanical or degradation properties. Elastomers do not possess the viscoelastic properties of soft tissues (Figure 2). However, they are particularly interesting in interfacing with highly stretchable tissues, such as skin, and are extensively used in the field of wearable bioelectronic systems and “skin electronics” (e-skin) (Park et al., 2022a; Gong et al., 2022; Liu et al., 2023a). Their mechanical toughness makes them also interesting when in contact with moderately “hard” tissues, like the tendons and heart. Therefore, it can be foreseen that resorbable elastomers will attract more and more interest in the coming years.

#### 2.1.2 Synthetic polyesters and other polymers

Thanks to their biocompatibility and well-controlled structure-tunable degradability, synthetic aliphatic polyesters have been extensively developed in medical devices since the 1970s. In particular, they have been used as surgical sutures, drug delivery systems, and tissue-engineering scaffolds. Most employed materials include PLA, poly(glycolic acid) (PGA), PLGA, PCL, and PHA (Figure 3). Because these materials generally display thermoplastic properties and can also serve as alternatives to petro-sourced polymers in other applications such as packaging, they have also been described as “bioplastics” (Bano et al., 2018; Jiang et al., 2022).

PLA is obtained by the poly(condensation) of lactic acid, which can interestingly be obtained by the bacterial fermentation of carbohydrates or synthetically. The L-lactide isomer is naturally produced and leads to PLLA (poly(L-lactide)). PLLA is quite a hydrophobic and slow-degrading crystalline polymer, with a glass transition temperature of 60°C–65°C and melting temperature of 175°C (Nair et al., 2007). It displays a relatively high tensile strength (0.01–5 GPa) (Eglin et al., 2008; Ulery et al., 2011) and a high modulus, with resorption kinetics up to 5 years (Ulery et al., 2011), making it suitable for use in surgical sutures. To increase its kinetics of resorbability, the two isomers, D and L, can be combined to obtain amorphous PLA with less mechanical strength and a lifetime of 12–16 months (Nair et al., 2007). Lactic acid can also be combined with glycolic acid to obtain PLGA. PLGA polymers are extensively used in medical applications since they can be processed with a wide range of different shapes: micro-structured films (Abu Ammar et al., 2021), porous scaffolds (Pan et al., 2012), microspheres and nanoparticles (Lu et al., 2023a), and microfibers (Chor et al., 2020). They can be used for drug delivery and tissue engineering applications or as surgical sutures or substrates for bioelectronics. Indeed, by modulating not only the L/G monomer ratio but also the polymer molecular mass and ending groups, it is possible to fine-tune the thermal, mechanical, and degradation properties of PLGA to make them match with the targeted application (Bano et al., 2018; Lu et al., 2023a). Contrary to PLA and PGA, PLGA are amorphous polymers when the L/G ratio is between 1/3 and 3, with fast kinetics of hydrolysis degradation up to 1–2 months for the 1/1 copolymer (Ulery et al., 2011). PGA is less hydrophobic and resistant against hydrolysis than PLA, but still crystalline and with a high melting temperature (>200°C), as well as displaying very high tensile strength (0.3–0.9 GPa) (Eglin et al., 2008; Ulery et al., 2011), which has made it a relevant material for surgical sutures and tissue engineering.

Poly(caprolactone) (PCL) is a hydrophobic semi-crystalline polymer that is interesting for its low glass transition (−54°C) and melting (approximately 60°C) temperatures, and its high elongation (300%–4,700%) and tensile strength (20–40 MPa) at break (Eglin et al., 2008; Ulery et al., 2011). It displays a very long degradation time (2–4 years). Because of its high permeability, it is mainly used in drug delivery systems and tissue engineering (Bano et al., 2018). PCL is also extensively used in combination with PLLA, PLA, and PLGA.

Poly(hydroxyalkanoates) (PHAs) are a family of polyesters produced by bacterial fermentation or algal bioproduction and presenting a very wide variety of structures and properties. To date, PHAs used in the biomedical field are mainly poly(3-hydroxybutyrate) (PHB) and poly(3-hydroxybutyrate-co-3-hydroxyvalerate) (PHBHV) (Singh et al., 2019; Tebaldi et al., 2019; Ansari et al., 2021). Their biocompatibility, non-immunogenicity, and non-carcinogenic properties have been regularly pointed out. Their mechanical properties can be tuned to match those of very soft (skin) to very hard (bone) tissues. Their kinetics of degradation can be tuned from weeks to several years, for short- or long-term drug delivery or implant use. Present biomedical applications of PHA include resorbable surgical sutures (muscle and skin regeneration) (Piarali et al., 2020), cardiovascular stents, bone and cartilage implants, or nerve repair conduits. In addition to tissue engineering applications, their use as drug delivery systems has also been explored (Koller, 2018; Singh et al., 2019; Tebaldi et al., 2019; Piarali et al., 2020; Ansari et al., 2021).

Bioplastics have been extensively used in resorbable bioelectronic applications, mainly as substrate films, eventually conformable and stretchable, but they can also be processed as fibers. For instance, electrospun PLA or PCL fibers coated with poly(aniline) or gold nanoparticles, eventually assembled in mats, have been used in cardiac tissue engineering (Fleischer et al., 2014; Wang et al., 2017a) and PCL fibers embedded with graphene and carbon nanotubes for nerve reconstruction (Sun et al., 2021). If bioplastics present tunable thermal and mechanical properties, as well as kinetics of resorbability, they remain rather hydrophobic materials. They can be combined with more hydrophilic polymer segments such as poly(ethylene glycol) (PEG), poly(vinyl alcohol) (PVA), and polyvinylpyrrolidone (PVP) to create copolymers. PEG, PVA, and PVP are synthetic polymers that can form after chain cross-linking hydrogels, i.e. 3D cross-linked polymer networks that can encapsulate large quantities of water. Because of their important swelling in aqueous buffers and biological fluids, hydrogels constitute ionic conductive materials when wet (Chen et al., 2021). As such, hydrogels also constitute materials of choice to mimic the tissue extracellular matrix (ECM). PEG, PVA, and PVP polymers are resorbable in the sense that they can be excreted through urine when their molecular mass is not too high (Yamaoka et al., 1994; Jensen et al., 2016; Kurakula et al., 2020). However, pure cross-linked PEG networks could present limited resorbability and potential safety concerns (Ulery et al., 2011). PVP and PVA are highly soluble in water and, therefore, are rather used as formulation aids but not often as scaffolds by themselves. If not cross-linked, they dissolve very quickly in water.

#### 2.1.3 Natural polymers

Natural polymers comprise mainly polysaccharides and protein-derived macromolecules.

Polysaccharides have aroused large interest due to their high availability, biocompatibility, variety of structures, and chemical and biological properties that they offer (Yang et al., 2022). In particular, hyaluronic acid (HA) is a highly relevant material for bioelectronics because it is an endogenous glycosaminoglycan of the extracellular matrix (ECM), which is widely available today as it is being produced by controlled bacterial fermentation, is easily processable (high water solubility and functional groups amenable to chemical modification on the polymer backbone), and is already being used in numerous biomedical applications (Knopf-Marques et al., 2016; Kobayashi et al., 2020; Vasvani et al., 2020). HA contributes to maintaining homeostasis and promotes cell migration, adhesion, and differentiation and as such has long been used in drug delivery systems and tissue engineering, in particular when in contact with the brain tissue (Miyata et al., 2017). HA is fully resorbable, with a degradation rate of a few hours to days (according to the body location) when not cross-linked. Other polysaccharides of interest for medical applications are chitosan which presents mucoadhesive properties (Dash et al., 2011), cellulosics (Aghazadeh et al., 2022), and alginate (Zhang et al., 2023). Though they display high biocompatibility and are degradable, only a few of their derivatives, such as oxidized cellulose and oxidized alginate, appear resorbable. Nevertheless, a few alginate-based materials will be described below since they were largely used to develop and illustrate innovative concepts, particularly in the field of dynamic hydrogels (Tringides et al., 2021; Tringides et al., 2023).

Another class of natural polymers is protein-derived macromolecules, such as collagen and its gelatin derivative, fibrin, elastin, elastin-like polypeptides, and silk fibroin. Similar to HA, collagen, fibrin, and elastin are endogenous components of the ECM. This confers to these materials mechanical properties that are very close to that of native tissues and a high biocompatibility although they can also elicit an immune response (Ulery et al., 2011). However, their supply can be limited by their extraction from animal sources and their cost (Wang et al., 2023). Mainly, silk has been explored for the design of resorbable bioelectronic systems, while collagen and gelatin have been used to design conductive hydrogels for tissue engineering.

Silk is a natural material produced by a variety of arthropods; the one extracted from the cocoons of *Bombyx mori* has been used for centuries and is still the most exploited due to its extraction by simple processes (Ullah et al., 2019). After processing, the major components, silk sericin and silk fibroin, are obtained. Fibroin, a structural protein composed of 18 amino acids, is constituted of crystalline β-sheets that self-assemble through intramolecular and intermolecular interactions (such as H-bonds, van der Waals, and hydrophobic interactions) and of hydrophilic and amorphous random coil domains. Playing with processing conditions that can tune the rearrangement and the ratio of the crystalline and amorphous domains, silk fibroin can be processed as nanofibers, microfibers (that can be converted to yarns and textiles), films, aerogels/cryogels, and hydrogels (Wang et al., 2019a). Its protein nature confers the material with high biocompatibility and programmable degradability and opens the possibility to further genetically engineer the protein (e.g., insert elastin-like sequences to improve elasticity for instance) to match the desired properties (Wang et al., 2021). The proteolytic degradation of silk fibroin films by enzymes such as chymotrypsin, actinase, and carboxylase can be accelerated by reducing the protein β-sheet content through the presence of chaotropic agents or the drying process (Cao et al., 2009; Chatterjee et al., 2019; Ullah et al., 2019). Because of its high biocompatibility and processability, the absence of adverse immune reaction, its resorbability, and outstanding mechanical toughness, silk fibroin has been employed for centuries in biomedical applications, for instance, as surgical sutures, besides its use in the textile industry.

Silk films were identified as early as the late 2000s as resorbable substrates of high interest for the design of soft electronics, particularly in the group of Rogers (Kim et al., 2009; Kim et al., 2010; Hwang et al., 2012; Tao et al., 2014). Transient electrode microarrays were designed by transfer printing very thin silicon patterns onto a casted 5- to 15-µm-thick silk fibroin film (Kim et al., 2009; Kim et al., 2010; Hwang et al., 2012). The very soft material showed high conformability to adapt to brain morphology and resorbability in water of approximately 1 h. Silk was also combined with resorbable magnesium [Mg (conductive) and MgO (insulating)] materials to develop a resorbable implanted system for the on-demand delivery of antibiotics to treat infected surgical wounds (Tao et al., 2014). The high Young’s modulus (5–12 GPa) and low stretchability (20%) of silk fibroin films were modulated by Chen et al. (2018a) in order to obtain stretchable films suitable for on-skin electronics. The authors studied the plasticization of a fibroin film through the addition of CaCl_2_ and ambient hydration, guided by molecular dynamics simulations of the rearrangement of β-sheets and coil domains into the film structure. After patterned vacuum deposition of gold, electrodes were obtained onto a wrinkled highly stretchable (>400%) film (Young’s modulus 0.1–2 MPa), and the device was tested for skin interfacial impedance measurement.

Silk fibroin can also be used to design resorbable conductive inks. Graphene derivatives and silk fibroin are particularly interesting to combine thanks to hydrogen bonds that can be reversibly created between the two materials (Wang et al., 2021). For instance, Dorishetty et al. (2022) studied the formulation, microstructure, and biocompatibility of a set of extrusion printable silk/graphene inks with different rGO content. An increase in the rGO concentration was shown to decrease the obtained material pore size while increasing its mechanical resistance. A PEDOT-based conductive printable ink was also developed by the addition of photosensitive sericin in an aqueous dispersion of PEDOT:PSS (Pal et al., 2016) (Figure 5). Silk sericin was modified with photosensitive methacrylate groups and mixed with PEDOT:PSS conductive ink. The photosensitive conductive resin was then spin-coated onto the fibroin substrate and UV-exposed through a photomask to create micropatterns after development in water.

The above examples demonstrate the high versatility and processability of silk that make it a real asset in the design of resorbable conductive materials. Similar to bioplastics, silk displays programmable structure-related degradability and mechanical properties and can moreover form optically transparent films for optoelectronic applications. As a natural polymer, silk is also a highly biocompatible and sustainable material, processable in water, and amenable to chemical modifications that can improve its interaction with conductive materials or tissue adhesion.

We have highlighted in this section the wide range of substrates/scaffolds that can be used to design resorbable materials. In the next section, we will focus on the electrical conductors.

### 2.2 Electrical conductors

**FIGURE 4:**
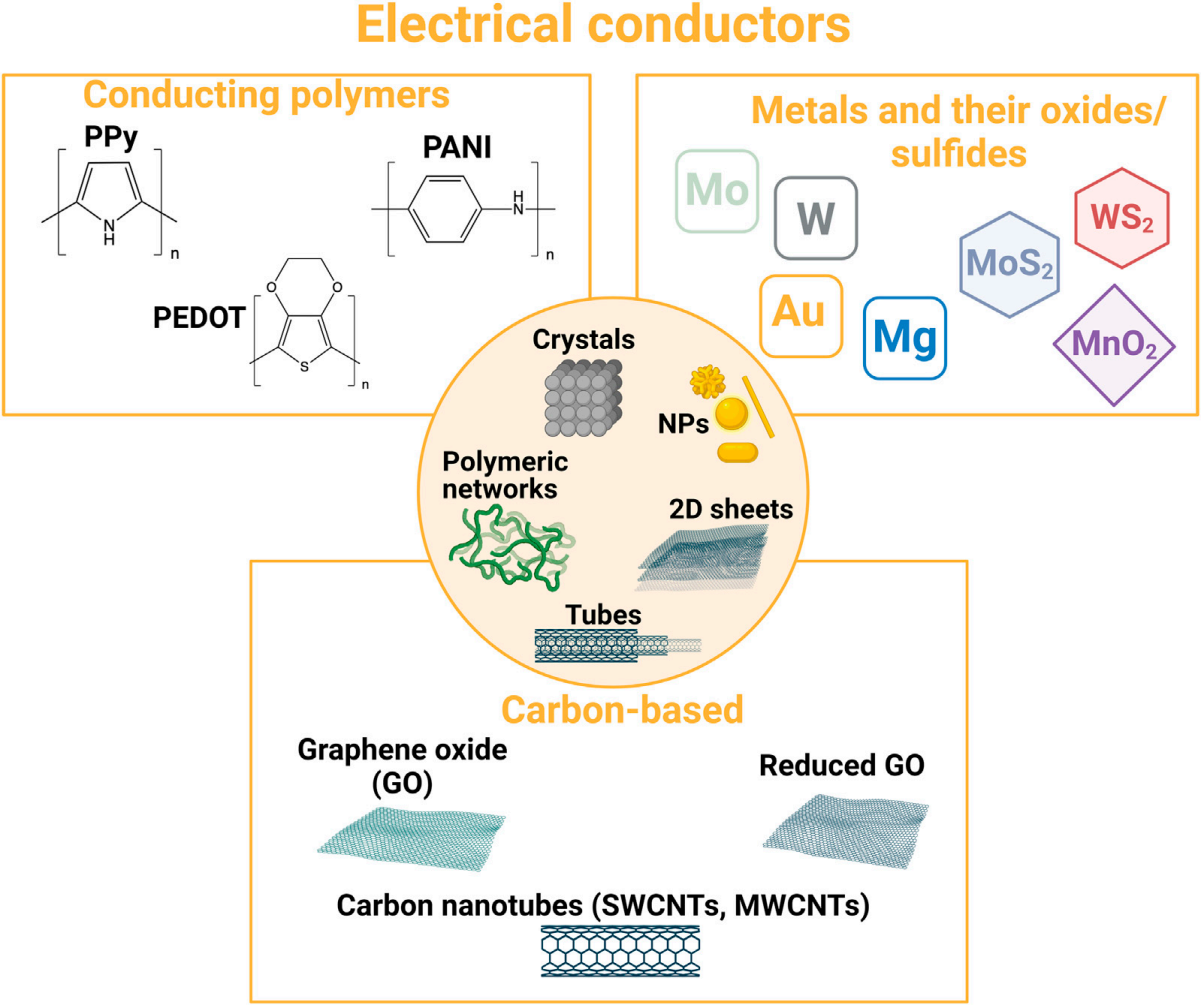
Overview of typical resorbable electrical conductors used in the design of bioresorbable conductive materials.

Different conductive components can be employed to design resorbable materials for healthcare applications (Figure 4). Metals and semiconductors are the electrical conductors that are usually employed in microelectronics. Therefore, when resorbable, they have been used to design resorbable bioelectronic systems dedicated to wearable or *in vivo* sensing or stimulation. To address tissue engineering applications and design 3D medical devices, such materials are employed in micro- or nanoparticle forms. Other micro- and nanostructures can also be used, and transition-metal dichalcogenides and carbon-based fillers (carbon nanotubes and graphene derivatives) are very popular in the field, either for the design of bioelectronic systems or tissue engineering. Conducting polymers are also very relevant materials for the bioelectronic system/tissue interface, either for the design of wearable bioelectronic systems or tissue engineering. Their strengths are their mixed ionic/electronic behavior and their polymeric nature, making them both a structuring and conductive material.

#### 2.2.1 Metals and semiconductors

Classically employed materials for designing conductive patterns in microelectronics are semiconductors or metals. Several are biocompatible (platinum, gold, titanium nitride, silicon, etc.), while some are resorbable (molybdenum, magnesium, tungsten, iron, silicon, germanium, and zinc/zinc oxide) (Chatterjee et al., 2019). Their resorbability is mainly accounted for by the formation of metal hydroxides or oxides that are dissolvable in biofluids. The kinetics of bioresorption of resorbable metals can vary over different orders of magnitude, from 1.7 µm/day at pH 7 at 37°C for Mg to 7 nm/day for Mo (Chatterjee et al., 2019). Silicon hydrolysis depends not only on pH, temperature, and ionic concentration but also on its crystalline form (Hwang et al., 2012). Although these materials are inherently hard in bulk, they can be deposited on a flexible substrate in thin layers, of the order of a hundred nanometers, or can be thinned after deposit to obtain flexible bioelectronic systems. Metals and semiconductors can also be shaped into nanostructures such as nanotubes or nanowires to increase the contact surface with tissues (Duan et al., 2012; Robinson et al., 2012). Combining resorbable polymer substrates and metals, different research groups have started developing resorbable electronic systems since the late 2000s. For instance, the group of Rogers has extensively developed the electronic on-silk concept (Kim et al., 2009; Kim et al., 2010; Hwang et al., 2012; Tao et al., 2014). Huang et al. (2014a) developed a fully printed circuit board using poly(ethylene oxide) and carboxymethyl cellulose as substrate materials combined with magnesium stacks and a paste comprising tungsten microparticles for electrical connections. Still, resorbable polyester substrates such as PLGA films are presently the most employed.

The high interest of semiconductors and metals is their high intrinsic conductivity. However, it could be complicated to combine them in bulk with highly porous and hydrophilic materials such as hydrogels. Choi et al. (2020) described the possibility of combining hydrogels with a liquid metal, an eutectic alloy of 75% gallium and 25% indium (mass ratio). Though the biocompatibility of liquid metal Galinstan, an eutectic mixture of 68% Ga, 22% In, and 10% Sn (mass ratio), has been assessed (Foremny et al., 2021), the use of such materials still remains questionable from the biocompatibility and resorbability points of view. Another possibility to combine metals and other conductive materials to resorbable scaffolds is their use as micro- or nanostructures.

#### 2.2.2 Conductive nanostructures

A large variety of inorganic conductive micro- and nanostructures are available to design conductive materials (Figure 4). Micro- and nanostructures include semiconductor or metallic flakes, nanowires or nanorods, 2D materials like transition metal dichalcogenides (MoS_2_ and WS_2_), Xenes (e.g., black phosphorous), MXenes (2D carbides or nitrides), transition metal oxides (e.g., MnO_2_ and MoO_3_), and carbon-based structures, such as carbon nanotubes and graphene-based materials. The increased surface/volume ratio facilitates and fastens the material resorption in the body, when possible. We will shortly focus below on the few structures that have been highlighted in literature and for which more solid resorption data exist. Numerous other conductive micro- and nanomaterials are presently under development and could be acknowledged in the near future as suitable for the design of resorbable bioelectronics (Choi et al., 2019).

Concerning metallic nanostructures, because of biocompatibility issues, copper and silver are ruled out for most biomedical applications (Han et al., 2022). The *in vivo* safety of platinum nanoparticles (Pt NPs) has also been questioned (Czubacka et al., 2019; Gutiérrez de la Rosa et al., 2022). As a highly stable metal in biological fluids, Pt NPs require a very long time for dissolution-based resorption but can possibly be eliminated by urinary excretion. Because of their high biocompatibility and processability, gold nanoparticles, nanocages, nanorods, and nanowires are extensively studied in tissue engineering applications (Yadid et al., 2019), especially cardiac engineering (Saghebasl et al., 2022). However, they are not bioresorbable, even if very recently, small gold-polymer nanostructures (90 nm size, 4.5% w/w gold) were found to be excretable (Cassano et al., 2019), 4–22 nm-diameter gold nanostructures were also found to be degraded and metabolized by cells by similar pathways than gold ions, but into biopersistent products (Balfourier et al., 2020).

Two-dimensional transition-metal dichalcogenides, such as molybdenum disulfide (MoS_2_) sheets, are very attractive thanks to their 2D electrical conductivity and optical transparency in the visible range that can be used advantageously in optoelectronic systems (Choi et al., 2019). The resorbability of MoS_2_-isolated crystals and large-area polycrystalline films in representative fluids and cranial environments was studied (Chen et al., 2018b). The polycrystalline MoS_2_ monolayer (grain size ∼200 nm) dissolved in approximately 2 months in PBS solution at 37°C, and the CVD-grown monolayer inserted in implantable device prototypes was dissolved completely in 1 month. Such implantable bioelectronic systems can be used to monitor pressure, strain, and temperature (Chen et al., 2018b), as well as serve as an image sensor array when implanted in the eye, thanks to their photoabsorption and photocurrent generation performances (Choi et al., 2017).

Pristine graphene is also a 2D material of high interest for bioelectronic applications (Choi et al., 2019). Because of the thickness of individual layers (a few angstroms), graphene can be soft and flexible, yet strain-resistant and optically transparent. Based on a hexagonal honeycomb lattice of carbon atoms, graphene is also biocompatible and can be functionalized easily for better interactions with tissues or other materials though its impact on the immune system is being questioned (Ban G et al., 2023). Endogenous peroxidases such as human myeloperoxidase can degrade graphene in the form of a single or a few layers, in tens of hours to days (Ma et al., 2020). However, pristine graphene is quite hydrophobic and hence poorly compatible with hydrogels (Chen et al., 2021). Graphene oxide (GO), more hydrophilic and dispersible in water, displays poor conductivity because of the damaged conductive network due to the oxidation of the graphene structure. Therefore, reduced graphene oxide (rGO), for which the graphene structure has been partially restored, constitutes a good compromise between conductivity and limited hydrophobicity and is preferred for biomedical applications (Chen et al., 2021). The kinetics of degradation of GO and rGO is related to the oxygen atom content of the structures, and therefore they resorb in the body with faster kinetics than pristine graphene (Ma et al., 2020).

Carbon nanotubes (CNTs) also constitute a hexagonal lattice of carbon atoms rolled up into a tube of 0.5–2 nm diameter for single-walled carbon nanotubes (SWCNTs), with several SWCNTs in a tube-in-tube structure for multi-walled carbon nanotubes (MWCNTs). Like graphene, their carbon atoms can be oxidized and functionalized to confer additional functionalities and hydrophilicity. For instance, Liu et al. (2011) studied *in vitro* the influence of carbon nanotube surface chemistry (carboxylic acids, amines, and alcohols) on neuron network organization, offering cells a high variety of adhesion orientation and sites. They have been particularly explored in wearable sensor applications (Gandhi et al., 2020; Palumbo et al., 2022) and nerve constructs for tissue engineering (Salehi et al., 2018; Manousiouthakis et al., 2022). Though resorbable, thanks to their degradation by peroxidases (Ma et al., 2020), their biocompatibility is questionable (Mishra et al., 2018). It seems to mostly depend on their synthesis and purification process, as well as their aspect ratio.

#### 2.2.3 Conducting polymers

Electronic conducting polymers (CPs) such as poly(pyrrole) (PPy), polyaniline (PANI), poly(thiophene) (PTh), and poly(3,4-ethylenedioxythiophene) (PEDOT) are a class of polymers that possess π-conjugated structures, enabling electron delocalization along their backbone (Guo et al., 2020). Given the aromaticity of the polymer structure, the overlapping of π-orbitals results in a conductive pathway obtained by freely moving delocalized π-electrons (Nezakati et al., 2018). In their pristine state, CPs behave as semiconductor materials exhibiting weak conductivity compared to classical semiconductors. When oxidized (0.25–0.33 charge per monomeric unit) through electrochemical treatments or oxidation/reduction chemical reactions, they gain high electronic conductivity. This (electro)-oxidation process is accompanied by the introduction in the CP films of counter anions that maintain their electroneutrality. Such an ion exchange property is generally referred to as a doping process correlatively to inorganic semiconductors, the counter anions being referred to as the dopant species (Namsheer et al., 2021). As such, conducting polymers are a category of organic materials characterized by distinctive electrical and electrochemical performances. They combine the advantages of a wide range of electrical conductivity (10^−5^–10^5^ S cm^−1^) like carbon- or metal-based electrical conductors and of structure tunability, flexibility, and relatively low cost, like polymers. They also feature both ionic (such as tissues) and electronic (such as bioelectronic systems) conductivities, making them ideal interface materials for bioelectronic applications (Fattahi et al., 2014; Onorato et al., 2019; Gao et al., 2022). CP coatings on metallic electrodes can significantly enhance the electrical properties at the interface of tissues as compared to bare electrodes, especially decreasing electrical impedance and increasing charge transfer capacity (Ludwig et al., 2006; Richardson-Burns et al., 2007; Khodagholy et al., 2015). For instance, conducting polymer microstructures (microcups) were obtained by the electrochemical deposition of conducting polymers (PEDOT, PPy) onto the surface of PLLA/PLGA sacrificial microspheres (Antensteiner et al., 2017a; Khorrami et al., 2017). These microstructures deposited on the surface of a metallic electrode enhanced the surface roughness of the CP films by over 90% through the control of both deposition time and applied electrical voltage. The impedance of PPy-modified electrodes was found to decrease by up to 88% in comparison to bare electrodes (Antensteiner et al., 2017b). These neural electrodes improved the cellular response of neurons during chronic stimulation and recording (Antensteiner et al., 2017a; Khorrami et al., 2017). Thanks to these unique combined properties, as well as their ease of preparation and biocompatibility, conducting polymers have attracted significant attention for a wide range of biomedical applications such as the development of (bio)-sensors, actuators, drug delivery systems, and tissue engineering scaffolds. Due to their availability and physiologically relevant electrical conductivity upon intrinsic or external doping, PPy, PANI, PEDOT, and their derivatives, are the most widely used CPs for bio-interfacing applications.

Poly(pyrrole) (PPy) is a heterocyclic polymer that can be easily synthetized in different solvents (such as water) and polymerized via chemical or electrochemical routes of pyrrole oxidation (Mao et al., 2018). Due to its high biocompatibility, ease of preparation, and good conductivity in physiological conditions, it has been widely used at the interface between bioelectronic systems and cells or tissues, for both recording and stimulation purposes (Liu et al., 2023b). Notably, PPy is a suitable substrate in the modulation of different cellular activities (such as cell attachment and proliferation) and possesses excellent biocompatibility *in vivo* (Huang et al., 2014b). Furthermore, it can be easily chemically modified to allow the conjugation of bioactive molecules such as proteins and enzymes (e.g., glucose oxidase and antibodies), short peptides with specific “recognition sequences” (providing binding sites for cells), nucleic acids, DNA/RNA, or growth factors (which allow the immobilization of cytokines). These bioactive molecules can act as PPy dopants, improving material conductivity and/or further promoting material–tissue integration. Nevertheless, additional polymer modifications are required to overcome issues with water solubility, mechanical rigidity, and poor processability, which significantly limits its use as a standalone material. In addition, PPy is very sensitive to over-oxidation in the presence of radical oxygen species present in biological media or generated by local inflammatory processes (Palmisano et al., 1995). Despite its considerable biocompatibility, at its pristine state, PPy is also poorly degradable. Bioerodible forms of PPy can be prepared via electrochemical polymerization of beta-substituted pyrrole monomers containing hydrolyzable side groups (Zelikin et al., 2002) or by PPy integration in biodegradable polymers, such as PLA or PCL (Boutry et al., 2012).

Polyaniline (PANI) is a phenylene-based polymer offering several advantages in biomedical applications, such as high thermal and environmental stability, high conductivity values (10^1^–10^2^ S cm^−1^), and inexpensive and easy synthesis processes (Solazzo et al., 2019). It can be synthesized via electrochemical processes, or chemical oxidation of aniline monomer, typically in the presence of ammonium persulfate as the oxidizing agent. Fine control over synthesis conditions, such as pH, the presence of acids, and the choice of solvents and oxidizing levels, greatly influence the physical and electrical properties of the obtained PANI polymers that can be under different forms (i.e., leucoemeraldine, emeraldine, and pernigraniline) that differ by the oxidation level of the backbone (Tran et al., 2022). PANI generally offers several advantages for biomedical applications, such as high thermal and environmental stability, antibacterial properties, inexpensive and easy synthesis processes, and excellent charge transport due to the doping/de-doping process (Beygisangchin et al., 2021). Another major advantage of PANI is its good solubility in a few selected organic solvents, as well as in water, thereby largely improving material processability. Nevertheless, the biocompatibility of both pristine PANI and its derivatives is still controversial. Several studies have reported high *in vitro* cytotoxicity and chronic inflammation episodes after cell/tissue contact (Wang et al., 1999; Borriello et al., 2011; Zhang et al., 2019), despite a plethora of studies having reported good *in vitro* and *in vivo* compatibility of PANI oligomers (pentamers and tetramers) when employed in subcutaneous films or biomimetic sensors for short-term use (Humpolicek et al., 2012; Guo et al., 2018; Pyarasani et al., 2019). Furthermore, similar to the other conducting polymers, PANI used as a standalone material is also non-degradable. As well as for PPy applications, in recent years, numerous studies have focused on combining PANI with other biodegradable synthetic or natural polymers to develop blends or composite systems (Rai et al., 2022). Despite this, *in vivo* studies assessing the resorbability of PANI-based materials or their long-term presence in the body (eventually in degraded form) have not been extensively carried out.

Poly(3,4-ethylenedioxythiophene) (PEDOT), a thiophene derivative of the less stable and less biocompatible pristine poly(thiophene), is one of the most widely studied CPs in bioelectronics and tissue engineering applications due to its improved chemical and environmental stability and biocompatibility compared to PPy and PANI (Tropp et al., 2021). PEDOT is generally synthetized in the presence of a dopant, typically polystyrene sulfonate (PSS), which plays the role of the counter anion of the positively charged PEDOT (more conductive than the pristine form) and improves polymer solubility and conductivity (Gueye et al., 2020). Although poly(styrene sulfonate) (PSS) is the most widely studied PEDOT dopant to design soft flexible conductive materials for bioelectronics, PSS lacks biocompatibility and degradability (Kayser et al., 2019). Several biomolecules have therefore been investigated to obtain resorbable PEDOT:biomolecule inks to be used for the design of transient implantable electronic devices (Boehler et al., 2019; Leprince et al., 2023a). PEDOT is generally obtained via three polymerization routes: i) oxidative chemical polymerization of EDOT-based monomers using various oxidants, yielding a suspension of PEDOT (or PEDOT:dopant) particles (ink) (Nie et al., 2021; Leprince et al., 2023a), ii) electrochemical polymerization of an EDOT-based monomer onto a conductive substrate in a three-electrode setup yielding a PEDOT coating or a film, and iii) using transition-metal-mediated coupling (Nie et al., 2021). When not electrochemically synthetized onto a substrate or as a self-standing film, PEDOT can be formulated to be further coated, casted, or printed by different processes (spin-coating, dip-coating, spray coating, screen printing, inkjet printing, doctor blading, roll-to-roll printing, etc.). The relative ease of PEDOT (or PEDOT/dopant) processing and the possibility to design it as resorbable using biomolecule-based dopants have led to the widespread use of PEDOT in the development of modified electrodes for tissue interface (Wang et al., 2019b) and tissue engineering applications (Bhat et al., 2021).

Despite CPs promising bioelectrical characteristics and ease of processability, the same intrinsic structure enabling conductivity is also characterized by strong bond dissociation energies, imparting the materials with excessive rigidity and poor degradability, limiting their application as biointerfaces with tissues (Liu et al., 2023b). CPs’ conjugated backbones are indeed hardly cleavable in physiological conditions, unless for small molecular mass, resulting in a poor to null degradation and metabolization in the body. Furthermore, several classical dopants, such as PSS for PEDOT, are not cleavable in the physiological condition as well, further hindering CPs' *in vivo* employability. Replacing PSS by potentially (bio)degradable molecules, such as alginate (Puiggalí-Jou et al., 2020; Yang et al., 2020), heparin (Xu et al., 2019b), or hyaluronic acid (Guo et al., 2018; Leprince et al., 2023a) is a widely known strategy to increase PEDOT:dopant biocompatibility and degradability, preserving the inherent conductivity of the polymer (Guo et al., 2013). Interestingly, such biopolymer-based dopants can also be used to design the conductive substrate/scaffold part of the conductive material. If resorbable and associated with CP oligomers, resorbable conductive hydrogels can be obtained with high interest in bioelectronics and tissue engineering. A second reported strategy is the integration of cleavable chemical motifs in the CPs' backbone, which allows their partial or complete degradation into smaller products (monomer or oligomer fragments) that can be processed through different physiological mechanisms, such as phagocytosis, metabolization, bioabsorption, or excretion (Feig et al., 2018). Disintegrable organic semiconductors can also be obtained by utilizing degradable or reversible dynamic covalent linkages, for instance, based on Schiff base chemistry, within each conducting polymer repeat unit (Tropp et al., 2021). The use of hydrolyzable linkages within a CP monomer leads to a degradable conducting polymer backbone that affords the design of high-performance transient and biocompatible semiconductors (Lei et al., 2017). In conclusion, extensive and rigorous studies on the mid- and long-term use of CP-based materials in transient implantable devices are still required to solve the pending questions of device biocompatibility and electroconductive stability over time. However, such materials display high structural versatility and offer large possibilities and interest in bioelectronic applications, especially on-skin electronics and tissue engineering.

Only a limited number of electrical conductors inherently exhibit full bioresorbability such as resorbable metals, semiconductors, and MoS_2_ sheets. However, the functionalization of others (such as graphene, carbon nanotubes, and their derivatives) through the application of organic coatings or the combination of conductive oligomers with bioresorbable polymers has paved the way for enhancing these materials' biocompatibility and resorbability. Consequently, strategies that amalgamate structuring scaffolds with electrical conductors play a pivotal role in achieving resorbable conductive materials.

## 3 Combining structuring scaffolds and electrical conductors

**FIGURE 5:**
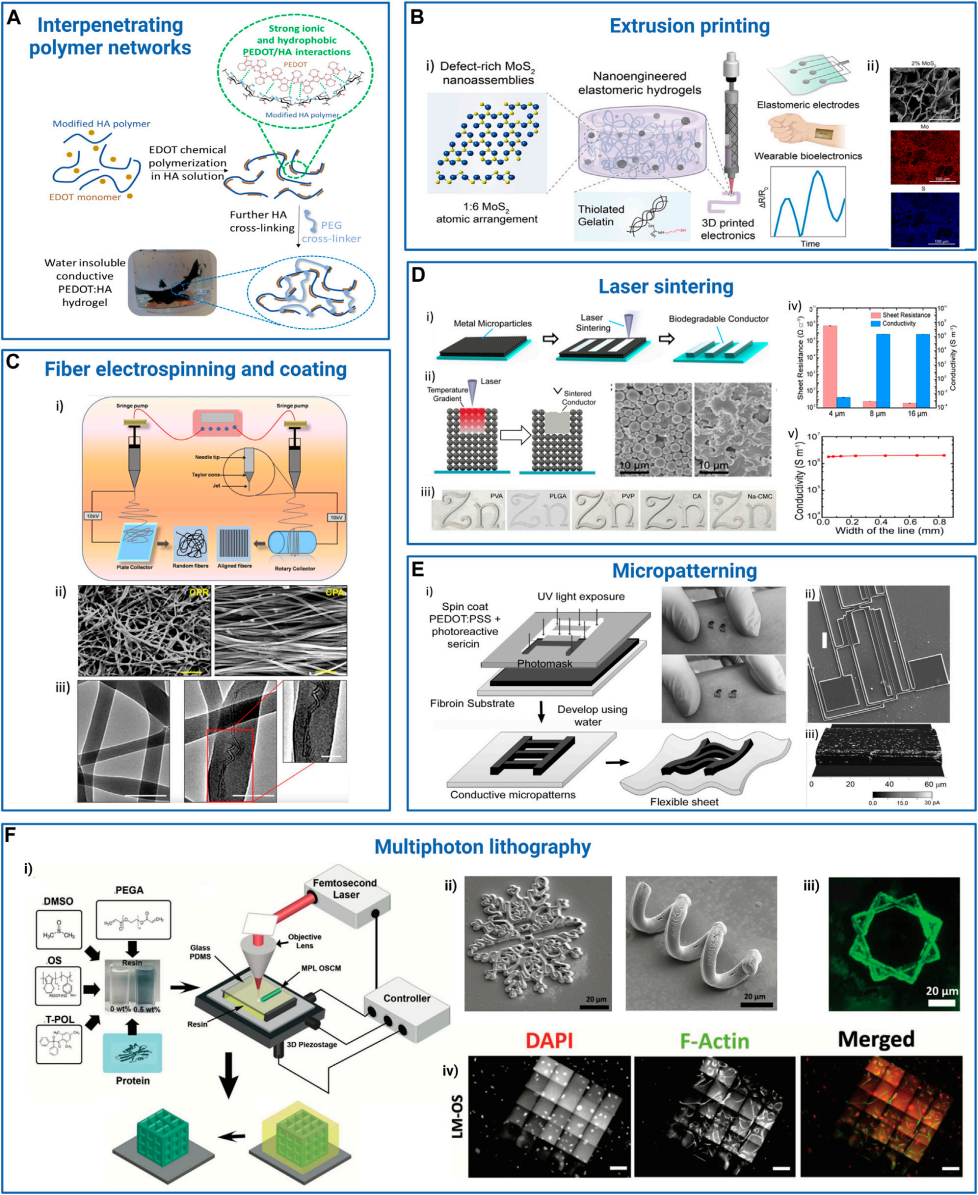
Examples of processes and strategies used to obtain resorbable conductive materials for their application in bioelectronics. **(A)** *Interpenetrating networks of scaffolding and conducting polymers*: conducting polymer PEDOT is chemically synthetized in a solution of modified hyaluronic acid (HA) polymer. The PEDOT:HA ink can be further photo-cross-linked to achieve non-water soluble resorbable conductive hydrogels (Leprince et al., 2023b). Adapted with permission from Leprince et al. (2023a), Copyright 2023, Elsevier, and Leprince et al. (2023b), Copyright 2023, the Royal Society of Chemistry. **(B)** *Extrusion printing*: (i) a bioprintable conductive ink is obtained by mixing thiolated gelatin and defect-rich MoS_2_ nano-assemblies. This ink can be further extrusion-printed as a stand-alone material to design wearable sensors. ii) Scanning electron microscopy (SEM) and energy-dispersive X-ray spectroscopy (EDS) images of a transverse cross-section of the hydrogel: gelatin scaffold (top), molybdenum (Mo, middle), and sulfur (S, bottom). Scale bar: 100 µm. Reprinted with permission from Deo et al. (2022), Copyright 2022, the American Chemical Society. **(C)** *Fiber electrospinning and coating:* electrospun PCL-collagen fibers coaxially reinforced with MWCNTs. i) Electrospinning parameters were modulated to deposit random or parallel fibers. ii) SEM images of the random (CPR, left) and aligned (CPA, right) MWCNT-reinforced fiber scaffolds. Scale bar: 1 μm. iii) TEM images of scaffolds without (left) and with (right) MWCNT fibers. Scale bar: 200 nm, inset: 100 nm. Adapted with permission from Ghosh et al. (2020), Copyright 2020, the American Chemical Society. **(D)** *Laser sintering*: laser sintering process to integrate zinc or iron microparticle inks into hydrogels (i). The microparticle ink (black) was spin-coated onto the hydrogel substrate (blue), and the device was washed in the appropriate solvent after laser sintering to reveal the pattern. ii) Scheme illustrating the sintering process. Scale bar: 10 μm. iii) Examples of Zn patterns obtained on different polymer substrates [PVA, PLGA, PVP, cellulose acetate (CA), and sodium carboxymethyl cellulose (Na-CMC)]. iv and v) Resistance and conductivity of Zn line patterns with different thicknesses (iv) and widths (v) onto PVA substrate. Adapted with permission from Feng et al. (2019), Copyright 2019, the American Chemical Society. **(E)** *Photolithography micro-patterning*: i) scheme depicting the microfabrication of silk sericin/PEDOT:PSS patterns onto a silk fibroin substrate. ii) SEM image of a complex resorbable device (ii, scale bar: 100 µm). iii) Conductive AFM image of a 50-μm-thick ink line deposited on a glass surface. Reproduced with permission, Copyright 2015, John Wiley and Sons (Pal et al., 2016). **(F)** *Multiphoton lithography*: i) schematics illustrating the composition of the organic semiconductor (OS) resin, featuring PEDOT:PSS, photoinitiator (3-(trimethoxysilyl)propyl methacrylate), dimethyl sulfoxide (DMSO), laminin, glucose oxidase, and the experimental setup for multiphoton lithography, resulting in the formation of 3D OS composite microstructures (OSCM) upon resin removal (depicted in yellow). ii) SEM images showcasing diverse conductive and bioactive complex microstructures. Scale bar: 20 μm. iii) Fluorescent microscopy images depicting laminin-incorporated OS (LM-OS) microstructures in green. Scale bar: 20 μm. iv) Epifluorescence microscopy images showing endothelial cells on LM-OS microstructures after 48 h of culture, stained with DAPI (red, indicating cell nuclei) and phalloidin (green, representing F-actin). Scale bar: 100 µm. Reproduced with permission, Copyright 2022, John Wiley and Sons (Dadras-Toussi et al., 2022). Created with BioRender.com.

Based on the large variety of existing chemical structures for both resorbable substrates/scaffolds and electrical conductors, there exist different possibilities to combine them to obtain conductive materials for their use in bioelectronics or tissue engineering. We propose below an insight into the most popular processes encountered in the bibliography. Some of them are illustrated in Figure 5.

### 3.1 Blending structuring scaffolds/substrates and electrical conductors

Blending is the easiest way to combine synthetic or natural polymer-based substrates/scaffolds with electrical conductors such as metallic or semiconducting nanoparticles, nanorods or nanowires, carbon-based conductive fillers, and conducting polymers. We provide below general examples of blended resorbable conductive materials, preceding a brief section concerning polymer cross-linking that can be used to ensure material infusibility in tissues in contact with biological fluids and place specific enphasis on conducting polymer/hydrogel interpenetrated polymer networks.

#### 3.1.1 Blended resorbable conductive materials

Blending has been used to design several parts of resorbable bioelectronic systems.

Biodegradable conductive pastes were developed by blending tungsten micro-/nanoparticles with natural waxes (issued from bee, soy, myrtle, or candelilla) (Won et al., 2018; Kim et al., 2023a). These hydrophobic pastes, which degrade within days to weeks, can be used to interconnect different electronic components of resorbable bioelectronic systems (Won et al., 2018; Choi et al., 2021).

Resorbable PVA films filled with iron nanoparticles and MWCNT were used to design biodegradable tactile sensors (Karmakar et al., 2022). Wang et al. (2019c) designed a resorbable strain sensor based on a dynamic network of PVA, borate, and GO particles partially reduced during dopamine oxidative self-polymerization. The PVA-PDA-rGO material displayed self-healing features, and the rGO filler acted both as a mechanical stiffener and electrically conductive particles. rGO was also combined with PCL, PU, PEG, gelatin methacrylate (GelMA), collagen, and chitosan for cardiac (Saghebasl et al., 2022) or neural (Manousiouthakis et al., 2022) tissue engineering. Tringides et al. (2021) blended alginate hydrogels with both graphene flakes and carbon nanotubes prior to gelation and freeze drying to obtain macroporous materials. They thoroughly studied the impact of the two types of materials on the structure and conductivity of the obtained conductive hydrogels. They showed that graphene flakes were integrated into the walls of the porous materials, whereas CNT acted as interconnects through the pores, to achieve conductivities as high as 35 S m^–1^. The conductive alginate hydrogel (100 µm thick) could then be encapsulated between two thin layers (15 µm) of a resorbable viscoelastic elastomer in which openings could be performed with a CO_2_ laser and to design a resorbable electrode array for the heart or brain electrical recording or stimulation.

In tissue engineering applications, blending conductive fillers or conducting polymers into resorbable hydrogels is the usual method to obtain conductive ECM-like scaffolds. For instance, AuNPs have been integrated with different polymer-based scaffolds (alginate, chitosan, PCL/gelatin, etc.) and shown to improve the mechanical and electrical properties of the matrices and tissue regeneration from stem cells. PEDOT:PSS was mixed with collagen and (3-glycidoxypropyl)trimethoxysilane as a cross-linker to design 3D conductive scaffolds that can be used to measure impedance and monitor cell growth (Inal et al., 2017). In nerve tissue reconstruction, 2D graphene materials have often been combined with carbon nanotubes as polyester or hydrogel fillers (Gupta et al., 2019; Sun et al., 2021; Huang et al., 2023). The objective is to optimize the material conductivity by ensuring the percolation of the conductive domains, while minimizing the impact of these additives on the scaffold biocompatibility and/or mechanical properties.

Nano- and microparticles and 2D materials are interesting to blend with a polymer matrix to obtain resorbable devices for different reasons. Their size and dimensionality make them soft and suitable to be combined with flexible substrates to match the curvilinear shape of the human body and fasten and ease their resorption. In addition to their electrical properties, they can also confer the materials with additional functional properties, such as optical transparency, when at low concentration or present as a thin layer. The increased surface-to-volume ratio can also increase electric signal sensitivity to the adsorption of molecules or biomolecules. Therefore, the use of such materials is a real asset to design resorbable optoelectronic systems and sensors or biosensors. However, we have to consider that the presence of fillers generally modifies the material’s toughness and stretchability (most of the time, the material becomes harder), which can impact its interaction with tissues.

It is also noteworthy that it is necessary to achieve high payloads of electrical conductors within the polymer matrix to ensure high material conductivity, especially when dry materials, i.e., elastomer- or bioplastic-based substrates are concerned. Indeed, in that case, the electrical conductors have to percolate into the by-nature insulating matrix to ensure material conductivity. In this perspective, the blending strategy is particularly interesting to combine electrical conductors and hydrogels. The ionic conductivity of hydrogels and their porous structure potentially facilitating the percolation of electronic conductors, could offer advantages to achieve conductive materials with moderate electrical conductivities.

When synthetic or natural polymers are used as substrates/scaffolds, it is sometimes required that the polymer chains are cross-linked so that the material is resorbable in the mid to long term but remains infusible when in contact with biological fluids during its use. This is particularly true for hydrophilic polymer networks that are hydrogels. Polymer cross-linking strategies will be detailed in the following sections.

#### 3.1.2 Polymer cross-linking

Cross-linking by physical bonds like H-bonding and hydrophobic interactions, or chemical bonds, in particular covalent bonds, can be achieved through thermal, mechanical, or chemical treatments. A variety of functional groups natively present on synthetic or natural polymers can be exploited for chemical cross-linking, such as alcohols (-OH), thiols (-SH), carboxylic acids (-COOH), and amines (-NH_2_). Other chemical functions (such as alkenes, alkynes, acrylates, methacrylates, azides, hydrazides, hydroxyamines, etc.) can be introduced, notably to further use click chemistry-based cross-linking strategies that can be performed in water and limit the formation of potentially toxic by-products (Hu et al., 2019; Chiulan et al., 2021; Battigelli et al., 2022; Mueller et al., 2022). Popular chemical cross-linking reactions include Michael additions, Diels–Alder reactions, azide–alkyne cycloadditions, thiol-ene photo-reaction, or photo-initiated methacrylate polymerization. By the modulation of the nature of the polymer, polymer and cross-linker concentrations, and eventually the number of chemical groups per polymer chain (degree of substitution), the crosslink density and hence the porosity and the mechanical and swelling properties of obtained materials can be fine-tuned.

More recently, the limitations of purely elastic polymer networks (i.e., cross-linked with static covalent bonds) were underlined, especially in the field of tissue engineering (Chaudhuri et al., 2015; Chaudhuri et al., 2016; Yang et al., 2016; Morgan et al., 2022). Dynamic polymeric networks, especially hydrogels, were described that can offer self-healing behavior, and dynamicity and stress relaxation properties that exist in tissues (Elosegui-Artola, 2021). Dynamic hydrogels are also intensively explored in the field of wearable bioelectronic systems as outstanding reversible adhesives to interface electronics and skin. Hydrogel/tissue interface interactions can rely on electrostatic or hydrophobic interactions, topological adhesion, and irreversible or reversible (i.e., dynamic) covalent bonding (Cong et al., 2022). In particular, bioinspired approaches such as those based on catechol chemistry (mussel-inspired) allow the establishment of stable and high-strength adhesive interfaces using easy polymer modification by functional moieties such as catechol, dopamine, or tannic acid (Zhang et al., 2020). The conductive version of these adhesive hydrogels is of greatest interest as ideal electrode interfaces between electronic systems and tissues. Other typical dynamic linkages are boronic esters, disulfide bonds, and hydrazine and oxime bonds (Zhu et al., 2023b; Grosjean et al., 2023). In particular, hydrogels obtained by mixing fast equilibrium hydrazide cross-links (K_eq_ ≈ 10^3^–10^4^ L mol^–1^) and slow equilibrium oxime cross-links (K_eq_ ≈ 10^6^–10^8^ L mol^–1^) have aroused high interest in tissue engineering (Morgan et al., 2022). They can be easily obtained by mixing an aldehyde or a methyl-ketone–modified polysaccharide with a mixture of bis(hydrazine) and bis(oxyamine) cross-linkers, whose ratio can be tuned to obtain a series of hydrogels with a range of elastic and viscoelastic properties (Su et al., 2010; Koivusalo et al., 2019; Baker et al., 2021; Morgan et al., 2022).

Taking advantage of the polymeric structure of both conducting polymers such as PANI, PPy, and PEDOT and hydrogels, the above-described crosslinking strategies can be pushed further to interpenetrate the two polymeric networks (Rogers et al., 2020; Xu et al., 2020; Chen et al., 2021; Xu et al., 2021; Gao et al., 2022; Zhu et al., 2023a).

#### 3.1.3 Interpenetrated networks of conducting polymers and hydrogels

Conducting polymer hydrogels have garnered particular interest because it is possible to uniquely play with the polymer nature of both the CP and hydrogel scaffold to end with original molecular designs where the two networks are interpenetrated. Integrating CPs within the hydrogel matrix allows the generation of conductive hydrogels that combine the tissue–biomimetic characteristics of hydrogels, such as soft mechanical properties and high swelling ratio, minimizing mismatch at the interface with biological tissues, with the peculiar conductivity of CPs (Liu et al., 2023b).

CP/hydrogel interpenetrated networks (IPNs) can be obtained by different strategies (Xu et al., 2020; Liu et al., 2023b). In a one-step strategy, the conductive monomer and the scaffold precursors are mixed, then the *in situ* redox polymerization of the conductive monomer (for instance, EDOT) is conducted simultaneously to the reticulation/gelation of the polymer matrix. For instance, a PEDOT:alginate hydrogel to support the 3D culture of brown adipose-derived stem cells was obtained by mixing in one pot EDOT, alginate, ammonium persulfate (as EDOT oxidant), and adipic acid dihydrazide (as alginate cross-linker) (Yang et al., 2020). However, the one-step strategy requires that the scaffold precursors and the conductive monomer react simultaneously, or with orthogonal chemistries. In a two-step strategy, the conductive monomer is mixed within the already cross-linked scaffold and then oxidized chemically or electrochemically to obtain the conducting polymer intertwined within the scaffold matrix. EDOT was chemically polymerized in the presence of a chitosan/gelatin cross-linked scaffold for neural tissue engineering (Wang et al., 2017b). Despite that relatively high conductivity was obtained (0.17 S/cm), the authors first swelled the hydrogel for 3 h in ammonium persulfate buffer, before immersing it in an EDOT/hexane solution to perform EDOT polymerization, probably in order to avoid EDOT polymerization that only occurs at the hydrogel interface. Alternatively, scaffold precursors are dispersed in a conducting polymer dispersion (for instance, a PEDOT:PSS ink), and then polymerized or reticulated. A conductive PEDOT:PSS/silk fibroin/tannic acid adhesive hydrogel that could serve as a skin/electrode interface was developed, where the PEDOT:PSS ink was mixed with silk fibroin before polymer cross-linking using Ca^2+^ and tannic acid (Luo et al., 2020). Interestingly, Ca^2+^ can form complexes with PSS, establishing strong interactions between the fibroin and PEDOT:PSS networks. This results in highly stretchable (≈300–400%, up to 32,000% for specific formulations) and adhesive materials, with a conductivity of 3 S/cm.

PSS can also be replaced by another biopolymer-based resorbable dopant, whose cross-linking can lead to the hydrogel scaffold, avoiding dilution of the PEDOT conducting moiety into the material. Our group developed a specific biodegradable hyaluronic-based dopant of PEDOT in order to achieve a PEDOT:HA ink with good conductivity (1.6 ± 0.2 S/cm) in comparison to similar PEDOT:biomolecule inks described in the literature (below 0.1 S/cm) (Leprince et al., 2023a). The introduction of both sulfonic acid and aromatic aminophenylboronic groups on the HA polymer backbone to mimic the PSS structure was shown to enhance ink conductivity above the additive effect, by providing, at the same time, charge carrier mobility and intra-/inter-chain charge transport through PEDOT/aminophenylboronic π-stacking interactions (Figure 5). The HA dopant was further functionalized to introduce alkene moieties that could photo-crosslink with a PEG-bis(thiol) linker to obtain a resorbable PEDOT:HA conductive ink. This ink was inkjetted on a flexible PLGA substrate in order to design a bioelectronic system that could resorb within 2 months in physiological conditions (Leprince et al., 2023b).

IPN conductive hydrogels are characterized by good biocompatibility, easy processing, and high processability (Zhu et al., 2023a). Contrary to a blend of carbon/metallic particles that are island-type distributed into the polymer matrix, the CP chains can come into contact with each other, forming a 3D interconnected conducting network, for electron transporting with relatively low resistance (Yao et al., 2017; Onorato et al., 2019). As a consequence, electronic conductivity in bulk electronically conductive hydrogels not only depends on the intrinsic CP electronic conductivity but also, and more particularly, on the inter-chain connectivity of the CPs and therefore on their spatial arrangement and concentration in the 3D matrix (Li et al., 2017; Xu et al., 2018). Still, limitations of such materials can come from the poor degradability of CPs, unless they are used in their oligomer form. However, the tunability of conductive hydrogel structures and properties make them a real asset in the field of bioelectronics.

The resorbable conductive materials obtained by the blending of scaffolds/substrates and electrical conductors are generally used without significant modification in tissue engineering applications. For these applications, they are applied onto the wound/skin, or implanted into the tissue through surgical intervention or injection. However, resorbable conductive materials can also be shaped into fibers. Another possibility is to combine them with specific patterns of the electrical conductor onto the resorbable 2D substrate to obtain wearable or implanted bioelectronic systems.

### 3.2 Fabricating composite resorbable conductive micro- and nanofibers

Two main strategies have been described to obtain nano- or microfibers of resorbable conductive material (Wei et al., 2023).

The first strategy uses the blending of the polymeric scaffold, selected for its spinning ability (e.g., PLGA, PLLA, PCL, silk, HA, etc.), and the electrical conductor (e.g., CNTs, graphene derivatives, PEDOT:PSS, PANI, PPy, Au nanoparticles, etc.). The blend can then be processed using microfluidics, extrusion printing, wet spinning, or electrospinning to obtain conductive nano- or microfibers, whose diameter (from a few hundreds of nanometers to a few hundreds of micrometers) highly depends on the production method and processing parameters. For instance, Wang et al. (2017) blended PLA and PANI before electrospinning to yield nanofibrous sheets, suitable for cardiomyocyte culture. The thin material sheets could also be rolled up or folded to obtain different shapes. While microfluidics can be used to design core/shell structured fibers, it is worth noting that such a strategy generally results in fibers that consist of the homogeneous blend of the electrical conductor and polymeric scaffold.

On the contrary, the second strategy consists of first fabricating polymer nano- or microfibers using similar techniques as those quoted before (microfluidics, extrusion printing, wet spinning, and electrospinning, with the two last being the more generally used) and subsequently coating them with the conductive material. This strategy results in polymer-based fibers coated with an electrical conductor layer and is largely used for biomedical applications. It should, however, be carefully handled to avoid the issues of delamination or poor adhesion of the conductive layer onto the fiber substrate and is particularly critical whenever the fibers are foreseen to withstand important elongation (Wei et al., 2023). To overcome these issues, the fibers can be coated with the electrical conductor under stretching, or their surface can be nano- or microstructured or functionalized with chemical groups like silanes or dopamine to promote the adhesion of the electrical conductor layer. For instance, electrospun PLLA fibers were coated with carboxylic- and dopamine-modified MWCNTs, rGO, or a mixture of both (Ramasamy et al., 2022). Poly(dopamine) improved not only the adhesion of the conductive materials but also the hydrophilicity and biocompatibility of the fiber-based scaffolds. Zou et al. (2016) prepared an aligned conductive fibrous scaffold of electrospun PLLA fibers that were coated by a PPy layer co-doped with poly(glutamic acid)/dodecyl benzenesulfonic acid by chemical oxidation. Weak polar van der Waals forces between PPy coating and PLLA fibers ensured the coating's mechanical adhesion. Interestingly, Jiang et al. (2019) used the opposite configuration, with the sheathing of PEDOT:PSS wires by silk fibroin in order to design resorbable (within a few weeks), insulated and flexible, connectors for fully organic bioelectronic systems dedicated to ascorbic acid sensing. “Pure” PPy or PEDOT nanotubes (70–175 nm diameter) could also be obtained by the electrochemical deposition of the conducting polymers onto the surface of PLLA/PLGA sacrificial electrospun fibers deposited on the electrode surface of a microelectrode array (Abidian et al., 2008; Abidian et al., 2009; Abidian et al., 2010). Eventually, the fibers could be aligned if the PLLA/PLGA fibers were so templated (Khorrami et al., 2018). The obtained microelectrode arrays were used for chronic neural recording. The PEDOT nanotubes were also filled with dexamethasone, an anti-inflammatory drug, and released the drug upon electrical stimulation (Abidian et al., 2006). These nanotubes that displayed a large electroactive surface were also implemented as mechanical actuators driven by the oxido-reductive incorporation of ions and water in their structures (Eslamian et al., 2021).

Electrospinning is largely employed to fabricate nano- or microfibers dedicated to medical applications. Interestingly, the electrospun fibers can be collected as fiber mats with a fiber anisotropic distribution, typically to design dressings or wearable bioelectronic systems, or alternatively as aligned fibers, particularly interesting for the oriented growth of neural cells or cardiomyocytes. For instance, Ghosh et al. (2020) electrospun a blend of PCL, collagen, and MWCNTs on a rotating mandrel (Figure 5). The alignment of the conductive fibers could be tuned according to the applied voltage and mandrel rotating speed. Random or parallel fibers could be obtained to study the effect of fiber alignment on nerve regeneration. Interestingly, the MWCNTs showed an end-to-end connection insuring electrical conductivity and a coaxial alignment within the PCL-collagen fiber. Zhao et al. (2022a) designed rGO-functionalized electrospun silk patches for post-infarction repair and observed that the anisotropic arrangement of fibers was beneficial to tissue repair in comparison to a random arrangement or the absence of fibers.

If conductive fibers are mainly used in tissue engineering applications, wearable or implanted bioelectronic systems result from the 2D patterning of conductive materials.

### 3.3 Patterning conductive materials onto 2D substrates

The classic microfabrication processes used in “hard” and “soft” microelectronics (i.e., lithography, etching, printing, etc.) can be used to produce resorbable bioelectronic systems. The major advantages of these microfabrication technologies are their well-proven and established processes, the collective production of a large number of devices, and the possibility to define micrometric and even nanometric scale patterns or roughness.

#### 3.3.1 Usual microelectronic system fabrication techniques

Classic microfabrication techniques are used for working with a “hard” (typically silicon, silicon oxide) or “soft” (polymer or bioplastic sheet) substrate. During the fabrication process, the soft substrate is most of the time attached to a more rigid one, like glass or silicon. To pattern metals or semiconductor tracks/electrodes and insulation layers, the traditional approach involves a series of sequential steps, such as deposition (evaporation or sputtering), and the application of photoresists through methods like spray coating or spin coating to delineate the areas to be etched or protected. Subsequently, a selective etching process is employed, which can be either wet (involving acids or bases) or dry, using free radicals from various gases (SF_6_, CF_4_, etc.). These potentially harsh techniques must be adapted to align with the materials used in the construction of resorbable devices while also meeting the peculiar requirements of bioelectronics, such as device flexibility and stretchability.

Kurland et al. (2013) have developed a “silk protein lithography” technique, for which fibroin and sericin proteins are modified by methacrylate groups to obtain photosensitive resists amenable to lithography patterning. The authors blended PEDOT:PSS dispersion with photosensitive sericin in order to obtain a printable PEDOT-based conductive ink. This ink was then spin-coated onto a 50-µm-thick UV-cross-linked fibroin-methacrylate substrate. Subsequently, the coated substrate was exposed to UV light through a mask and developed in water, resulting in devices featuring water-insoluble conductive patterns made of PEDOT:PSS/sericin on the fibroin substrates with a resolution as low as 1 µm (Pal et al., 2016). The conductive tracks could withstand bending without alteration of their resistivity or charge storage capacity.

To step toward device flexibility necessary for bioelectronics, the first strategy consists of building the functional stack on a sacrificial layer, resulting in a thin chip upon transfer or etching. When applied in the context of resorbable bioelectronics, it is necessary to reduce the thickness of resorbable semiconductive and metallic layers to the greatest possible extent and to subsequently transfer or encapsulate them within thin, flexible, and elastomeric resorbable substrates. For instance, Choi et al. (2021) designed a bioresorbable cardiac pacemaker where a tungsten-coated magnesium (W/Mg) (700 nm thick/50 µm) coil antenna and a radiofrequency PIN diode based on a very thin doped silicon nano-membrane (320 nm) were encapsulated into a 50-µm-thick PLGA substrate. Another critical bioelectronic system specification concerns stretchability. If there exists a large choice of materials to design resorbable stretchable substrates such as resorbable synthetic and natural polymers, as described above, this requirement is much more difficult to meet for conductive tracks. To better address the need for stretchability, i.e., elastic limit in elongation, a common approach is to employ zig-zag or serpentine patterns for conductive tracks, where the resulting increase in track length is compensated by an increase in the track cross-sectional area to mitigate the impedance impact. For instance, Held et al. (2022) designed serpentine tracks on resorbable PGS films and compared the device properties with its non-resorbable Ecoflex (silicone) counterpart. The PGS film patterned with serpentine tracks could withstand 130%–350% elongation at the break without any decrease of track conductivity, similar to the non-resorbable bioelectronic system, and be applied for the design of biodegradable (approximately 60% degradation in 3 months) wearable or implanted strain sensors or electronic circuit boards.

Resorbable carbon conductive fillers can also be introduced in bioelectronic systems through the use of microelectronic techniques. Carbon-based structures such as boron-doped diamond (Hébert et al., 2014) or carbon nanotubes (Keefer et al., 2008) are good candidates to boost the effective contact surface of electrodes. Their surface chemistry can also be tuned to improve cell or protein adhesion. Technically, the growth of an oriented or wild forest of carbon nanotubes directly on the electrode array structure requires substrates compatible with high-temperature processes (in the range of 600°C) such as ceramic, quartz, or silicon as performed by Sauter-Starace et al. (2011) on silicon shanks. To circumvent the substrate temperature limit before degradation, carbon nanotubes may be grown at a very high temperature on a mineral substrate and then transferred onto the polymeric flexible film of interest (Roy et al., 2017). The use of graphene-based materials is interesting to obtain transparent electrodes for optical stimulation and characterization. For example, Williams et al. developed, for simultaneous electrophysiology and optical imaging, arrays of transparent neuronal microelectrodes from ultraviolet to infrared wavelengths and displaying a high signal-to-noise ratio (Park et al., 2014a). To achieve this goal, the authors grew graphene flakes on copper blocks and transferred them onto a flexible substrate using a PMMA sheet as the carrier; the copper blocks were then etched using ferric chloride solution, and any remaining residues were removed with a 1:10 HF solution. Afterward, the PMMA sheet carrier was dissolved in acetone, and the graphene layer was patterned to establish a junction with the metal pads and the edge of the transparent part of the design.

Laser sintering processes have been developed to pattern resorbable metals on elastomeric or bioplastic substrates. A layer of metal microparticles or a metallic foil was initially deposited on the substrate. Subsequently, a laser beam was applied to the targeted patterns, causing the metal to melt and penetrate into the first layer of the film. Rahimi et al. (2018) achieved direct integration of Zn conductive patterns onto a stretchable, resorbable, photo-cross-linked acrylamide-based elastomer. In another study, a Zn foil was deposited on a ≈250-µm-thick polymer film subjected to laser cutting. The laser ablation process enabled the metal to penetrate the substrate to a depth of approximately 50 µm. After encapsulation by a second polymer film, the device was used for wireless thermotherapy on the skin. Feng et al. (2019) demonstrated the feasibility of this approach using Mo, Zn, and Fe, creating a set of different transient wearable bioelectronic systems featuring conductive patterns deposited on degradable substrates (composed of cellulose acetate, CMC, PVA, PVP, and polyvinyl acetate) for optical image display and temperature sensing (Figure 5).

Microelectronic patterns are intrinsically made of a stacking of layers on a 2D substrate. However, recent strategies have led to the transition from 2D to 3D bioelectronic systems. For instance, Dadras-Toussi et al. (2022) developed a 3D multiphoton lithography method to obtain 3D patterns on a polymer substrate using a photosensitive resin based on poly(ethylene glycol diacrylate), PEDOT:PSS, and 3-(trimethoxysilyl)propyl methacrylate as the photoinitiator (Figure 5). Liu et al. introduced the concept of three-dimensional macroporous nano-electronic networks, which they called “syringeable electronics” (Liu et al., 2013; Liu et al., 2015). The concept relied on a 2D mesh network of passivated polymer fibers, some of which were coated by conductive nanowires and nanotubes to define electrical connects and contacts of the device. The thinness of the conductive layer and the flexibility of the mesh made it possible to flex it in a 3D conformation and deform it into a syringe restriction for tissue injection. If system resorbability was not targeted (and hence studied) in these publications, the concept could be applied in the future to the minimally invasive injection into the tissues of resorbable medical devices such as sensors. Rogers et al. developed an alternative strategy to obtain 3D electronic systems from 2D layer stacks (Park et al., 2021). The 3D electrode frameworks were created by encapsulating star-shaped patterned chromium/gold (10/200 nm) thin electrodes into a flexible polyimide substrate of the same shape, which was finally attached to a 30% elongated PDMS elastomeric substrate only at specific locations. When the PDMS substrate relaxed, the encapsulated metal electrodes flexed to create a 3D framework, suitable for the encapsulation of a cortical spheroid (Park et al., 2021). Such 3D advanced strategies are still under development and not yet implemented with resorbable metals to the extent of our knowledge. However, they have already paved the way for innovative designs of transient bioelectronics.

#### 3.3.2 Flexible electronics techniques

Another family of microfabrication processes is that used in the field of flexible electronics. These include spin coating, dip coating, coating through metering rods, spray coating, screen printing, inkjet printing, doctor-blading, and stamping, among others. For instance, PEDOT:PSS conductive materials can be processed by these different techniques by tuning the conductive formulation to display different physicochemical properties (surface tension, viscosity, wettability) playing on the pH, concentration, and ionic strength, and the addition of surfactants, viscosity enhancers, or co-solvents, in the dispersion (Glasser et al., 2019). Poorly viscous dispersions (referred to as “inks”) will preferentially be inkjet-printed, while very viscous ones (referred to as “pastes”) will rather be screen printed. Park et al. (2014b, 2017) developed a stamping method to create conducting polymer patterns on the surface of a gold substrate. Agarose hydrogel stamps with desired patterned posts were fabricated from PDMS mold templates. The stamps were then impregnated for approximately 20 min within a CP monomer (EDOT and pyrrole) and dopant (PSS) solution. The impregnated stamps were then applied onto a gold-coated surface and a voltage was applied between the gold electrode and the hydrogel to polymerize the CP film at the post location. The same stamp could be used several times. The thickness of the CP film was controlled by the application time of the stamp and the applied voltage. Interestingly, each post could be further functionalized by specific biomolecules (avidin and laminin peptide sequence) to create specific cell biorecognition patterns. Extrusion printing is also a very popular technique, mostly concerning hydrogel-based formulations. For tissue engineering applications in particular, the development of bioinks combining hydrogels (conductive or not) with cells has appeared as a very active research field during the last decade. In this case, the printability of the material is the key to obtaining printed structures with satisfactory pattern resolution while preserving cell viability (Gillispie et al., 2020). The use of self-healable and dynamic hydrogels is particularly interesting to achieve this goal (Morgan et al., 2022).

Conductive inks and pastes can be developed with different electrical conductors such as gold nanoparticles, carbon nanotubes, graphene derivatives, or MoS_2_ sheets dispersed in polymer solutions or hydrogels. Deo et al. (2022) developed a conductive and 3D printable ink combining thiolated gelatin and defect-rich 2D MoS_2_ nanomaterials (Figure 5). Scanning electron microscopy (SEM) and energy-dispersive X-ray spectroscopy (EDS) evidenced the porous structure of the gelatin scaffold loaded with a homogeneous dispersion of molybdenum in the matrix. Interestingly, MoS_2_ ensured material conductivity and acted as covalent cross-links for the hydrogel, ensuring its printability and stability. The material displayed sensitivity to strain, and potentially to pH and biological interactions. As such, it is foreseen to be of high interest for wearable electronics, and for the development of conformable implanted sensors that would necessitate minimal intervention, thanks to the injectability of the materials. Kim et al. (2023b) developed a thermoplastic tungsten-based conductive paste (7 10^5^ S cm^−1^) composed of a mixture of beeswax (as resorbable matrix), tungsten nanoparticles (as electrical conductor, 500 nm–10 µm size), and glycofurol (as formulation aid). The paste can be filled at moderate temperature (approximately 70°C) into molds and screen-printed, and remains stable at body and room temperatures; it gets degraded in PBS buffer in approximately 80 days. To design resorbable bioelectronic systems, both the substrate and conductive ink patterns should be resorbable. Wang et al. (2019d) designed wearable devices based uniquely on graphene and silk. They combined calcium-plasticized fibroin with graphene (Ca/fibroin/graphene) to directly write or print conductive patterns onto a Ca^2+/^silk stretchable film. Interestingly, the Ca/fibroin/graphene ink displayed self-healing properties that facilitated printing.

Following deposition of the conductive material (ink or paste) onto the substrate, it is often necessary to post-process the material so that the conductive patterns cannot be subsequently washed out or be rapidly dissolved in biological fluids. This post-treatment should, however, not compromise the material functionality, biocompatibility, or resorbability. Depending on the materials, post-processing can include chemistry, light, or heat treatment. Thermal and UV cross-linking are particularly interesting due to their ease of implementation.

This section has evidenced the wide range of processes that can be used to combine scaffolds/substrates and electrical conductors to obtain resorbable conductive materials with a variety of chemical structures, morphologies, properties, and shapes. The next section is dedicated to the description of the applications of such materials in the biomedical field.

## 4 Applications in the biomedical field

Various applications of resorbable conductive materials are emerging and are foreseen to expand in the near future; they require different specifications and shall comply with different regulations. After a short section dedicated to material biocompatibility requirements, we will review the different domains of applications of resorbable conductive materials.

### 4.1 Biocompatibility and other requirements

Wearable devices require sufficient biocompatibility to avoid skin irritation and intradermal sensitization (biocompatibility standard ISO 10993-Part 10: Tests for skin sensitization), whereas implantable devices and tissue substitutes may require the full list of biocompatibility tests required by ISO 10993-1, depending on the contact duration and their invasiveness, in particular ISO 10993-6 (Part 6: Tests for local effects after implantation). In the case of biodegradable materials, the identification and safety of degradation products are also of paramount importance. To this aim, the investigator shall refer to ISO 10993-13 and ISO 10993-15, dedicated to the degradation of products from polymer-based medical devices and metal alloys, respectively. Then, according to the risk analysis, the investigator shall evaluate the tolerable intake (TI) based on the maximum amount of substances below the threshold of adverse effects [the no-observed-adverse-effect-limit (NOAEL)] and modifying factors according to ISO 10993-17. Other material requirements, such as mechanical and conductive properties, also strongly depend on the targeted application. Tissue engineering and 3D cell culture applications demand high biocompatibility because of the intimate contact between materials and cells and require suitable mechanical properties (for instance, soft but stable dynamic hydrogels), but do not require high conductivity (Xu et al., 2021; Gao et al., 2022). On the contrary, a highly conductive material like metal should be used to fabricate the electrical tracks of a resorbable bioelectronic system.

### 4.2 Medical devices for sensing and recording

#### 4.2.1 Wearable bioelectronic systems

In these applications, resorbable conductive materials are essentially obtained by 2D patterning processes on a flexible, sometimes stretchable, substrate (typically an elastomer or polyester film). Wearable bioelectronic systems are mainly dedicated to sensing, display, and stimulation. Electrical stimulation for wound healing will be specifically addressed in the tissue engineering section since the conductive materials employed can serve both as stimulation electrodes and tissue substitutes.

**FIGURE 6:**
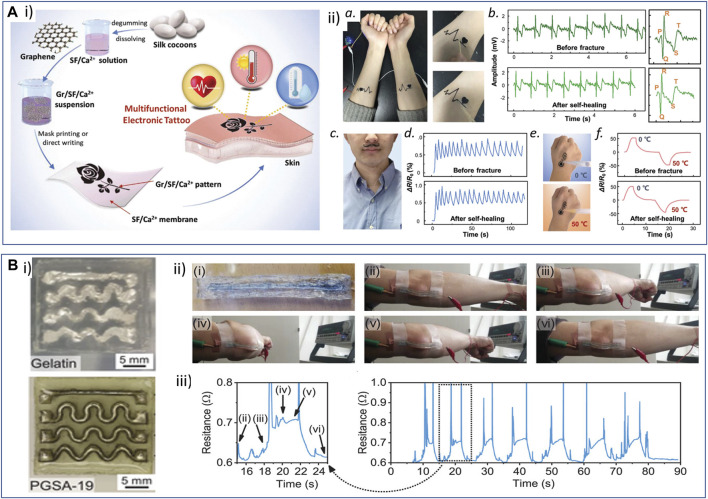
Examples of resorbable wearable sensors. **(A)** *Wearable resorbable tattoo sensors based on silk and graphene materials*: i) silk fibroin (SF) was extracted from *B. mori* cocoons and mixed with Ca^2+^ and graphene aqueous suspension to obtain a conductive ink that could be used to directly write or mask-print conductive patterns onto a SF/Ca^2+^ cross-linked membrane. ii) The resistivity of the obtained devices was sensitive to strain, humidity, and temperature, which makes them useful in designing an electrocardiogram (ECG) recording device (*a,b*), or respiration (*c,d*) and temperature (*e,f*) sensors. Interestingly, the materials presented self-healing properties that made it possible to continue signal recording with conductive pattern fracture and healing. Reproduced with permission, Copyright 2019, John Wiley and Sons, (Wang Q. et al., 2019). **(B)** *Strain sensors based on Galistan metal liquid tracks embedded in gelatin or PGS acrylate (PGSA) films*: (i) the gelatin-based sensor was then applied on a volunteer subject to monitor elbow bending. (ii) The device withstood important bending without rupture. (iii) The motion could be monitored by recording the resistivity of the conductive track. Reproduced with permission, Copyright 2022, John Wiley and Sons (Held et al., 2022).

Most of the described resorbable wearable systems concern physical sensing (strain, temperature) and display. For instance, Wang et al. (2019d) designed a Ca/fibroin/graphene ink whose resistivity was sensitive to strain, temperature, and humidity and that could be printed onto Ca^2+/^silk stretchable films to design wearable devices. The bioelectronic systems were applied onto the skin as sensing tattoos (Figure 6A). A hydrogel dynamic network composed of PVA, borate, and GO particles partially reduced by dopamine oxidative self-polymerization was applied on the skin as a strain-sensing material (Wang et al., 2019c). Held et al. (2022) designed Galinstan serpentine tracks (approximately 2 mm thick) entrapped between two layers of resorbable PGS/PGS-acrylate or gelatin films. Galinstan is a liquid metal, an eutectic mixture of 68% Ga, 22% In, and 10% Sn (mass%), whose biocompatibility has been assessed (Foremny et al., 2021). The high elongation at a break that can withstand the films was used to design strain sensors to monitor elbow bending (Figure 6B). Feng et al. (2019) developed resorbable, temperature-sensitive, and optically transparent Zn electrodes patterned onto PVA films for temperature sensing or wearable heaters. Using the same materials but different patterns obtained by laser sintering, they designed a partially resorbable device for near-field communication and display.

Electrochemical-based sensors hold great potential for sensing and biosensing, and if processed as resorbable, can be used as transient devices deposited onto the skin or injected into the dermis. Liang et al. (2014) obtained a 70-µm-thick graphene/silk conductive composite film using the vacuum filtration of a graphene oxide and silk fibers suspension, followed by the chemical reduction of GO. Platinum nanospheres were then grown onto the film by cyclic voltammetry electrodeposition in order to obtain an H_2_O_2_ sensor or a glucose biosensor after immobilization of glucose oxidase (GOx) on Pt nanoparticles. Though no resorbability study was reported, the employed sensing materials could be resorbable and serve to design a transient wearable bioelectronic system. Kurland et al. (2013) developed a PEDOT:PSS/sericin ink loaded with glucose oxidase enzyme that they printed onto a UV-cross-linked fibroin-methacrylate substrate in order to obtain highly sensitive and selective glucose electrochemical sensors that could withstand 30° bending and be resorbed in approximately 4 weeks. Devices comprising PEDOT- or PANI-based inks loaded with enzymes and printed on different substrates for the analysis of a few metabolites (glucose, lactate, and cholesterol) extracted from sweat (Bilbao et al., 2023) hold great promises. The use of micro- or nanostructured patterns (nanotubes and microcups) of conducting polymers combined with GOx or laminin cell adhesion peptide could further enhance the sensitivity of the sensors in comparison to plain films by increasing the sensing surface (Yang et al., 2014). 3D-printed patterns of a conductive resin could further enhance the recognition sensitivity of the conductive hydrogel. Dadras-Toussi et al. (2022) developed a photosensitive resin comprising poly(ethylene glycol diacrylate), PEDOT:PSS, a photosensitizer, and GOx or laminin cell adhesion peptide (Figure 5). 3D multiphoton lithography was used to obtain self-standing 3D patterns of cell-adhesion conductive materials or GOx-coated patterned microelectrodes that exhibited high sensitivity and specificity. These electrically conductive resins therefore appeared appealing for the design of biosensors and sensing in organ-on-chip devices.

However, the development of resorbable sensors is still limited by several barriers that are yet to be overcome. A main limitation is the poor conductivity of some materials, especially conducting polymers when combined with resorbable hydrogels. CPs’ conductivity can potentially be improved by combining them with other conductive fillers such as carbon nanotubes or graphene derivatives although it could stiffen the materials and decrease their flexibility and stretchability. When designing ion-selective electrodes, another important limitation is the present use of non-safe and non-degradable materials in the design of the sensing part (Cánovas et al., 2019). Therefore, the design of fully resorbable electrochemical-based wearable sensors still largely remains an unmet challenge.

Although a few examples of resorbable wearable sensors have been described in the literature, the fact that the devices are laid onto the skin and can be easily removed after use limits their applications. However, the development of wearable resorbable devices can appear as the first step and proof-of-concept toward the development of resorbable implanted bioelectronic systems and presents high environmental interest in reducing electronics waste (Feng et al., 2019).

#### 4.2.2 Implanted microelectrode arrays and bioelectronic systems

The field of cardiac or neural tissue recording or stimulation is a good example to describe the evolution of materials and processes used through the last 25 years, with the objective to progress toward softer, more conformable, and flexible bioelectronic systems intended for use in the heart, brain, spinal cord, peripheral nerve, muscle, and skin (Tringides et al., 2021). Ultimately, it has now turned its attention toward device resorbability, particularly for specific applications such as short- to medium-term monitoring for epilepsy seizure (Yu et al., 2016; Xu et al., 2019a), neural stimulation for temporary pain relief (Lee et al., 2022), or intracranial pressure monitoring (Shin et al., 2019).

The first electrode arrays cleared by the FDA were the renowned Utah array (Rousche et al., 1998). These arrays comprised a hundred silicon cones, each covered by an insulator (silicon dioxide and/or parylene), except for their apex. These devices sparked considerable interest as they offered the possibility of concurrent neural recordings from up to a hundred channels, thus initiating the development of implantable devices for brain–computer interfaces (Hochberg et al., 2006). However, the risks of damaging blood vessels and triggering microhemorrhages, along with inflammatory response and the eventual formation of glial scar, remain very high (Kozai et al., 2015). In response to this challenge, Neuralink has developed a specialized sewing machine capable of gently introducing flexible polyimide shanks into the cortex while avoiding blood vessels, with the assistance of medical imaging. Owing to the substantial difference between Young’s modulus of polyimide and the stiffness of the brain tissue, the thickness of the shank was decreased to attain a targeted range of 1 µm (Lycke et al., 2023). However, for insertion into the brain tissue, as the thinned structure was no longer stiff enough to prevent buckling, the authors utilized a 75-μm tungsten wire affixed to the shank via a biodissolvable PEG adhesive.

**FIGURE 7:**
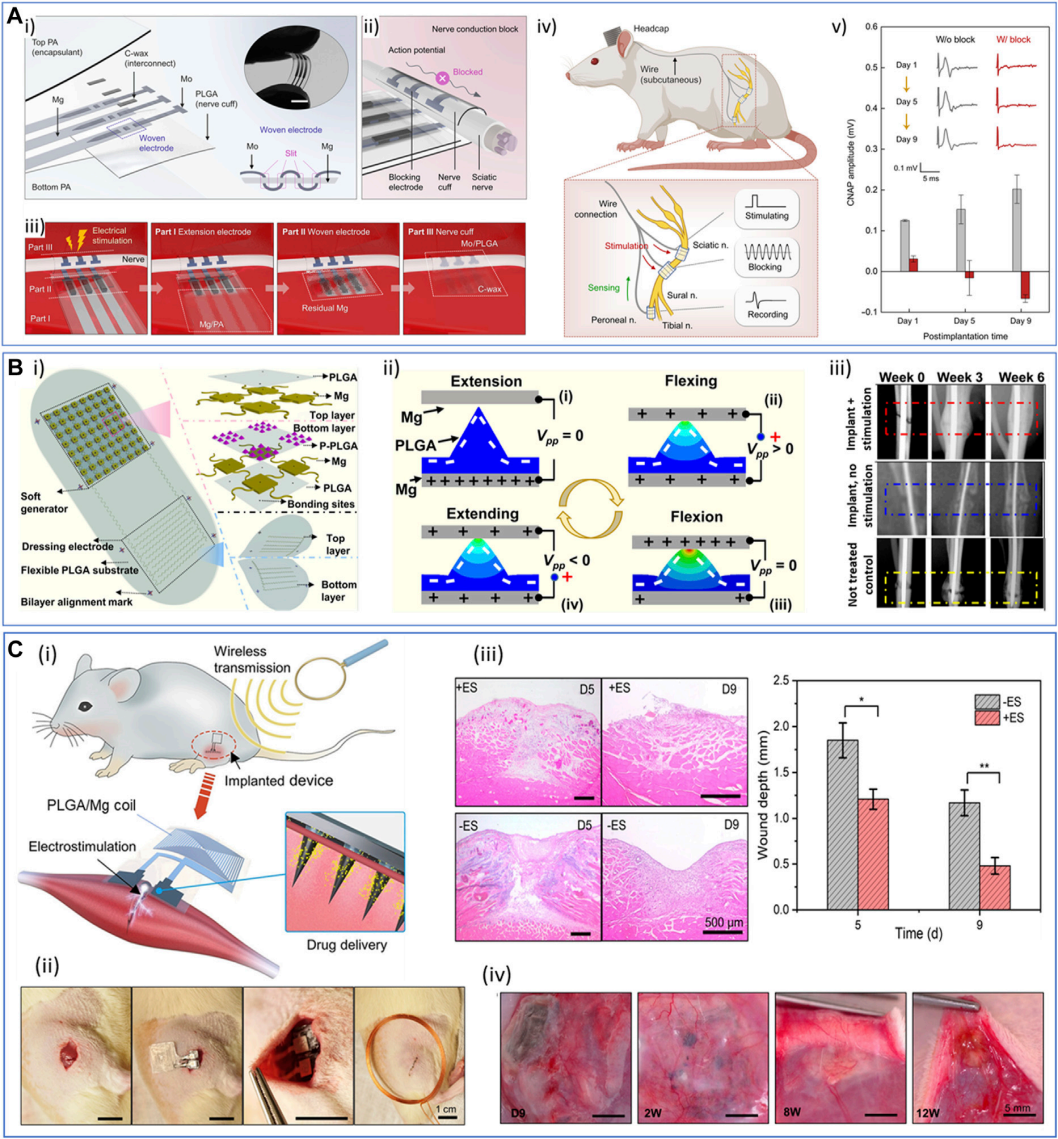
Examples of resorbable implanted medical devices. **(A)** *Bioresorbable nerve cuff as a stimulator to electronically block pain.* i) Schematic representation of the device, showing the Mg/Mo woven electrode structure connected through C-wax. ii) Schematic of the process of the nerve conduction block in the cuff-geometry device. iii) Illustration of the functioning and progressive bioresorption of the device through various stages of its lifetime. iv) Illustration of the *in vivo* implantation: a subcutaneous pathway for the placement of nerve cuffs and wire interconnects (top) and cuffs wrapping around nerves for stimulation, blockage, and recording (bottom). v) Representative compound nerve action potential (CNAP) measurements at days 1, 5, and 9 after implantation, without (w/o) or with electrical stimulation of the pain-blocking device. Copyright 2022, the Authors, published by *Science Advances* (Lee et al., 2022). **(B)** *Self-powered implant to stimulate and monitor the resorption of bone fracture.* i) Representative scheme of the device, showing the two parts: the self-powering generator (top) and the interdigitated electrode dressing for electrical stimulation (bottom). ii) Triboelectric working principle of the self-powering generator part. iii) X-ray photographs of bone fracture healing (right tibia) with time in rodents, when treated with an active device (i.e., implantation of the fully active device, top row), when treated with the inactive device (i.e., implantation of the device for which the self-powering unit has been disconnected from the interdigitated electrode pattern, middle row), when not treated (no device implantation, bottom row). Reproduced with permission, Copyright 2021, the Authors, published by *PNAS* (Yao et al., 2021). **(C)** *Resorbable microneedle-based device for in-depth and wireless electrotherapy and drug delivery into injured muscle tissue.* i) The device is composed of a drug-loaded PLGA microneedle array assembled with a PLGA sheet comprising magnesium coils acting as an antenna for wireless power transmission. ii) Invasive surgical procedure for the implantation of the device in rat muscle injury models. Scale bar: 1 cm. iii) Muscle healing assessment with (+ES) or without (−ES) electrostimulation at days 5 (D5) and 9 (D9) after treatment: representative H&E staining section images (scale bar: 500 µm) and statistical analysis of muscle injury depth. iv) Photographs of microneedle-based devices implanted in rats at different times after therapy (9 days, 2 weeks, 8 weeks, and 12 weeks). Scale bar: 5 mm. Adapted with permission from Huang et al. (2022), Copyright 2022, the American Chemical Society.

Alternately, to reduce the mechanical mismatch with the brain tissue, using softer materials that closely resemble it, such as hydrogels, is a viable approach. A transient microarray for the recording of electrophysiological signals from the cortex was designed by the group of Rogers (Yu et al., 2016). It involved the utilization of thin silicon tracks transferred onto a PLGA film and coated with a SiO_2_ dielectric layer (Yu et al., 2016). More recently, the same research group introduced a bioresorbable device designed for the transient electrostimulation of the sciatic nerve to act as an electronic pain blocker (Figure 7A) (Lee et al., 2022). In this bioelectronic system, fast-resorbable magnesium was used to design tracks in contact with the external connector (kilo-hertz frequency alternating current pulses sent during treatment), whereas slowly degrading Mo was used to make contact with the nerve. Thin strips of Mo were put in contact with Mg strips using a conductive resorbable carbon wax (C-wax) in a woven electrode pattern. The primary objective of this design was to ensure that the rapid resorption of the 50-µm-thick Mg patterns after the electrostimulation treatment (approximately 10 days in phosphate buffer, 2 months when implanted in rodents) did not impose any constraints on the nerve. The nerves were in contact with 700-nm-thick Moelectrodes that were resorbed over a longer time scale (approximately 2 months in phosphate buffer). Note that different substrate materials were also tested and selected for the fabrication of the system to achieve suitable kinetics of bioresorption. Conducting polymers were shown to improve the bioelectronic interface between metals (gold and platinum) and neural tissues, especially by decreasing the electrical impedance and increasing the charge transfer capacity (Ludwig et al., 2006; Richardson-Burns et al., 2007; Khodagholy et al., 2015). CP nanotubes (70–175 nm diameter) (Abidian et al., 2008; Abidian et al., 2009; Abidian et al., 2010) and microstructures (Antensteiner et al., 2017a; Khorrami et al., 2017) advantageously enhanced the surface roughness of the CP films and improved the cellular response of neurons for chronic stimulation and recording. Our group recently developed a resorbable PEDOT:HA ink that can be inkjet-printed and UV-cross-linked on molybdenum tracks patterned onto a flexible PLGA substrate, to serve as a transient recording device of visual stimuli (Leprince et al., 2023b). The conductivity of the tracks and signal recording was maintained for approximately 4 months of implantation on rat cortex, correlated with the system resorption *in vivo*.

Other microelectrode arrays have been designed for various applications, for instance, interacting with bones. Yao et al. (2021) described a bioresorbable and self-powered implant to stimulate and monitor the resorption of bone fracture. The device was composed of two parts: a self-powering generator with micro-pyramid-shaped PLGA structures between two Mg conductive islands and an interdigitated electrode dressing for bone electrical stimulation and healing monitoring (Figure 7B). It can be foreseen that transient electrochemical sensors (for instance, monitoring pH, lactate, and inflammation) can be of high interest in assessing infection risks during the few months following prosthesis implantation (Dai et al., 2023). A thermoresponsive and injectable self-supporting conductive hydrogel made of collagen and polypyrrole was described to detect glucose by amperometry (Ravichandran et al., 2018). The key issue of connectivity was however not addressed or described, and the demonstration of functionality was made in a piece of meat, evidencing the long pathway to human applications.

Microneedle (MN)-based devices to measure or stimulate in a minimally invasive way through the skin have emerged in the last 10 years (Huang et al., 2022; GhavamiNejad et al., 2023). If the benefit of microneedles essentially relies on their skin-anchoring function, which minimizes potential artifacts due to body movements, they still remain invasive systems for microneedle heights above 80 μm, which makes us consider them as implanted devices. If the anchoring function and improvement of measure quality and reliability can counterbalance the regulatory issue due to the invasiveness of such systems, they should expand rapidly for minimally invasive transdermal sensing in the coming years. Microneedles have so far been made mainly from non-resorbable materials, and intended to be removed at the end of their use. For instance, GhavamiNejad et al. (2023) designed an MN patch for glucose sensing based on a blend of hyaluronic acid, dopamine, PEDOT:PSS, Pt nanoparticles, and glucose oxidase. They associated this MN patch as the working electrode with two other patches made of polycarbonate, coated with a Pt layer (counter-electrode) and Ag/AgCl (reference electrode) for glucose transdermal sensing. In the future, the use of long-term (1–2 months) fully resorbable materials can be a real asset to improve the safety of such devices, as well as their disposal after use. In this perspective, Huang et al. (2022) very recently described a resorbable implantable electronic system composed of a drug-loaded PLGA microneedle array (25 MN of 900 µm height) coated by a tungsten layer (1 µm thick), assembled through a conductive paste (mixture of W nanoparticles and candelilla wax) with a PLGA sheet comprising square-shaped magnesium coils (41 µm thick) acting as the antenna for wireless power transmission (Figure 7C). This device was used for the in-depth electrostimulation conjugated to the delivery of anti-inflammatory drugs (aspirin or ibuprofen) into injured muscle tissue. While the Mg coils resorbed in approximately 8 weeks, the degradation of the PLGA material was slower, within 12 weeks. However, the bioelectronic system was not implanted onto the skin surface but directly onto the muscle tissue using an invasive surgery procedure. The microneedle structuration of the device was therefore used for its tissue-anchoring function but not to reduce the invasiveness of the implantation.

### 4.3 Tissue engineering

Tissue engineering has emerged as a promising therapeutic approach, aiming to replace damaged tissue with a functional one that is generally grown within a biologically functional scaffold. Notably, efforts have been focused on constructing biomaterials-based scaffolds that resemble the extracellular matrix to promote cellular regrowth, differentiation, and tissue regeneration. However, designing scaffolds that achieve optimal cellular responses and integration with the host tissue remains a challenge. Recently, there has been growing interest in incorporating both electroconductive and bioresorbable properties into biomaterials used for tissue engineering. Electroconductive materials can mimic the natural electrical microenvironment of living tissues, facilitating accurate cell responses and tissue development. Meanwhile, bioresorbable materials gradually degrade and are absorbed by the body, reducing the need for invasive procedures and potential complications associated with permanent implants. Therefore, the combination of electroconductive and bioresorbable properties in tissue engineering scaffolds holds promise for tissue regeneration and functional organ restoration. The following section will primarily focus on exploring the latest advancements in electroconductive scaffolds for understanding the fundamental mechanisms underlying *in vitro* cellular monitoring and stimulation in the presence of electroconductive elements. Subsequently, the latest advancements in electroconductive and biodegradable materials in tissue engineering will be treated, with a particular focus on the functional restoration of four of the major electroactive tissues: the heart, nervous system, skin, and bones.

#### 4.3.1 Engineered *in vitro* culture systems for cellular monitoring and stimulation

Nowadays, advanced *in vitro* culture systems have emerged as valuable tools for studying the intricate structure and functionality of human tissues, bridging the gap between *in vitro* and *in vivo* investigations. These systems enable the recreation of physiologically relevant microenvironments, in particular the 3D native extracellular matrix (ECM) that serves as a dynamic scaffold that supports cell adhesion, migration, and differentiation. This capability is crucial to accurately recapitulate complex pathophysiological phenomena and support various applications, such as tissue engineering, regenerative medicine, and drug discovery. In this context, the integration of physiologically relevant electroconductivity into 3D *in vitro* cell culture models adds a new dimension to their capabilities, as several tissues, such as the heart, nerves, skin, and brain, are known to display electrical properties (Xu et al., 2021). By incorporating conductive materials into hydrogel matrices, it becomes possible to develop scaffolds that not only support cell growth but also provide electrical stimulation to mimic the natural electrical cues found in living tissues. This unique combination of physicochemical support and electrical conductivity promotes cell proliferation, differentiation, and tissue regeneration, making electroconductive hydrogels a promising tool for the development of functional, biomimetic tissue constructs (Min et al., 2018).

Electroconductive hydrogels play a significant role in the monitoring of cell growth *in vitro*. These hydrogels offer the unique capability of integrating electrical monitoring within the 3D culture systems. The electrical properties of the culture, such as impedance, capacitance, and electrical resistance, can be measured and monitored, which can provide insights into cellular behaviors such as proliferation, viability, and metabolism (De León et al., 2020). The electrical monitoring of cell growth in electroconductive hydrogels allows for real-time and non-invasive assessment of cell behavior. Changes in electrical signals can reflect alterations in cell morphology, cell adhesion, and cellular responses to environmental cues. These electrical cues can serve as indicators of cell growth, functionality, and response to external stimuli (Khan et al., 2019). In particular, impedance measurements at the cell–material interface offer non-invasive and real-time monitoring of cellular behavior and allow to correlate and quantify cell viability over time. Inal et al. (2017) developed a conducting polymer scaffold, made of macroporous PEDOT:PSS integrated with collagen and dodecylbenzenesulfonic acid (DBSA) for the growth and monitoring of MDCK-II cells in a 3D system via electrochemical impedance spectroscopy. The presence of collagen provides cells with a biologically supportive, biomimetic environment to grow. On the other hand, the intimate contact of cells with the electroactive material allows a highly efficient signal transduction at the electrode interface, providing valuable, quantitative insights into MDCK-II growth and migration when compared to the bare collagen scaffold condition (Inal et al., 2017). Furthermore, the possibility to create seamless interfaces between cells and tissues and electronics is gaining significant interests in the contexts where intimate and long-lasting contact is required for monitoring complex *in vitro* systems. Ferlauto et al. (2018) developed a soft electroconductive interface through the selective electrodeposition of a hybrid material made from alginate and PEDOT:PSS onto platinum microelectrodes within a planar microelectrode array (MEA). The integration resulted in a substantial reduction of both mechanical and electrical mismatch at the tissue/electrode interface. Notably, it significantly reduced the electrical noise during recording when iPSC-derived neurospheres were encapsulated within these hydrogels, in contrast to using bare alginate hydrogels. Additionally, it provided a soft, supportive microenvironment for the long-term growth of organoids.

**FIGURE 8:**
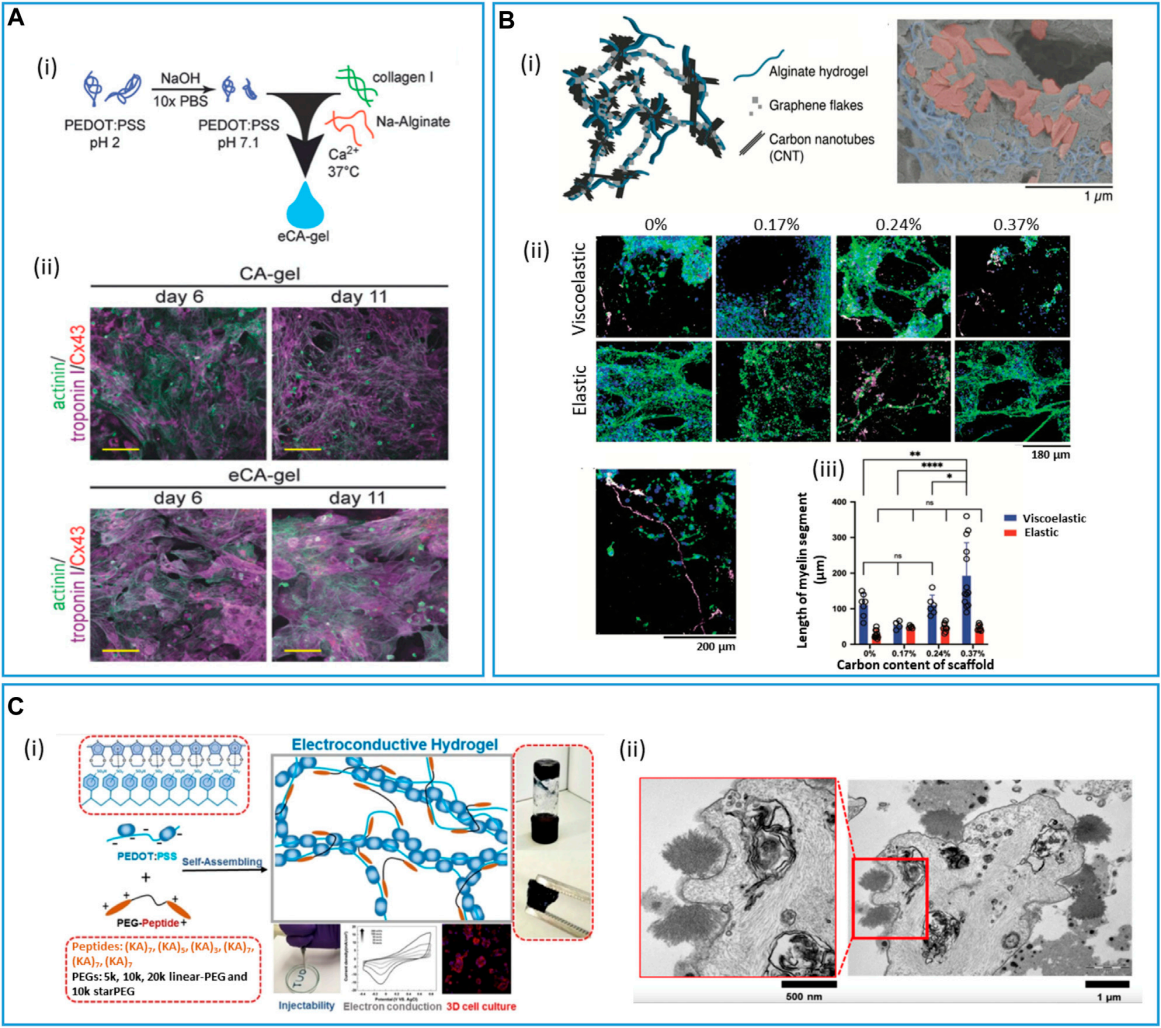
Cell-conductive material interactions in advanced hydrogels and scaffolds for *in vitro* 3D culture models. **(A)** *Biohybrid hydrogel composed of collagen, alginate, and PEDOT:PSS for in vitro hiPSCs-derived cardiomyocyte maturation.* (i) Schematic of the conductive biohybrid hydrogel formation (eCA-gel). (ii) Maturation of hiPSC-derived cardiomyocytes in the non-conductive hydrogel control (CA-gel) and electroconductive hydrogels (eCA-gel). Confocal images projection of tissue constructs stained for cardiomyocyte-specific markers troponin I, sarcomeric-α-actin, and connexin 43. Scale bars: 25 µm. Adapted with permission, Copyright 2018, John Wiley and Sons (Roshanbinfar et al., 2018). **(B)** *Porous, conductive scaffold made of alginate and carbon nanomaterials (CNTs and carbon flakes) for the electromechanical differentiation of neural progenitor cells (NPCs)*. (i) Schematic of the porous scaffold and scanning electron microscopy (SEM) images of the internal structure, showing the entrapment of carbon flakes (red) and CNT (blue) into alginate (gray). Scale bar: 1 µm. (ii) 3D reconstruction of NPC micrographs in scaffolds of different mechanical properties (viscoelastic and elastic) and carbon content (%) after 6 weeks in culture. Staining for oligodendrocyte markers TuJ1 (green), myelin basic protein (MBP, magenta), and NPC (red). Scale bars: 180 µm. (iii) Quantification of the length of myelin for different carbon contents (%) and mechanical properties (viscoelastic, elastic) of the scaffolds. Adapted with permission, Copyright 2022, John Wiley and Sons (Tringides et al., 2023). **(C)** *Characteristics of PEDOT:PSS/peptide-PEG conductive dynamic hydrogels.* (i) Self-assembling and representative hydrogel formation through reversible and non-covalent interactions. (ii) Transmission electron microscopy (TEM) images of MSCs encapsulated in the PEDOT:PSS/peptide-PEG hydrogel showing the formation of nanofiber bundles around cells. Scale bars: 500 nm (left) and 1 µm right). Adapted with permission from Xu et al. (2018), Copyright 2018, the American Chemical Society.

In addition to cell and tissue monitoring, the possibility to electrically stimulate cells *in vitro* (via endogenous or exogenous routes) has been shown to deeply influence cell behavior and stem cell differentiation, offering a potential strategy for generating tissue-specific cell types in regenerative medicine and tissue engineering applications (Chen et al., 2019). In a seminal study, a biohybrid hydrogel composed of collagen, alginate, and PEDOT:PSS was shown to induce *in vitro* maturation and beating properties of hiPSCs-derived cardiomyocytes, upon external electrical stimulations (Figure 8A) (Roshanbinfar et al., 2018). Similarly, the *in vitro* electrical stimulation of neural stem cells (NSCs) embedded in a collagen-based network containing PPy showed NSC proliferation and differentiation into astrocytes, provoking NSC transcriptome alternation, such as genes involved in cell proliferation and synaptic remodeling (Xu et al., 2022).

The molecular mechanisms underlying electrically induce cell growth and differentiation involves intricate cellular signaling pathways and gene expression regulation (Thrivikraman et al., 2018; Chen et al., 2019; Katoh, 2022). Electrical stimulation triggers the activation of specific ion channels and transporters on the cell membrane, leading to changes in intracellular ion concentrations, particularly calcium ions. These changes initiate downstream signaling cascades, such as activation of protein kinases, phosphorylation of transcription factors, and modulation of gene expression. This ultimately regulates cell growth and differentiation by influencing cell cycle progression, proliferation, and the activation of lineage-specific genes. Additionally, electrical stimulation can modulate the activity of growth factors and cytokines, further contributing to cell growth and differentiation processes. It is however worth noting that the specific molecular mechanisms underlying electrically induced cell growth and differentiation are still partially unclear and can greatly vary depending on the cell type and on the specific characteristics of the conductive material in contact with cells.

It is also evident that cellular differentiation in such systems relies on the combination of mechanical and electrical stimulations through a concert of biochemical and mechanobiological pathways (Bielfeldt et al., 2022). The possibility to independently investigate the two stimuli might give further insight into the biological processes underpinning cell type formation and help develop a more accurate platform for regenerative medicine applications. In a recent study, Tringides et al. (2023) developed a porous conductive scaffold by incorporating carbon nanomaterials (CNTs and carbon flakes) into an alginate hydrogel matrix with tunable mechanical and electrical properties in the range of the typical neural tissue values (Figure 8B). By varying the degree of matrix viscoelasticity and CNT content, the authors could study and characterize the ability of encapsulated neural progenitor cells to grow and differentiate under different electrical and mechanical environments. Notably, it was found that the more viscoelastic and conductive scaffolds produced denser neurite networks and differentiated into astrocytes and myelinating oligodendrocytes. These findings were consistent with previous studies examining the influence of matrix viscoelasticity and conductivity associated with carbon nanotubes (CNTs) on the maturation of neural cells (Chaudhuri et al., 2015; Chen et al., 2019; Samanta et al., 2022). Importantly, this study provides novel insights into the individual contributions of each stimulus, shedding light on their independent effects.

Interestingly, the dynamic rearrangement of the internal matrix nanostructure, as a result of the continuous cell–environment interaction, was shown to further alter the endogenous conductivity of the system. This phenomenon was particularly evident upon cell encapsulation in dynamic hydrogel networks. In an electroconductive PEDOT:PSS/peptide-PEG hydrogel, the assembly of peptide-PEG and negatively charged PEDOT:PSS nanostructure resulted in a dynamic non-covalent network, prone to structural rearrangements under cellular action, such as growth and maturation (Figure 8C) (Xu et al., 2018). Despite that the underlying biological and chemical mechanisms remained undetermined, the increase in conductivity after 5 days of mesenchymal stromal cell culture was associated with a simultaneous marked change in the matrix network structure, with the presence of nanofiber bundles around the cells.

In conclusion, the exploration of the underlying biological processes governing cellular differentiation and phenotypic expression in the presence of electroconductive elements is crucial for advancing regenerative medicine applications. Further investigation of these intricate mechanisms offers an opportunity to develop refined and precise platforms that can accurately replicate the complex cellular environments found *in vivo*. By unraveling the intricacies of cell–biomaterial interactions at a fundamental level, new insights can be gained into cellular fate determination, lineage commitment, and the modulation of cellular functions, thereby enhancing *in vitro* modeling capabilities and driving the development of innovative therapeutic approaches for tissue engineering applications.

#### 4.3.2 Cardiac tissue engineering

Cardiovascular diseases, such as myocardial infarction, occur with imparted electrical activity and alteration of the heart's mechanical function, which causes severe damage to the heart tissue, such as the loss of cardiomyocytes (CMs). The infarcted myocardial tissue triggers a pro-fibrotic response, which is responsible for the stiffening of the tissue and loss of contractility. Compared to other tissues, the cardiac regenerative capacity is really limited. In this context, cardiac tissue engineering aims at assembling tissue patches, adhesives, or injectable materials, which can intrinsically integrate with the cardiac tissue. Notably, apart from being biocompatible and biodegradable, those scaffolds should match the mechanical and electrical behavior of the myocardium ECM in order to mimic and transduce the heartbeat. Moreover, as the heart has a low inherent regenerative capacity, it is crucial that these systems allow for the maturation and differentiation of implanted cells into conductive and contractile CMs. The conductivity range of native myocardium, which can be used as reference values for designing electroconductive hydrogels for cardiac repair, varies from 5 × 10^−5^ S cm^−1^ (transversally) to 0.0016 S cm^−1^ (longitudinally) (Gao et al., 2022).

The main strategy used in cardiac tissue engineering consists of the addition of carbon nanotubes, gold nanoparticles, and graphene and its derivatives, or conducting polymers such as PPy, PANI, and PEDOT, to hydrogels. The electrically conductive properties of the materials incorporated in ECM-mimic hydrogels have been shown to improve *in vivo* cardiac function and electrical impulse propagation in the absence of external stimulation (Morsink et al., 2022). On the other side, it is widely accepted that the inclusion of nano- or microstructured materials, regardless of their conductivity, also changes the stiffness and topography of the scaffold, influencing the maturation and irritability of the heart tissue. It has been argued that the incorporation of intrinsic conductive materials results in the formation of tight connections between cell membranes and the scaffold, forming a hybrid conductive network that, in turn, facilitates signal propagation and excitability of heart cells. Furthermore, the presence of nano- or microelectrical conductors has been shown to promote cell attachment and the expression of cardiac-specific markers (e.g., sarcomeric alpha-actin striations and connexin 43 (cx-43) (Morsink et al., 2022). Lee et al. (2019) have characterized the effects of the addition of CNTs, GO, and rGO in gelatin methacrylate (GelMA) hydrogels on cardiomyocytes' function and behavior, underlying the importance of directing cardiac tissue regeneration through mechanical and electrical cues of carbon derivatives. GelMA-CNT and GelMA-rGO scaffolds resembled typical stiffness values of the heart, featured electrophysiological properties, and displayed electrical conductivity. Surprisingly, the different types of carbon functionalization direct different types of *in vitro* tissue maturation. While GelMA-CNT hydrogels led to ventricular-like tissue, GelMA-GO hydrogels guided to atrial-like tissue. This is likely the result of the integrin-mediated differentiation of the CMs, which was stimulated differently by using different carbon nanoparticles and thus different gel topography.

Injectable electroconductive hydrogels can be deployed into the infarct site and surrounding area to promote myocardial tissue function restoration, providing an effective and minimally invasive method. Once injected into the myocardium, the hydrogel undergoes fast *in situ* polymerization, not only acting as a structural support for the damaged tissue but also bridging the electrical mismatch between healthy and damaged CMs, promoting cardiac resynchronization (Gao et al., 2022). Liang et al. (2018) developed a series of electroconductive hydrogels by combining PPy with gelatin and PEGDA via a two-step Michael addition reaction. The injectable material was directly painted into the infarcted area in a myocardial infarction rat model, and *in situ* polymerization occurred in less than 10 s, avoiding any adverse hydrogel leakage and creating a seamless interface between the hydrogel and the tissue. Four weeks post-implantation, the PPy-containing hydrogels were mostly degraded, likely due to enzymatic and hydrolysis degradation, and the cardiac tissue presented a remarkably improved electrophysiological signal restoration and reconstruction. Injectable hydrogels could also load cells, drugs, and biomolecules into the tissues, further boosting myocardial restoration and cardiac function. Bao et al. (2017) developed an injectable PEGDA-melamine/hyaluronan-thiol (HA-SH)/GO hydrogel, where GO interacted with the melamine core through π–π conjugation, and this was loaded with adipose-derived stem cells. The flowable behavior of the hydrogel allowed the safe injection of the cells into the myocardial infarction area in rats, by mitigating the injection-associated shear stress. *In vivo* results have shown a remarkable improvement in heart function, characterized by an increase in ejection fraction and reduction of the infarcted area together with enhanced angiogenesis (Bao et al., 2017).

Among other electrically conductive nanoparticles, AuNPs are widely employed for cardiac tissue engineering due to the ease of producing particles with different sizes, shapes, and surface properties, allowing for tunable electrical and mechanical properties. Saravanan et al. (2018) incorporated graphene oxide gold nanosheets (GO-Au) into degradable chitosan polymer. *In vitro*, the scaffold exhibited Go-Au concentration-dependent degradation properties, supported cell attachment and maturation, displayed no cytotoxicity, and increased electrical conductivity and signal propagation. When tested *in vivo*, in a rat model of myocardial infarction, the cardiac patch showed improved heartbeat, contractility, conductivity, and restoration of the ventricular function. However, despite low levels of inflammation, contrary to the *in vitro* tests, after 5 weeks post-implantation, the patch was still present in the heart, suggesting the need for more elaborative studies on the long-term fate of this class of implanted scaffolds and their long-term effects on cardiac function (Saravanan et al., 2018). In general, to be clinically relevant, cardiac patches should withstand the continuous, dynamic-stress environment of the heart and should hold great tissue adhesion, enabling tissue restoration while providing mechanical and electrical support to the infarction site. As such, conductive dynamically bonded hydrogels, featuring self-healing behaviors, are the desired materials to mimic the ability of native cardiac tissue to regenerate through the continuous formation of new chemical bonds (Rogers et al., 2020). To further enhance hydrogel wet-adhesion to tissues, conductive dopamine-based materials have been widely employed due to their notable gluing properties. To this end, Jing et al. (2017) developed electroconductive chitosan-based hydrogels functionalized with GO and featuring dynamic, self-healing, and self-adhesive behavior, by the incorporation of poly(dopamine). *In vitro* cell culture results showed enhanced viability and proliferation of human stem cell-derived fibroblasts and CMs in the conductive chitosan–dopamine/GO hydrogels, as well as faster, spontaneous, and physiologically relevant beating rate when compared to the non-conductive control.

**FIGURE 9:**
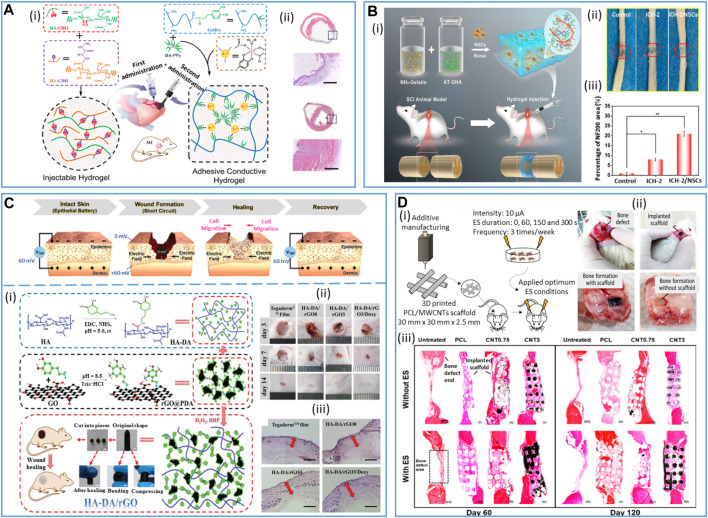
Conductive hydrogels for tissue engineering applications. **(A)** *Co-administration of an injectable hydrogel (HA-CHO/HA-CDH) and an adhesive conductive hydrogel patch (Gel-DA/DA-PPy) to treat myocardial infarction.* (i) Schematic depicting the synthesis and co-administration of the two hydrogels. (ii) Masson's trichrome-stained sections showing infarct size and related fibrotic tissue (blue stained infarct scar) of the myocardium for the untreated group (top) and the hydrogels co-administration group (bottom). Scale bars: 1 mm. Adapted with permission from Wu et al. (2020), Copyright 2020, the American Chemical Society. **(B)** *Injectable, conductive, self-healing hydrogel (ICH) scaffold for spinal cord injury repair.* (i) Schematic illustration of the hydrogel, based on amino-modified gelatin (NH_2_-gelatin) and aniline tetramer-grafted oxidized hyaluronic acid (AT-OHA), loaded with exogenous neural stem cells (NSCs) and its administration in a rat model of total spinal cord injury (SCI). (ii) Total spinal cord resection samples 6 weeks after implantation of ICH, NSCs-loaded ICH (ICH-NSCs), and without any treatment (control group). (iii) Semi-quantitative analysis of the proportion of neurofilament 200 (NF200)-positive regions in rats by immunofluorescence staining. Adapted with permission from Liu H. et al. (2023), Copyright 2023, the American Chemical Society. **(C)** *Conductive hydrogels for skin repair and wound dressing.* (Top) Trans-epithelial potential and electric field at the wound site before and after the healing process. Reproduced with permission, Copyright 2020, John Wiley and Sons (Korupalli et al., 2021). (Bottom) (i) Diagrammatic sketch of soft hemostatic antioxidant conductive HA-DA/rGO@polydopamine (PDA) hydrogel preparation, macroscopic characteristics, and implantation at the wound site. Scale bar: 5 mm. (ii) Pictures of wounds at days 3, 7, and 14 post-implantation. (iii) Granulation tissue (red arrows) thickness for the different groups on day 14 post-implantation. Scale bar: 500 µm. Tegaderm: commercial film dressing (control); HA-DA/rGO0: HA-DA hydrogel in the absence of rGO, HA-DA/rGO3; HA-DA hydrogel cross-linked with rGO@PDA, HA-DA/rGO3/Doxy: doxycycline-loaded HA-DA/rGO@PDA hydrogel. Adapted with permission, Copyright 2019, John Wiley and Sons (Liang et al., 2019). **(D)** *In vivo study of conductive 3D-printed PCL/MWCNTs scaffolds for bone tissue engineering.* (i) Schematic illustration of the experimental setup: synthesis of the conductive scaffolds and their implantation with electrical stimulation (ES) treatment. (ii) Bone defect formation in the animal model and bone tissue formation with and without conductive scaffold after 60 days. (iii) Cross-section histological images of bone tissue formation at the bone defect for all groups (PCL, PCL/MWCNTs 0.75% wt and PCL/MWCNTs 3% wt) after 60 and 120 days, with and without electrical stimulation (ES). Adapted from eSilva et al. (2021), Copyright 2022, Springer Nature.

Interestingly, Wu et al. (2020) developed a combined approach therapy, by synthetizing two types of biodegradable and bio-conductive hydrogels for the co-administration of a self-adhesive conductive hydrogel patch and injectable and self-healable hydrogel to the infarcted myocardium (Figure 9A). The dynamic, injectable hydrogel, obtained via Schiff-base hydrazone bonds between oxidized HA and hydrazide-functionalized HA, was first injected at the infarcted area in order to provide mechanical support and promote angiogenesis. Subsequently, the self-adhesive hydrogel patch, obtained by combining gelatin-dopamine and dopamine-modified PPy upon Fe^3+^ trigger, was painted and rapidly bound to the outermost layer of the beating myocardium in order to provide high hydrogel–tissue integration and homogenous electrical conductivity. These combined hydrogel/patch approaches featured good biodegradability of both materials upon *in vivo* implantation and a more pronounced improvement of the conductive functions, in terms of electrophysiological, histological, and antigenic outcomes when compared to single-mode systems (cardiac patch or injectable hydrogel) (Wu et al., 2020).

#### 4.3.3 Nerve tissue engineering

Nerves' regrowth upon injury can be slow and often results in incomplete functional recovery as a consequence of the limited nervous tissue regenerative capacity. Endogenous and external electrical stimulations have been shown to promote nerve growth, enhance axonal regeneration, and guide the direction of nerve growth. Similar to cardiac tissue, recent studies have shown that conductive hydrogels can be used as electrical stimulators and greatly promote neural cell proliferation, elongation, orientation, and neuronal differentiation, making these scaffolds promising for tissue repair (Rogers et al., 2020; Gao et al., 2022). Notably, conductive hydrogels employed as structural and electrical “functional bridges” at the lesion site could stimulate new neurogenesis and subsequent functional neural network formation. Such hydrogels are also the most suitable candidates for delivering stem cells, retaining and protecting cells after injection at the target tissue, and even directing the differentiation of neural stem cells by fine-tuning the modulus and conductivity of hydrogels. Notably, electrical stimulation in the presence of neural stem cells (NSCs) has been demonstrated to enhance their differentiation toward a neuron-like phenotype, as evidenced by a general increase in TuJ1 expression and the development of longer neurites, promoting neuronal differentiation over astrocyte differentiation (lower GFAP levels of expression) (Zhu et al., 2019; Sordini et al., 2021).

Spinal cord injury is a serious and disabling health issue that causes loss of motor functioning and sensing. It causes neural necrosis and axonal disruption with a low to null regenerative capacity. Implantation of injectable conductive hydrogels can restore the spinal cord by providing physiologically relevant electrical signal pathways (estimated to be 8–100 S cm^−1^ for the spinal cord). The promotion of specific cell proliferation and differentiation at the injured site has been shown to be a promising clinical treatment. Liu et al. (2023) developed injectable, biodegradable, and self-healing hydrogel scaffolds based on aniline tetramer grafted onto oxidized hyaluronic acid and amino-modified gelatin, eventually loaded with NSCs (Figure 9B). The resulting hydrogels displayed electroconductive (134 S cm^−1^) and mechanical (G′ = 446 Pa) properties that matched the natural spinal cord values. When injected into the back skin tissue of Sprague–Dawley rats, it showed good compatibility and complete degradation after 16 days (aniline tetramer is generally metabolized in physiological environments). Furthermore, when injected in a rat model of total spinal cord resection, the loaded NSCs differentiated into neurons and further grew into new nerve axons. The self-healing properties, together with the good conductivity of the hydrogel scaffold promoted endogenous neurogenesis, by providing continuous tissue electro-activity, which finally led to nerve regeneration and locomotor function recovery. Notably, the controlled degradation rate of the hydrogel allowed the complete axon integration within the tissue, without hindering their prior growth and connection (Liu H. et al., 2023). Similarly, Luo et al. (2022) developed an ECM-mimic hydrogel composed of borax-functionalized oxidized chondroitin sulfate, PPy, and gelatin, featuring injectable, self-healing properties (Schiff-base and borate-diol ester dynamic bonds), as well as physiologically relevant electrical (50 S cm^−1^ conductivity) and mechanical (G′ = 930 Pa) properties. The injection of such hydrogels into the injury site to fill the lesion cavity promoted endogenous neural stem cells' neurogenesis and induced myelinated axon regeneration into the lesion site, thereby achieving significant locomotor function restoration in rats with spinal cord injury. Hydrogel degradation occurred within 21 days post-implantation (into rat subcutaneous tissue) with no histological damage to major organs (kidney, heart, liver, and spleen) (Luo et al., 2022). Furthermore, external electrical stimulation was proven to enhance recovery of nerve injuries. Zhang et al. (2021) developed a wireless method for spinal cord regeneration by developing magneto-metric Fe_3_O_4_ Ba TiO_3_ NP loaded with biodegradable ECM-like HA/collagen hydrogels and used an external magnetic field to induce electrical stimulation. Transplantation of this scaffold with wireless stimulation in the rat hemi-section spinal cord injury model showed the promotion of neural regeneration and paved the way for non-invasive remote control electrical devices for soft tissue stimulation. Contrary to the central nervous system, the peripheral nervous system has a good regenerative capacity after injury. Nevertheless, the complete restoration of nerve defects remains a big clinical challenge due to the side effects related to the use of autologous grafts as the golden standard, such as donor shortages, rejection, and infection risks. In the framework of tissue engineering, nerve guidance conduits (NGCs) are promising alternatives to nerve auto-grafting (Vijayavenkataraman, 2020). NGCs are generally tubular polymeric scaffolds that act as functional bridges between the injured nerve endings, providing structural and trophic support for the axon reconstruction along the conduit. As such, the requirements for an ideal NGC include suitable mechanical properties and structural support for promoting the longitudinal alignment of the new axons, high porosity, biocompatibility, and electrical conductivity. In this regard, an NGC made of PEDOT NPs incorporated in a tetrapeptide-modified chitin hydrogel was developed (Huang et al., 2021). The incorporation of PEDOT NPs, together with the highly porous structure of the hydrogel film, significantly promoted sciatic nerve regeneration after 20 weeks, featuring gastrocnemius muscle restoration and thickness of myelinated axon, similar to the auto-graft control groups. Despite the promising results, data on the conductive hydrogel degradation rates were reported only for *in vitro* experiments. Park et al. (2020) developed conductive NGCs by polymerizing *in situ* a GelMA/GO composite hydrogel using an annulus mold and further chemically reducing it to the r(GO/GelMA) form under mild conditions. The obtained material featured suitable mechanical stability (57 kPa Young’s modulus), permeability, flexibility, and electrical conductivity (90 S cm^−1^) for nerve restoration. *In vitro*, the hydrogel at its reduced form r(GO/GelMA) displayed remarkable neurogenesis capacity compared to the non-reduced form and pristine GelMA hydrogel, likely due to the electroactive interaction of rGO within the hydrogel. *In vivo* studies with a 10-mm peripheral defect model showed that the r(GO/GelMA) material significantly promotes neural regrowth, myelination, and functional regeneration of muscle and nerve tissues within 8 weeks post-implantation, without significant toxicity to other major organs. Nevertheless, at week 8, the r(GO/GelMA) hydrogel was only partially degraded at the implantation site (Pyarasani et al., 2019). Despite encouraging *in vitro* data on the degradation kinetics of hydrogel, future long-term investigations on material degradation and its systemic toxicity are necessary.

#### 4.3.4 Wound dressing and skin repair

Similar to the myocardium and nerves, the skin also exhibits sensitivity to electrical signals, characterized by a conductivity ranging from 2.6 to 1 × 10^−7^ S cm^−1^. In intact skin, the epithelial tissue transports ions to the epidermis to form a transdermal potential (∼10–60 mV). When a wound occurs, the homeostatic balance of the transdermal potential is disrupted, resulting in a potential decrease at the wound site and the formation of positive currents flowing toward the wound center. As a result, the formation of these endogenous electric fields promotes cellular recruitment and proliferation at the wound site, following the electrical gradient (electrotaxis), until complete wound healing is achieved, and the initial transdermal potential is restored (Shaner et al., 2023).

Therefore, to enhance such a regenerative capacity of the skin and accelerate the healing process, conductive hydrogel dressings can be employed. Such materials applied at the wound site should possess multifunctional properties. Notably, conductive dressings should exhibit adhesiveness to secure the dressing, possess antibacterial properties to prevent infections, scavenge radicals to minimize oxidative stress, and have sufficient conductivity and good mechanical properties to support cell migration, proliferation, and tissue regeneration. In this context, a series of soft hemostatic antioxidant conductive hydrogels based on HA-graft-dopamine and rGO have been developed (Liang et al., 2019) (Figure 9C). *In vivo*, the injected hydrogels showed high tissue adhesive properties, viscoelasticity, conductivity, and hemostatic ability. The hydrogel dressing showed substantial tissue repair after 7 days of treatment in a mouse full-thickness skin defect repair model, accelerating epidermal remodeling and promoting neovascularization at the lesion site. Furthermore, *in vitro* drug release studies in physiological conditions and zone of inhibition tests of antibiotics-loaded hydrogels showed a promising sustained drug release capacity of these hydrogels (Liang et al., 2019).

Apart from the re-establishment of the skin's endogenous electrical field through conductive dressing, the application of an external electrical field can also improve wound healing. Lei et al. (2021) developed an adaptive conductive hydrogel by incorporating tannic acid and human-like collagen into a polyvinyl alcohol and borax hydrogel dynamic cross-linking network. The dynamicity of the bonds imparted the hydrogel with self-healing and self-adaptive behavior at the wound site, which facilitated cell-to-cell signaling, promoted hemostatic repair, and maintained hydrogel structural and functional capacity. Furthermore, such adaptive behavior allowed endogenous and external current conduction, promoting electrostimulation in wound tissue. The combination of electrostimulation and hydrogel greatly promoted L929 cell migration and proliferation and *in vivo* wound healing, with subcutaneous tissue (blood vessels and pores) reconstruction. *In vivo* degradability tests showed gradual and complete hydrolytic degradation of the hydrogel in accordance with wound repair, avoiding any possible secondary damage due to adhesive peeling (Lei et al., 2021). Interestingly, the incorporation of additional characteristics into these electroconductive hydrogels could impart them with superior regenerative capabilities for a broad range of tissue engineering applications. In this context, Li et al. (2020) developed biomechanically active conductive hydrogels that could promote wound healing through the combination of biomechanical and biochemical functions. By combining quaternized chitosan, poly(dopamine)-coated reduction graphene oxide and poly(N-isopropylacrylamide), a series of multifunctional, injectable hydrogels have been developed. While the biochemical characteristics provided the hydrogels with self-healing, antioxidant, and adhesive properties, as well as good conductivity, the self-contraction ability of the poly(N-isopropylacrylamide) in response to temperature changes played a crucial role in wound closure, leading to a significant improvement in tissue restoration in an *in vivo* full-thickness skin defect (Li et al., 2020).

#### 4.3.5 Bone tissue engineering

Bone has a naturally good regenerative capacity to recover small bone defects; however, larger fractures generally require external intervention to restore the damaged tissue (Schemitsch, 2017). In this context, bone tissue engineering demands the creation of biocompatible, osteogenic scaffolds, which can sustain the dynamic nature and irregular structure of the microenvironment of the bone. These scaffolds should allow bone tissue remodeling and regeneration, providing physicochemical properties for osteoblast attachment, strong mechanical properties, and scaffold mineralization. Bone tissue engineering undergoes a process initiated by the migration and recruitment of bone cells, subsequently followed by proliferation, differentiation, and matrix formation (calcium deposition) (Mostafavi et al., 2020). Electrical stimuli play a key role in a broad spectrum of biological processes involved in bone regeneration, such as angiogenesis, cell division, and signaling. In this context, conductive hydrogels have been shown to effectively stimulate and sustain the effect of endogenous electric fields in bone tissue repair (Arambula-Maldonado et al., 2022).

In order to increase scaffold elastic modulus, roughness, and conductivity, the main strategy consists of the development of functional hybrid hydrogel/fiber composites. Notably, the combination of fibrous PANI in a graphene-containing hydrogel demonstrated that the inclusion of conducting fibers yielded materials that better supported human osteoblast-like cell adhesion, proliferation, and morphology when compared to hydrogel alone (Khorshidi et al., 2018). However, considerations on the *in vivo* biological toxicity of PANI, especially in the form of fiber, must be taken into account. On the other hand, the introduction of GO to porous hydrogel matrices was shown to significantly improve the efficiency of the mineralization process through electrostatic interactions, which sustained crystal growth and apatite deposition. In another study, β-cyclodextrin (β-CD)-functionalized rGO was used as a conductive component and mixed with GelMA/acryloyl-β-cyclodextrin (Ac-CD)-based photo-cross-linked hydrogel (Li et al., 2022). Such a hydrogel was shown to accelerate the *in vivo* defect repair in a rat skull defect model by promoting collagen deposition and mineralization. *In vitro* degradation tests confirmed the ability of the hydrogels to provide adequate mechanical and structural support in the early stages and to subsequently undergo gradual degradation, providing a good foundation for *in vivo* bone repair (Li et al., 2022).

Recently, conductive scaffolds were also obtained through the incorporation of multi-walled carbon nanotubes (MWCNTs) in a biodegradable PCL hydrogel and cut to fit the bone defect in rat skull models (eSilva et al., 2021) (Figure 9D). Upon exogenous electrical stimulation over a period of 120 days, thicker bone tissue reformation was observed, along with angiogenesis and mineralization. In particular, the combination of conductive hydrogel and electrostimulation was shown to play an important role in bone remodeling, inducing osteoclast formation and functioning.

## 5 Challenges and perspectives

As shown in this review, the field of resorbable conductive materials applied to biomedical applications is emerging and already fast expanding, giving rise to a growing interest in the community of advanced medical devices and tissue engineering. This is shown by the large number of publications in the domain reported in this review, and their novelty (>75% in the last 5 years, >95% since 2013). The field of resorbable bioelectronic systems is relatively young compared to chronic implantable devices such as pacemakers, deep brain stimulators, neural stimulators, or cochlear implants. However, there exist already several proofs of concept at the *in vitro* and preclinical (mainly in rodents) stages. The fact that implanted systems may be designed as resorbable is expected to extend their use to non-chronic pathologies requiring transient therapy and/or monitoring. Tissue engineering applications are quite novel in general, and the use of conductive materials will help address important applications (for instance tissue healing and heart and nerve reconstruction) that are still at the proof-of-concept stage. Therefore, it can be foreseen that the requirement for resorbable conductive materials that can optimally interface with tissues or act as tissue substitutes will quickly expand in the coming decades.

Another striking point that appears through this overview is the variety of employed polymer substrates/scaffolds, electrical conductors, and processes, which can be used to obtain resorbable conductive materials. This is largely accounted for by not only the large range of addressed biomedical applications but also the wide panoply of available components (scaffolds and electrical conductors) that allow choosing different strategies to design and fabricate resorbable bioelectronic systems, implants, or tissue substitutes. The variety of proposed approaches in the domain is interesting in order to meet the different challenges yet to be addressed.

Technical challenges concern the fabrication processes, stretchability and conformability of the devices, as well as material conductivity. In addition, communication of the implanted device with the external world has to be adressed in terms of electric and power connectivity, as well as for eventual information transfer. Concerning the fabrication process, the adhesion between different device layers, or at the interface between metals and polymers, for instance, can still be an issue, with poor adhesion or delamination being a source of low electrical contact (and hence low device performance) and/or premature degradation. For certain applications, such as spinal cord stimulation or recording, the stretchability of the device is key. The more stretchable it is, the better it can withstand large displacements and torsions in the spine during movements. In this perspective of high stretchability/conformability to tissues, conducting polymer-based materials, thanks to their mechanical and structural properties, appear very interesting in designing electrical tracks or contact electrodes. However, their electrical conductivity is still several orders of magnitude below the ones of resorbable metals. Therefore, innovative chemical designs are still intensively sought to achieve conductive materials presenting simultaneously features of high conductivity, stretchability, and biocompatibility.

Another challenge concerns the identification of relevant medical applications that will boost scientific and technical developments, in particular in the field of resorbable implanted bioelectronic systems. For instance, if the resorbable pacemaker prototype proposed by the group of Rogers (Choi et al., 2021) is a remarkable technical achievement, doubt can exist concerning its medical relevance. However, relevant applications of resorbable implanted bioelectronic systems are foreseen in the fields of i) minimally invasive transient monitoring (wearable devices and microneedle-based devices), ii) post-surgery transient monitoring and drug delivery [e.g., for the prevention/detection of infections, the follow-up of orthopedic surgery (bone fracture and prosthesis implantation), and the assessment of flaps resection and anastomosis], and iii) electrical stimulation for the healing of the skin or internal tissue wounds, or for muscular recovery and reinforcement. At the present time, the reported studies mainly appear to be supported by technical research groups rather than by medical teams. The technical signs of progress and first preclinical proofs of concept of devices, implants, and tissue substitutes based on resorbable conductive materials will hopefully pique clinicians’ interest and will lead to the emergence of new applications of these materials.

Concerning the clinical translation challenge, in addition to the identification of relevant medical applications that are already emerging, the main task remains the demonstration of the full resorbability of devices or full integration of tissue substitutes, without any acute or long-term side effects, and the safety of the employed materials. The scaffolds/substrates and electrical conductors that have been described in Section 2 of this review have been reported as “biocompatible” and “resorbable,” but it has to be reminded that material safety has to be evaluated according to its dose, its precise preparation process, its location of implantation into the body and its residence time, its metabolization pathway, its degradation products, and possible migration away from its site of implantation. Some of the described materials are highly questionable, such as CNTs (Mishra et al., 2018) and ZnO or Zn (that will oxide in zinc oxide during its degradation process) (Xia et al., 2008; Jin et al., 2021). In presently reported studies, the resorbability of the materials is mainly assessed qualitatively by photographs or/and indirectly by the measurement of physical parameters such as impedance for an electrical contact, or a quality factor for an antenna. Safety evaluations involve the immunostaining and histology of tissue sections that come into contact with the devices or tissue substitutes. If such preliminary results, which already represent a huge amount of work, can be satisfactory in the first stage of the development of a resorbable material or device, further investigations will be required to enable the translation to the clinic. They include i) the evaluation of the dissolution rate of the material and the comparison of the employed dose with the toxicity intake limits given in meta-analysis databases such as https://pubchem.ncbi.nlm.nih.gov/; ii) the study of the metabolization pathways and identification of the degradation products of the conductive material; iii) the assessment of the biodistribution and the clearance of the material components and degradation products with time in the different organs; and iv) the study of potential side effects in the short and long terms. Another consideration to take into account is that the degradation rate of the material should be aligned as best as possible to its targeted use to minimize risks.

In conclusion, resorbable conductive materials already appear to have a bright future in biomedical applications and serve different purposes in the design of tissue substitutes and optimized interfaces between tissues and medical devices. This already very active research field should expand in the coming years, with intensive work to improve the performances of the materials to simultaneously present features of high conductivity, stretchability/conformability, and biocompatibility and to assess their safe use. These are the necessary conditions for their adoption into the clinics.

## Figures and Tables

**Table:** 

**CNT/SWCNT/MWCNT**	carbon nanotube/single-wall CNT/multiwall CNT
**β-CD**	β-cyclodextrin
**CVD**	chemical vapor deposition
**CMC**	carboxymethyl cellulose
**CMs**	cardiomyocytes
**CP**	conducting polymer
**DNA/RNA**	deoxyribonucleic acid/ribonucleic acid
**DBSA**	dodecylbenzenesulfonic acid
**ECG**	electrocardiography
**ECM**	extracellular matrix
**EDOT/PEDOT**	3,4-ethylenedioxythiophene/poly(3,4-ethylenedioxythiophene)
**GelMA**	gelatin methacrylate
**GFAP**	glial fibrillary acidic protein
**GO/rGO**	graphene oxide/reduced graphene oxide
**GOx**	glucose oxidase
**HA**	hyaluronic acid
**iPSC/hiPSC**	induced pluripotent stem cells/human induced pluripotent stem cell
**MEA**	microelectrode array
**MN**	Microneedle
**NP**	Nanoparticle
**NOAEL**	no-observed-adverse-effect limit
**NGCs**	nerve guidance conduits
**NSCs**	neuronal stem cells
**PANI**	poly(aniline)
**PBS**	Phosphate-buffered saline
**PCL**	poly(caprolactone)
**PDMS**	poly(dimethyl-siloxane)
**PEG**	poly(ethylene glycol)
**PGA**	poly(glycolic acid)
**PGS**	Poly(glycerol sebacate)
**PHA**	poly(hydroxyalkanoates)
**PHB/PHBV**	poly(3-hydroxybutyrate)/poly(3-hydroxybutyrate-co-3-hydroxyvalerate)
**PLA**	poly(lactic acid)
**PLLA**	poly(L-lactide)
**PLGA**	poly(lactic-co-glycolic acid)
**POMaC**	Poly(octamethylene maleate anhydride)
**PMMA**	poly(methyl methacrylate)
**PPy**	poly(pyrrole)
**PU**	pol(urethane)
**PSS**	poly(styrene sulfonate)
**PVA**	poly(vinyl alcohol)
**PVP**	polyvinylpyrrolidone
**TI**	tolerable intake

## References

[B1] Abidian M. R. Corey J. M. Kipke D. R. Martin D. C. (2010). Conducting-polymer nanotubes improve electrical properties, mechanical adhesion, neural attachment, and neurite outgrowth of neural electrodes. Small 6 (3), 421–429. 10.1002/smll.200901868 20077424 PMC3045566

[B2] Abidian M. R. Kim D.-H. Martin D. C. (2006). Conducting-polymer nanotubes for controlled drug release. Adv. Mater. 18 (4), 405–409. 10.1002/adma.200501726 21552389 PMC3088882

[B3] Abidian M. R. Ludwig K. A. Marzullo T. C. Martin D. C. Kipke D. R. (2009). Interfacing conducting polymer nanotubes with the central nervous system: chronic neural recording using poly(3,4-ethylenedioxythiophene) nanotubes. Adv. Mater. 21 (37), 3764–3770. 10.1002/adma.200900887 26345408 PMC4559350

[B4] Abidian M. R. Martin D. C. (2008). Experimental and theoretical characterization of implantable neural microelectrodes modified with conducting polymer nanotubes. Biomaterials 29 (9), 1273–1283. 10.1016/j.biomaterials.2007.11.022 18093644 PMC2692518

[B5] Abu Ammar A. Abdel-Haq M. Abd-Rbo K. Kasem H. (2021). Developing novel poly(lactic-Co-glycolic acid) (PLGA) films with enhanced adhesion capacity by biomimetic mushroom-shaped microstructures. Biotribology 27, 100184. 10.1016/j.biotri.2021.100184

[B6] Aghazadeh M. R. Delfanian S. Aghakhani P. Homaeigohar S. Alipour A. Shahsavarani H. (2022). Recent advances in development of natural cellulosic non-woven scaffolds for tissue engineering. Polymers 14 (8), 1531. 10.3390/polym14081531 35458282 PMC9030052

[B7] Alhashmi Alamer F. Almalki G. A. (2022). Fabrication of conductive fabrics based on SWCNTs, MWCNTs and graphene and their applications: a review. Polymers 14 (24), 5376. 10.3390/polym14245376 36559743 PMC9788045

[B8] Anand P. Llewelyn J. G. Thomas P. K. Gillon K. R. W. Lisk R. Bloom S. R. (1988). Water content, vasoactive intestinal polypeptide and substance P in intact and crushed sciatic nerves of normal and streptozotocin-diabetic rats. J. Neurological Sci. 83 (2), 167–177. 10.1016/0022-510X(88)90066-4 2451710

[B9] Ansari S. Sami N. Yasin D. Ahmad N. Fatma T. (2021). Biomedical applications of environmental friendly poly-hydroxyalkanoates. Int. J. Biol. Macromol. 183, 549–563. 10.1016/j.ijbiomac.2021.04.171 33932421

[B10] Antensteiner M. Abidian M. R. (2017b). “Tunable nanostructured conducting polymers for neural interface applications,” in Proceedings of the 39th Annual International Conference of the IEEE Engineering in Medicine and Biology Society (EMBC), Jeju Island, South Korea, July 11-15, 2017, 1881–1884. 10.1109/EMBC.2017.8037214 PMC5805560 29060258

[B11] Antensteiner M. Khorrami M. Fallahianbijan F. Borhan A. Abidian M. R. (2017a). Conducting polymer microcups for organic bioelectronics and drug delivery applications. Adv. Mater. 29 (39), 1702576. 10.1002/adma.201702576 PMC5798879 28833611

[B12] Arambula-Maldonado R. Mequanint K. (2022). Carbon-based electrically conductive materials for bone repair and regeneration. Mater. Adv. 3 (13), 5186–5206. 10.1039/d2ma00001f

[B13] Baker A. E. G. Cui H. Ballios B. G. Ing S. Yan P. Wolfer J. (2021). Stable oxime-crosslinked hyaluronan-based hydrogel as a biomimetic vitreous substitute. Biomaterials 271, 120750. 10.1016/j.biomaterials.2021.120750 33725584

[B14] Balfourier A. Luciani N. Wang G. Lelong G. Ersen O. Khelfa A. (2020). Unexpected intracellular biodegradation and recrystallization of gold nanoparticles. Proc. Natl. Acad. Sci. 117 (1), 103–113. 10.1073/pnas.1911734116 31852822 PMC6955300

[B15] Ban G. Hou Y. Shen Z. Jia J. Chai L. Ma C. (2023). Potential biomedical limitations of graphene nanomaterials. Int. J. Nanomedicine 18, 1695–1708. 10.2147/IJN.S402954 37020689 PMC10069520

[B16] Bano K. Pandey R. Roohi J. (2018). New advancements of bioplastics in medical applications. Int. J. Pharm. Sci. Res. 9 (2), 402–416. 10.13040/IJPSR.0975-8232.9(2).402-16

[B17] Bao R. Tan B. Liang S. Zhang N. Wang W. Liu W. (2017). A π-π conjugation-containing soft and conductive injectable polymer hydrogel highly efficiently rebuilds cardiac function after myocardial infarction. Biomaterials 122, 63–71. 10.1016/j.biomaterials.2017.01.012 28107665

[B18] Bashir S. Hina M. Iqbal J. Rajpar A. H. Mujtaba M. A. Alghamdi N. A. (2020). Fundamental concepts of hydrogels: synthesis, properties, and their applications. Polymers 12 (11), 2702. 10.3390/polym12112702 33207715 PMC7697203

[B19] Battigelli A. Almeida B. Shukla A. (2022). Recent advances in bioorthogonal click chemistry for biomedical applications. Bioconjugate Chem. 33 (2), 263–271. 10.1021/acs.bioconjchem.1c00564 35107252

[B20] Beygisangchin M. Abdul Rashid S. Shafie S. Sadrolhosseini A. R. Lim H. N. (2021). Preparations, properties, and applications of polyaniline and polyaniline thin films—a review. Polymers 13 (12), 2003. 10.3390/polym13122003 34207392 PMC8234317

[B21] Bhat M. A. Rather R. A. Shalla A. H. (2021). PEDOT and PEDOT:PSS conducting polymeric hydrogels: a report on their emerging applications. Synth. Met. 273, 116709. 10.1016/j.synthmet.2021.116709

[B22] Bielfeldt M. Rebl H. Peters K. Sridharan K. Staehlke S. Nebe J. B. (2022). Sensing of physical factors by cells: electric field, mechanical forces, physical plasma and light—importance for tissue regeneration. Biomed. Mater. Devices 1, 146–161. 10.1007/s44174-022-00028-x

[B23] Bilbao E. Garate O. Rodríguez Campos T. Roberti M. Mass M. Lozano A. (2023). Electrochemical sweat sensors. Chemosensors 11 (4), 244. 10.3390/chemosensors11040244

[B24] Boehler C. Agrawe Z. Asplund M. (2019). Applications of PEDOT in bioelectronic medicine. Bioelectron. Med. 2 (2), 89–99. 10.2217/bem-2019-0014

[B25] Borriello A. Guarino V. Schiavo L. Alvarez-Perez M. A. Ambrosio L. (2011). Optimizing PANi doped electroactive substrates as patches for the regeneration of cardiac muscle. J. Mater. Sci. Mater. Med. 22 (4), 1053–1062. 10.1007/s10856-011-4259-x 21373812

[B26] Borschel G. H. Kia K. F. Kuzon W. M. Dennis R. G. (2003). Mechanical properties of acellular peripheral nerve. J. Surg. Res. 114 (2), 133–139. 10.1016/S0022-4804(03)00255-5 14559438

[B27] Boutry C. M. Kaizawa Y. Schroeder B. C. Chortos A. Legrand A. Wang Z. (2018). A stretchable and biodegradable strain and pressure sensor for orthopaedic application. Nat. Electron. 1 (5), 314–321. 10.1038/s41928-018-0071-7

[B28] Boutry C. M. Müller M. Hierold C. (2012). Junctions between metals and blends of conducting and biodegradable polymers (PLLA-PPy and PCL-PPy). Mater. Sci. Eng. C 32 (6), 1610–1620. 10.1016/j.msec.2012.04.051 24364967

[B29] Caliari S. R. Burdick J. A. (2016). A practical guide to hydrogels for cell culture. Nat. Methods 13 (5), 405–414. 10.1038/nmeth.3839 27123816 PMC5800304

[B30] Cánovas R. Padrell Sánchez S. Parrilla M. Cuartero M. Crespo G. A. (2019). Cytotoxicity study of ionophore-based membranes: toward on-body and *in vivo* ion sensing. ACS Sensors 4 (9), 2524–2535. 10.1021/acssensors.9b01322 31448593

[B31] Cao Y. Wang B. (2009). Biodegradation of silk biomaterials. Int. J. Mol. Sci. 10 (4), 1514–1524. 10.3390/ijms10041514 19468322 PMC2680630

[B32] Cassano D. Summa M. Pocoví-Martínez S. Mapanao A.-K. Catelani T. Bertorelli R. (2019). Biodegradable ultrasmall-in-nano gold architectures: mid-period *in vivo* distribution and excretion assessment. Part. Part. Syst. Charact. 36 (2), 1800464. 10.1002/ppsc.201800464

[B33] Chatterjee S. Saxena M. Padmanabhan D. Jayachandra M. Pandya H. J. (2019). Futuristic medical implants using bioresorbable materials and devices. Biosens. Bioelectron. 142, 111489. 10.1016/j.bios.2019.111489 31295710

[B34] Chaudhuri O. Gu L. Darnell M. Klumpers D. Bencherif S. A. Weaver J. C. (2015). Substrate stress relaxation regulates cell spreading. Nat. Commun. 6 (1), 6365. 10.1038/ncomms7365 PMC4518451 25695512

[B35] Chaudhuri O. Gu L. Klumpers D. Darnell M. Bencherif S. A. Weaver J. C. (2016). Hydrogels with tunable stress relaxation regulate stem cell fate and activity. Nat. Mater. 15 (3), 326–334. 10.1038/nmat4489 26618884 PMC4767627

[B36] Chen C. Bai X. Ding Y. Lee I.-S. (2019). Electrical stimulation as a novel tool for regulating cell behavior in tissue engineering. Biomaterials Res. 23 (1), 25. 10.1186/s40824-019-0176-8 PMC6896676 31844552

[B37] Chen G. Matsuhisa N. Liu Z. Qi D. Cai P. Jiang Y. (2018a). Plasticizing silk protein for on-skin stretchable electrodes. Adv. Mater. 30 (21), 1800129. 10.1002/adma.201800129 29603437

[B38] Chen L. Wang W. Lin Z. Lu Y. Chen H. Li B. (2022). Conducting molybdenum sulfide/graphene oxide/polyvinyl alcohol nanocomposite hydrogel for repairing spinal cord injury. J. Nanobiotechnology 20 (1), 210. 10.1186/s12951-022-01396-8 35524268 PMC9074236

[B39] Chen X. Park Y. J. Kang M. Kang S.-K. Koo J. Shinde S. M. (2018b). CVD-grown monolayer MoS2 in bioabsorbable electronics and biosensors. Nat. Commun. 9 (1), 1690. 10.1038/s41467-018-03956-9 29703901 PMC5924366

[B40] Chen Z. Chen Y. Hedenqvist M. S. Chen C. Cai C. Li H. (2021). Multifunctional conductive hydrogels and their applications as smart wearable devices. J. Mater. Chem. B 9 (11), 2561–2583. 10.1039/d0tb02929g 33599653

[B41] Chen Z. Lin Z. Obaid S. N. Rytkin E. George S. A. Bach C. (2023). Soft, bioresorbable, transparent microelectrode arrays for multimodal spatiotemporal mapping and modulation of cardiac physiology. Sci. Adv. 9 (27), eadi0757. 10.1126/sciadv.adi0757 37406128 PMC10321742

[B42] Chiulan I. Heggset E. B. Voicu Ş. I. Chinga-Carrasco G. (2021). Photopolymerization of bio-based polymers in a biomedical engineering perspective. Biomacromolecules 22 (5), 1795–1814. 10.1021/acs.biomac.0c01745 33819022

[B43] Choi C. Choi M. K. Liu S. Kim M. Park O. K. Im C. (2017). Human eye-inspired soft optoelectronic device using high-density MoS2-graphene curved image sensor array. Nat. Commun. 8 (1), 1664. 10.1038/s41467-017-01824-6 29162854 PMC5698290

[B44] Choi C. Lee Y. Cho K. W. Koo J. H. Kim D.-H. (2019). Wearable and implantable soft bioelectronics using two-dimensional materials. Accounts Chem. Res. 52 (1), 73–81. 10.1021/acs.accounts.8b00491 30586292

[B45] Choi Y. S. Yin R. T. Pfenniger A. Koo J. Avila R. Lee K. B. (2021). Fully implantable and bioresorbable cardiac pacemakers without leads or batteries. Nat. Biotechnol. 39 (10), 1228–1238. 10.1038/s41587-021-00948-x 34183859 PMC9270064

[B46] Choi Y. Y. Ho D. H. Cho J. H. (2020). Self-healable hydrogel–liquid metal composite platform enabled by a 3D printed stamp for a multimodular sensor system. ACS Appl. Mater. Interfaces 12 (8), 9824–9832. 10.1021/acsami.9b22676 31985196

[B47] Chor A. Gonçalves R. P. Costa A. M. Farina M. Ponche A. Sirelli L. (2020). *In vitro* degradation of electrospun poly(lactic-Co-glycolic acid) (PLGA) for oral mucosa regeneration. Polymers 12 (8), 1853. 10.3390/polym12081853 32824776 PMC7465081

[B48] Christenson E. M. Anderson J. M. Hiltner A. (2007). Biodegradation mechanisms of polyurethane elastomers. Corros. Eng. Sci. Technol. 42 (4), 312–323. 10.1179/174327807x238909

[B49] Cong Y. Fu J. (2022). Hydrogel–tissue interface interactions for implantable flexible bioelectronics. Langmuir 38 (38), 11503–11513. 10.1021/acs.langmuir.2c01674 36113043

[B50] Czubacka E. Czerczak S. (2019). Are platinum nanoparticles safe to human health? Med. Pr. 70 (4), 487–495. 10.13075/mp.5893.00847 31162484

[B51] Dadras-Toussi O. Khorrami M. Louis Sam Titus A. S. C. Majd S. Mohan C. Abidian M. R. (2022). Multiphoton lithography of organic semiconductor devices for 3D printing of flexible electronic circuits, biosensors, and bioelectronics. Adv. Mater. 34 (30), 2200512. 10.1002/adma.202200512 PMC9339506 35707927

[B52] Dai C. Kong D. Chen C. Liu Y. Wei D. (2023). Graphene transistors for *in vitro* detection of health biomarkers. Adv. Funct. Mater. 33, 2301948. 10.1002/adfm.202301948

[B53] Dash M. Chiellini F. Ottenbrite R. M. Chiellini E. (2011). Chitosan—a versatile semi-synthetic polymer in biomedical applications. Prog. Polym. Sci. 36 (8), 981–1014. 10.1016/j.progpolymsci.2011.02.001

[B54] De León S. E. Pupovac A. McArthur S. L. (2020). Three-Dimensional (3D) cell culture monitoring: opportunities and challenges for impedance spectroscopy. Biotechnol. Bioeng. 117 (4), 1230–1240. 10.1002/bit.27270 31956986

[B55] Deo K. A. Jaiswal M. K. Abasi S. Lokhande G. Bhunia S. Nguyen T.-U. (2022). Nanoengineered ink for designing 3D printable flexible bioelectronics. ACS Nano 16 (6), 8798–8811. 10.1021/acsnano.1c09386 35675588 PMC13050497

[B56] Dorishetty P. Balu R. Gelmi A. Mata J. P. Quigley A. Dutta N. K. (2022). Microporosity engineered printable silk/graphene hydrogels and their cytocompatibility evaluations. Mater. Today Adv. 14, 100233. 10.1016/j.mtadv.2022.100233

[B57] Duan X. Gao R. Xie P. Cohen-Karni T. Qing Q. Choe H. S. (2012). Intracellular recordings of action potentials by an extracellular nanoscale field-effect transistor. Nat. Nanotechnol. 7 (3), 174–179. 10.1038/nnano.2011.223 PMC3293943 22179566

[B58] Eglin D. Alini M. (2008). Degradable polymeric materials for osteosynthesis: tutorial. Eur. Cells Mat. 16, 80–91. 10.22203/ecm.v016a09 19101891

[B59] Eitel I. Friedrich M. G. (2011). T2-weighted cardiovascular magnetic resonance in acute cardiac disease. J. Cardiovasc. Magnetic Reson. 13 (1), 13. 10.1186/1532-429x-13-13 PMC3060149 21332972

[B60] Elosegui-Artola A. (2021). The extracellular matrix viscoelasticity as a regulator of cell and tissue dynamics. Curr. Opin. Cell Biol. 72, 10–18. 10.1016/j.ceb.2021.04.002 33993058

[B61] eSilva E. P. Huang B. Helaehil J. V. Nalesso P. R. L. Bagne L. de Oliveira M. A. (2021). *In vivo* study of conductive 3D printed PCL/MWCNTs scaffolds with electrical stimulation for bone tissue engineering. Bio-Design Manuf. 4 (2), 190–202. 10.1007/s42242-020-00116-1

[B62] Eslamian M. Mirab F. Raghunathan V. K. Majd S. Abidian M. R. (2021). Organic semiconductor nanotubes for electrochemical devices. Adv. Funct. Mater. 31 (49), 2105358. 10.1002/adfm.202105358 34924917 PMC8673914

[B63] Fattahi P. Yang G. Kim G. Abidian M. R. (2014). A review of organic and inorganic biomaterials for neural interfaces. Adv. Mater. 26 (12), 1846–1885. 10.1002/adma.201304496 24677434 PMC4373558

[B64] Feig V. R. Tran H. Bao Z. (2018). Biodegradable polymeric materials in degradable electronic devices. ACS Central Sci. 4 (3), 337–348. 10.1021/acscentsci.7b00595 PMC5879474 29632879

[B65] Feng S. Cao S. Tian Z. Zhu H. Kong D. (2019). Maskless patterning of biodegradable conductors by selective laser sintering of microparticle inks and its application in flexible transient electronics. ACS Appl. Mater. Interfaces 11 (49), 45844–45852. 10.1021/acsami.9b14431 31718133

[B66] Ferlauto L. D’Angelo A. N. Vagni P. Airaghi Leccardi M. J. I. Mor F. M. Cuttaz E. A. (2018). Development and characterization of PEDOT:PSS/alginate soft microelectrodes for application in neuroprosthetics. Front. Neurosci. 12, 648. 10.3389/fnins.2018.00648 30283296 PMC6156361

[B67] Fleischer S. Shevach M. Feiner R. Dvir T. (2014). Coiled fiber scaffolds embedded with gold nanoparticles improve the performance of engineered cardiac tissues. Nanoscale 6 (16), 9410–9414. 10.1039/c4nr00300d 24744098

[B68] Foremny K. Nagels S. Kreienmeyer M. Doll T. Deferme W. (2021). Biocompatibility testing of liquid metal as an interconnection material for flexible implant technology. Nanomaterials 11 (12), 3251. 10.3390/nano11123251 34947600 PMC8706733

[B69] Gandhi B. Raghava N. S. (2020). Fabrication techniques for carbon nanotubes based ECG electrodes: a review. IETE J. Res., 1–20. 10.1080/03772063.2020.1768909

[B70] Gao C. Song S. Lv Y. Huang J. Zhang Z. (2022). Recent development of conductive hydrogels for tissue engineering: review and perspective. Macromol. Biosci. 22 (8), 2200051. 10.1002/mabi.202200051 35472125

[B71] GhavamiNejad P. GhavamiNejad A. Zheng H. Dhingra K. Samarikhalaj M. Poudineh M. (2023). A conductive hydrogel microneedle-based assay integrating PEDOT:PSS and Ag-Pt nanoparticles for real-time, enzyme-less, and electrochemical sensing of glucose. Adv. Healthc. Mater. 12 (1), 2202362. 10.1002/adhm.202202362 36183355

[B72] Ghosh S. Haldar S. Gupta S. Bisht A. Chauhan S. Kumar V. (2020). Anisotropically conductive biodegradable scaffold with coaxially aligned carbon nanotubes for directional regeneration of peripheral nerves. ACS Appl. Bio Mater. 3 (9), 5796–5812. 10.1021/acsabm.0c00534 35021810

[B73] Gillispie G. Prim P. Copus J. Fisher J. Mikos A. G. Yoo J. J. (2020). Assessment methodologies for extrusion-based bioink printability. Biofabrication 12 (2), 022003. 10.1088/1758-5090/ab6f0d 31972558 PMC7039534

[B74] Glasser A. Cloutet É. Hadziioannou G. Kellay H. (2019). Tuning the rheology of conducting polymer inks for various deposition processes. Chem. Mater. 31 (17), 6936–6944. 10.1021/acs.chemmater.9b01387

[B75] Gong Y. Cheng Y. Z. Hu Y. C. (2022). Preparation of polymer conductive hydrogel and its application in flexible wearable electronic devices. Prog. Chem. 34 (3), 616–629. 10.7536/pc210329

[B76] Gottschalk A. Scafidi S. Toung T. (2021). Brain water as a function of age and weight in normal rats. PLoS ONE 16 (9), e0249384. 10.1371/journal.pone.0249384 34525113 PMC8443050

[B77] Grosjean M. Gangolphe L. Nottelet B. (2023). Degradable self-healable networks for use in biomedical applications. Adv. Funct. Mater. 33 (13), 2205315. 10.1002/adfm.202205315

[B78] Gueye M. N. Carella A. Faure-Vincent J. Demadrille R. Simonato J.-P. (2020). Progress in understanding structure and transport properties of PEDOT-based materials: a critical review. Prog. Mater. Sci. 108, 100616. 10.1016/j.pmatsci.2019.100616

[B79] Guo B. Glavas L. Albertsson A.-C. (2013). Biodegradable and electrically conducting polymers for biomedical applications. Prog. Polym. Sci. 38 (9), 1263–1286. 10.1016/j.progpolymsci.2013.06.003

[B80] Guo B. Ma P. X. (2018). Conducting polymers for tissue engineering. Biomacromolecules 19 (6), 1764–1782. 10.1021/acs.biomac.8b00276 29684268 PMC6211800

[B81] Guo X. Facchetti A. (2020). The journey of conducting polymers from discovery to application. Nat. Mater. 19 (9), 922–928. 10.1038/s41563-020-0778-5 32820293

[B82] Gupta P. Agrawal A. Murali K. Varshney R. Beniwal S. Manhas S. (2019). Differential neural cell adhesion and neurite outgrowth on carbon nanotube and graphene reinforced polymeric scaffolds. Mater. Sci. Eng. C 97, 539–551. 10.1016/j.msec.2018.12.065 30678940

[B83] Gutiérrez de la Rosa S. Y. Muñiz Diaz R. Villalobos Gutiérrez P. T. Patakfalvi R. Gutiérrez Coronado Ó. (2022). Functionalized platinum nanoparticles with biomedical applications. Int. J. Mol. Sci. 23 (16), 9404. 10.3390/ijms23169404 36012670 PMC9409011

[B84] Hall C. M. Moeendarbary E. Sheridan G. K. (2021). Mechanobiology of the brain in ageing and Alzheimer's disease. Eur. J. Neurosci. 53 (12), 3851–3878. 10.1111/ejn.14766 32356339

[B85] Han W. B. Yang S. M. Rajaram K. Hwang S.-W. (2022). Materials and fabrication strategies for biocompatible and biodegradable conductive polymer composites toward bio-integrated electronic systems. Adv. Sustain. Syst. 6 (2), 2100075. 10.1002/adsu.202100075

[B86] Hébert C. Mazellier J. P. Scorsone E. Mermoux M. Bergonzo P. (2014). Boosting the electrochemical properties of diamond electrodes using carbon nanotube scaffolds. Carbon 71, 27–33. 10.1016/j.carbon.2013.12.083

[B87] Held M. Pichler A. Chabeda J. Lam N. Hindenberg P. Romero-Nieto C. (2022). Soft electronic platforms combining elastomeric stretchability and biodegradability. Adv. Sustain. Syst. 6 (2), 2100035. 10.1002/adsu.202100035

[B88] Hochberg L. R. Serruya M. D. Friehs G. M. Mukand J. A. Saleh M. Caplan A. H. (2006). Neuronal ensemble control of prosthetic devices by a human with tetraplegia. Nature 442 (7099), 164–171. 10.1038/nature04970 16838014

[B89] Hu W. Wang Z. Xiao Y. Zhang S. Wang J. (2019). Advances in crosslinking strategies of biomedical hydrogels. Biomaterials Sci. 7 (3), 843–855. 10.1039/c8bm01246f 30648168

[B90] Huang L. Yang X. Deng L. Ying D. Lu A. Zhang L. (2021). Biocompatible chitin hydrogel incorporated with PEDOT nanoparticles for peripheral nerve repair. ACS Appl. Mater. Interfaces 13 (14), 16106–16117. 10.1021/acsami.1c01904 33787211

[B91] Huang W.-J. Wang J. (2023). Development of 3D-printed, biodegradable, conductive PGSA composites for nerve tissue regeneration. Macromol. Biosci. 23 (3), 2200470. 10.1002/mabi.202200470 36525352

[B92] Huang X. Liu Y. Hwang S.-W. Kang S.-K. Patnaik D. Cortes J. F. (2014a). Biodegradable materials for multilayer transient printed circuit boards. Adv. Mater. 26 (43), 7371–7377. 10.1002/adma.201403164 25244671

[B93] Huang X.-W. Wei J.-J. Zhang M.-Y. Zhang X.-L. Yin X.-F. Lu C.-H. (2018). Water-based black phosphorus hybrid nanosheets as a moldable platform for wound healing applications. ACS Appl. Mater. Interfaces 10 (41), 35495–35502. 10.1021/acsami.8b12523 30251823

[B94] Huang Y. Li H. Hu T. Li J. Yiu C. K. Zhou J. (2022). Implantable electronic medicine enabled by bioresorbable microneedles for wireless electrotherapy and drug delivery. Nano Lett. 22 (14), 5944–5953. 10.1021/acs.nanolett.2c01997 35816764

[B95] Huang Z.-B. Yin G.-F. Liao X.-M. Gu J.-W. (2014b). Conducting polypyrrole in tissue engineering applications. Front. Mat. Sci. 8 (1), 39–45. 10.1007/s11706-014-0238-8

[B96] Humpolicek P. Kasparkova V. Saha P. Stejskal J. (2012). Biocompatibility of polyaniline. Synth. Met. 162 (7), 722–727. 10.1016/j.synthmet.2012.02.024

[B97] Hwang S.-W. Tao H. Kim D.-H. Cheng H. Song J.-K. Rill E. (2012). A physically transient form of silicon electronics. Science 337 (6102), 1640–1644. 10.1126/science.1226325 23019646 PMC3786576

[B98] Inal S. Hama A. Ferro M. Pitsalidis C. Oziat J. Iandolo D. (2017). Conducting polymer scaffolds for hosting and monitoring 3D cell culture. Adv. Biosyst. 1 (6), 1700052. 10.1002/adbi.201700052

[B99] Jacot J. G. Martin J. C. Hunt D. L. (2010). Mechanobiology of cardiomyocyte development. J. Biomechanics 43 (1), 93–98. 10.1016/j.jbiomech.2009.09.014 PMC2813357 19819458

[B100] Jensen B. E. B. Dávila I. Zelikin A. N. (2016). Poly(vinyl alcohol) physical hydrogels: matrix-mediated drug delivery using spontaneously eroding substrate. J. Phys. Chem. B 120 (26), 5916–5926. 10.1021/acs.jpcb.6b01381 26958864 PMC4939746

[B101] Jiang D.-H. Satoh T. Tung S. H. Kuo C.-C. (2022). Sustainable alternatives to nondegradable medical plastics. ACS Sustain. Chem. Eng. 10 (15), 4792–4806. 10.1021/acssuschemeng.2c00160

[B102] Jiang Y. Xu M. Yadavalli V. K. (2019). Silk fibroin-sheathed conducting polymer wires as organic connectors for biosensors. Biosensors 9 (3), 103. 10.3390/bios9030103 31466277 PMC6784353

[B103] Jin M. Li N. Sheng W. Ji X. Liang X. Kong B. (2021). Toxicity of different zinc oxide nanomaterials and dose-dependent onset and development of Parkinson’s disease-like symptoms induced by zinc oxide nanorods. Environ. Int. 146, 106179. 10.1016/j.envint.2020.106179 33099061

[B104] Jing X. Mi H.-Y. Napiwocki B. N. Peng X.-F. Turng L.-S. (2017). Mussel-inspired electroactive chitosan/graphene oxide composite hydrogel with rapid self-healing and recovery behavior for tissue engineering. Carbon 125, 557–570. 10.1016/j.carbon.2017.09.071

[B105] Kalra A. Lowe A. Al-Jumaily A. M. (2016). Mechanical behaviour of skin: a review. J. Mat. Sci. Eng. 5 (4), 1–7. 10.4172/2169-0022.1000254

[B106] Karimi A. Shojaei A. Tehrani P. (2017). Mechanical properties of the human spinal cord under the compressive loading. J. Chem. Neuroanat. 86, 15–18. 10.1016/j.jchemneu.2017.07.004 28720407

[B107] Karmakar R. S. Chu C.-P. Liao Y.-C. Lu Y.-W. (2022). PVA tactile sensors based on Electrical Contact Resistance (ECR) change mechanism for subtle pressure detection. Sensors Actuators A Phys. 342, 113613. 10.1016/j.sna.2022.113613

[B108] Katoh K. (2022). Effects of electrical stimulation on the signal transduction-related proteins, c-src and focal adhesion kinase, in fibroblasts. Life 12 (4), 531. 10.3390/life12040531 35455022 PMC9024655

[B109] Kayser L. V. Lipomi D. J. (2019). Stretchable conductive polymers and composites based on PEDOT and PEDOT:PSS. Adv. Mater. 31 (10), 1806133. 10.1002/adma.201806133 PMC6401235 30600559

[B110] Keefer E. W. Botterman B. R. Romero M. I. Rossi A. F. Gross G. W. (2008). Carbon nanotube coating improves neuronal recordings. Nat. Nanotechnol. 3 (7), 434–439. 10.1038/nnano.2008.174 18654569

[B111] Keller T. S. Mao Z. Spengler D. M. (1990). Young's modulus, bending strength, and tissue physical properties of human compact bone. J. Orthop. Res. 8 (4), 592–603. 10.1002/jor.1100080416 2355299

[B112] Khan M. A. Cantù E. Tonello S. Serpelloni M. Lopomo N. F. Sardini E. (2019). A review on biomaterials for 3D conductive scaffolds for stimulating and monitoring cellular activities. Appl. Sci. 9 (5), 961. 10.3390/app9050961

[B113] Khodagholy D. Gelinas J. N. Thesen T. Doyle W. Devinsky O. Malliaras G. G. (2015). NeuroGrid: recording action potentials from the surface of the brain. Nat. Neurosci. 18 (2), 310–315. 10.1038/nn.3905 25531570 PMC4308485

[B114] Khorrami M. Abidian M. R. (2018). “Aligned conducting polymer nanotubes for neural prostheses,” in Proceedings of the 40th Annual International Conference of the IEEE Engineering in Medicine and Biology Society (EMBC), Honolulu, HI, USA, July 18-21, 2018, 6080–6083. 10.1109/EMBC.2018.8513649 30441722

[B115] Khorrami M. Antensteiner M. Fallahianbijan F. Borhan A. Abidian M. R. (2017). “Conducting polymer microcontainers for biomedical applications,” in Proceedings of the 39th Annual International Conference of the IEEE Engineering in Medicine and Biology Society (EMBC), Jeju Island, South Korea, July 11-15, 2017, 1869–1872. 10.1109/EMBC.2017.8037211 PMC5805561 29060255

[B116] Khorshidi S. Karkhaneh A. (2018). Hydrogel/fiber conductive scaffold for bone tissue engineering. J. Biomed. Mater. Res. Part A 106 (3), 718–724. 10.1002/jbm.a.36282 29094460

[B117] Kim B. C. Spinks G. Too C. O. Wallace G. G. Bae Y. H. (2000). Preparation and characterisation of processable conducting polymer–hydrogel composites. React. Funct. Polym. 44 (1), 31–40. 10.1016/S1381-5148(99)00074-7

[B118] Kim D.-H. Kim Y.-S. Amsden J. Panilaitis B. Kaplan D. L. Omenetto F. G. (2009). Silicon electronics on silk as a path to bioresorbable, implantable devices. Appl. Phys. Lett. 95 (13), 133701. 10.1063/1.3238552 20145699 PMC2816979

[B119] Kim D.-H. Viventi J. Amsden J. J. Xiao J. Vigeland L. Kim Y.-S. (2010). Dissolvable films of silk fibroin for ultrathin conformal bio-integrated electronics. Nat. Mater. 9 (6), 511–517. 10.1038/nmat2745 20400953 PMC3034223

[B120] Kim J. Campbell A. S. de Ávila B. E.-F. Wang J. (2019). Wearable biosensors for healthcare monitoring. Nat. Biotechnol. 37 (4), 389–406. 10.1038/s41587-019-0045-y 30804534 PMC8183422

[B121] Kim K. S. Maeng W.-Y. Kim S. Lee G. Hong M. Kim G.-B. (2023a). Isotropic conductive paste for bioresorbable electronics. Mater. Today Bio 18, 100541. 10.1016/j.mtbio.2023.100541 PMC9840151 36647537

[B122] Kim S. Baek S. Sluyter R. Konstantinov K. Kim J. H. Kim S. (2023b). Wearable and implantable bioelectronics as eco-friendly and patient-friendly integrated nanoarchitectonics for next-generation smart healthcare technology. EcoMat 5, e12356. 10.1002/eom2.12356

[B123] Knopf-Marques H. Pravda M. Wolfova L. Velebny V. Schaaf P. Vrana N. E. (2016). Hyaluronic acid and its derivatives in coating and delivery systems: applications in tissue engineering, regenerative medicine and immunomodulation. Adv. Healthc. Mat. 5, 2841–2855. 10.1002/adhm.201600316 27709832

[B124] Kobayashi T. Chanmee T. Itano N. (2020). Hyaluronan: metabolism and function. Biomolecules 10 (11), 1525. 10.3390/biom10111525 33171800 PMC7695009

[B125] Koivusalo L. Kauppila M. Samanta S. Parihar V. S. Ilmarinen T. Miettinen S. (2019). Tissue adhesive hyaluronic acid hydrogels for sutureless stem cell delivery and regeneration of corneal epithelium and stroma. Biomaterials 225, 119516. 10.1016/j.biomaterials.2019.119516 31574405

[B126] Koller M. (2018). Biodegradable and biocompatible polyhydroxy-alkanoates (PHA): auspicious microbial macromolecules for pharmaceutical and therapeutic *applications* . Molecules 23 (2), 362. 10.3390/molecules23020362 29419813 PMC6017587

[B127] Korupalli C. Li H. Nguyen N. Mi F.-L. Chang Y. Lin Y.-J. (2021). Conductive materials for healing wounds: their incorporation in electroactive wound dressings, characterization, and perspectives. Adv. Healthc. Mater. 10 (6), 2001384. 10.1002/adhm.202001384 33274846

[B128] Kozai T. D. Y. Jaquins-Gerstl A. S. Vazquez A. L. Michael A. C. Cui X. T. (2015). Brain tissue responses to neural implants impact signal sensitivity and intervention strategies. ACS Chem. Neurosci. 6 (1), 48–67. 10.1021/cn500256e 25546652 PMC4304489

[B129] Kozlowski M. T. Crook C. J. Ku H. T. (2021). Towards organoid culture without Matrigel. Commun. Biol. 4 (1), 1387. 10.1038/s42003-021-02910-8 34893703 PMC8664924

[B130] Kurakula M. Rao G. S. N. K. (2020). Pharmaceutical assessment of polyvinylpyrrolidone (PVP): as excipient from conventional to controlled delivery systems with a spotlight on COVID-19 inhibition. J. Drug Deliv. Sci. Technol. 60, 102046. 10.1016/j.jddst.2020.102046 32905026 PMC7462970

[B131] Kurland N. E. Dey T. Kundu S. C. Yadavalli V. K. (2013). Precise patterning of silk microstructures using Photolithography. Adv. Mater. 25 (43), 6207–6212. 10.1002/adma.201302823 24038619

[B132] Lee G. Ray E. Yoon H.-J. Genovese S. Choi Y. S. Lee M.-K. (2022). A bioresorbable peripheral nerve stimulator for electronic pain block. Sci. Adv. 8 (40), eabp9169. 10.1126/sciadv.abp9169 36197971 PMC9534494

[B133] Lee J. Manoharan V. Cheung L. Lee S. Cha B.-H. Newman P. (2019). Nanoparticle-based hybrid scaffolds for deciphering the role of multimodal cues in cardiac tissue engineering. ACS Nano 13 (11), 12525–12539. 10.1021/acsnano.9b03050 31621284 PMC7068777

[B134] Lei H. Fan D. (2021). Conductive, adaptive, multifunctional hydrogel combined with electrical stimulation for deep wound repair. Chem. Eng. J. 421, 129578. 10.1016/j.cej.2021.129578

[B135] Lei T. Guan M. Liu J. Lin H.-C. Pfattner R. Shaw L. (2017). Biocompatible and totally disintegrable semiconducting polymer for ultrathin and ultralightweight transient electronics. Proc. Natl. Acad. Sci. 114 (20), 5107–5112. 10.1073/pnas.1701478114 28461459 PMC5441761

[B136] Leprince M. Mailley P. Choisnard L. Auzély-Velty R. Texier I. (2023a). Design of hyaluronan-based dopant for conductive and resorbable PEDOT ink. Carbohydr. Polym. 301, 120345. 10.1016/j.carbpol.2022.120345 36446494

[B137] Leprince M. Regal S. Mailley P. Sauter-Starace F. Texier I. Auzély-Velty R. (2023b). A cross-linkable and resorbable PEDOT-based ink using a hyaluronic acid derivative as dopant for flexible bioelectronic devices. Mater. Adv. 4 (16), 3636–3644. 10.1039/d3ma00170a

[B138] Li C. Guan G. Reif R. Huang Z. Wang R. K. (2012). Determining elastic properties of skin by measuring surface waves from an impulse mechanical stimulus using phase-sensitive optical coherence tomography. J. R. Soc. Interface 9 (70), 831–841. 10.1098/rsif.2011.0583 22048946 PMC3306653

[B139] Li J. Fang L. Tait W. R. Sun L. Zhao L. Qian L. (2017). Preparation of conductive composite hydrogels from carboxymethyl cellulose and polyaniline with a nontoxic crosslinking agent. RSC Adv. 7 (86), 54823–54828. 10.1039/c7ra10788a

[B140] Li M. Liang Y. He J. Zhang H. Guo B. (2020). Two-pronged strategy of biomechanically active and biochemically multifunctional hydrogel wound dressing to accelerate wound closure and wound healing. Chem. Mater. 32 (23), 9937–9953. 10.1021/acs.chemmater.0c02823

[B141] Li Y. He J. Zhou J. Li Z. Liu L. Hu S. (2022). A conductive photothermal non-swelling nanocomposite hydrogel patch accelerating bone defect repair. Biomaterials Sci. 10 (5), 1326–1341. 10.1039/d1bm01937f 35103257

[B142] Liang B. Fang L. Hu Y. Yang G. Zhu Q. Ye X. (2014). Fabrication and application of flexible graphene silk composite film electrodes decorated with spiky Pt nanospheres. Nanoscale 6 (8), 4264–4274. 10.1039/c3nr06057h 24615460

[B143] Liang S. Zhang Y. Wang H. Xu Z. Chen J. Bao R. (2018). Paintable and rapidly bondable conductive hydrogels as therapeutic cardiac patches. Adv. Mater. 30 (23), 1704235. 10.1002/adma.201704235 29687502

[B144] Liang Y. Zhao X. Hu T. Chen B. Yin Z. Ma P. X. (2019). Adhesive hemostatic conducting injectable composite hydrogels with sustained drug release and photothermal antibacterial activity to promote full-thickness skin regeneration during wound healing. Small 15 (12), 1900046. 10.1002/smll.201900046 30786150

[B145] Liu C. Kim J. T. Yang D. S. Cho D. H. Yoo S. Madhvapathy S. R. (2023a). Multifunctional materials strategies for enhanced safety of wireless, skin-interfaced bioelectronic devices. Adv. Funct. Mater. 33. 10.1002/adfm.202302256

[B146] Liu D. Huyan C. Wang Z. Guo Z. Zhang X. Torun H. (2023b). Conductive polymer based hydrogels and their application in wearable sensors: a review. Mater. Horizons 10, 2800–2823. 10.1039/d3mh00056g 37204005

[B147] Liu H. Feng Y. Che S. Guan L. Yang X. Zhao Y. (2023c). An electroconductive hydrogel scaffold with injectability and biodegradability to manipulate neural stem cells for enhancing spinal cord injury repair. Biomacromolecules 24 (1), 86–97. 10.1021/acs.biomac.2c00920 36512504

[B148] Liu J. Appaix F. Bibari O. Marchand G. Benabid A.-L. Sauter-Starace F. (2011). Control of neuronal network organization by chemical surface functionalization of multi-walled carbon nanotube arrays. Nanotechnology 22 (19), 195101. 10.1088/0957-4484/22/19/195101 21436508 PMC3103516

[B149] Liu J. Fu T.-M. Cheng Z. Hong G. Zhou T. Jin L. (2015). Syringe-injectable electronics. Nat. Nanotechnol. 10 (7), 629–636. 10.1038/nnano.2015.115 26053995 PMC4591029

[B150] Liu J. Xie C. Dai X. Jin L. Zhou W. Lieber C. M. (2013). Multifunctional three-dimensional macroporous nanoelectronic networks for smart materials. Proc. Natl. Acad. Sci. 110 (17), 6694–6699. 10.1073/pnas.1305209110 23569270 PMC3637762

[B151] Lu Y. Cheng D. Niu B. Wang X. Wu X. Wang A. (2023a). Properties of poly (Lactic-co-Glycolic acid) and progress of poly (Lactic-co-Glycolic acid)-based biodegradable materials in biomedical research. Pharmaceuticals 16 (3), 454. 10.3390/ph16030454 36986553 PMC10058621

[B152] Ludwig K. A. Uram J. D. Yang J. Martin D. C. Kipke D. R. (2006). Chronic neural recordings using silicon microelectrode arrays electrochemically deposited with a poly(3,4-ethylenedioxythiophene) (PEDOT) film. J. Neural Eng. 3 (1), 59–70. 10.1088/1741-2560/3/1/007 16510943

[B153] Luo J. Yang J. Zheng X. Ke X. Chen Y. Tan H. (2020). A highly stretchable, real-time self-healable hydrogel adhesive matrix for tissue patches and flexible electronics. Adv. Healthc. Mater. 9 (4), 1901423. 10.1002/adhm.201901423 31945276

[B154] Luo Y. Fan L. Liu C. Wen H. Wang S. Guan P. (2022). An injectable, self-healing, electroconductive extracellular matrix-based hydrogel for enhancing tissue repair after traumatic spinal cord injury. Bioact. Mater. 7, 98–111. 10.1016/j.bioactmat.2021.05.039 34466720 PMC8379448

[B155] Lycke R. Kim R. Zolotavin P. Montes J. Sun Y. Koszeghy A. (2023). Low-threshold, high-resolution, chronically stable intracortical microstimulation by ultraflexible electrodes. Cell Rep. 42 (6), 112554. 10.1016/j.celrep.2023.112554 37235473 PMC10592461

[B156] Ma B. Martín C. Kurapati R. Bianco A. (2020). Degradation-by-design: how chemical functionalization enhances the biodegradability and safety of 2D materials. Chem. Soc. Rev. 49 (17), 6224–6247. 10.1039/c9cs00822e 32724940

[B157] Manousiouthakis E. Park J. Hardy J. G. Lee J. Y. Schmidt C. E. (2022). Towards the translation of electroconductive organic materials for regeneration of neural tissues. Acta Biomater. 139, 22–42. 10.1016/j.actbio.2021.07.065 34339871

[B158] Mao J. Zhang Z. (2018). “Polypyrrole as electrically conductive biomaterials: synthesis, biofunctionalization, potential applications and challenges,” in Cutting-edge enabling technologies for regenerative medicine. Editors Chun H. J. Park C. H. Kwon I. K. Khang G. (Singapore: Springer Singapore), 347–370. 10.1007/978-981-13-0950-2_18 30357632

[B159] Mbori N. J. R. Chuan X. Y. Feng Q. X. Alizada M. Zhan J. (2016). Evaluation of the combination of methylprednisolone and tranilast after spinal cord injury in rat models. J. Korean Neurosurg. Soc. 59 (4), 334–340. 10.3340/jkns.2016.59.4.334 27446512 PMC4954879

[B160] Min J. H. Patel M. Koh W.-G. (2018). Incorporation of conductive materials into hydrogels for tissue engineering applications. Polymers 10 (10), 1078. 10.3390/polym10101078 30961003 PMC6404001

[B161] Mishra V. Kesharwani P. Jain N. K. (2018). Biomedical applications and toxicological aspects of functionalized carbon nanotubes. Crit. Reviews™ Ther. Drug Carr. Syst. 35 (4), 293–330. 10.1615/CritRevTherDrugCarrierSyst.2018014419 29972680

[B162] Miyata S. Kitagawa H. (2017). Formation and remodeling of the brain extracellular matrix in neural plasticity: roles of chondroitin sulfate and hyaluronan. Biochim. Biophys. Acta 1861 (10), 2420–2434. 10.1016/j.bbagen.2017.06.010 28625420

[B163] Morgan E. F. Unnikrisnan G. U. Hussein A. I. (2018). Bone mechanical properties in healthy and diseased states. Annu. Rev. Biomed. Eng. 20 (1), 119–143. 10.1146/annurev-bioeng-062117-121139 29865872 PMC6053074

[B164] Morgan F. L. C. Fernández-Pérez J. Moroni L. Baker M. B. (2022). Tuning hydrogels by mixing dynamic cross-linkers: enabling cell-instructive hydrogels and advanced bioinks. Adv. Healthc. Mater. 11 (1), 2101576. 10.1002/adhm.202101576 PMC11468463 34614297

[B165] Morsink M. Severino P. Luna-Ceron E. Hussain M. A. Sobahi N. Shin S. R. (2022). Effects of electrically conductive nano-biomaterials on regulating cardiomyocyte behavior for cardiac repair and regeneration. Acta Biomater. 139, 141–156. 10.1016/j.actbio.2021.11.022 34818579 PMC11041526

[B166] Mostafavi E. Medina-Cruz D. Kalantari K. Taymoori A. Soltantabar P. Webster T. J. (2020). Electroconductive nanobiomaterials for tissue engineering and regenerative medicine. Bioelectricity 2 (2), 120–149. 10.1089/bioe.2020.0021 34471843 PMC8370325

[B167] Mueller E. Poulin I. Bodnaryk W. J. Hoare T. (2022). Click chemistry hydrogels for extrusion bioprinting: progress, challenges, and opportunities. Biomacromolecules 23 (3), 619–640. 10.1021/acs.biomac.1c01105 34989569

[B168] Nair L. S. Laurencin C. T. (2007). Biodegradable polymers as biomaterials. Prog. Polym. Sci. 32 (8), 762–798. 10.1016/j.progpolymsci.2007.05.017

[B169] Namsheer K. Rout C. S. (2021). Conducting polymers: a comprehensive review on recent advances in synthesis, properties and applications. RSC Adv. 11 (10), 5659–5697. 10.1039/d0ra07800j 35686160 PMC9133880

[B170] Nezakati T. Seifalian A. Tan A. Seifalian A. M. (2018). Conductive polymers: opportunities and challenges in biomedical applications. Chem. Rev. 118 (14), 6766–6843. 10.1021/acs.chemrev.6b00275 29969244

[B171] Nie S. Li Z. Yao Y. Jin Y. (2021). Progress in synthesis of conductive polymer poly(3,4-ethylenedioxythiophene). Front. Chem. 9, 803509. 10.3389/fchem.2021.803509 35004622 PMC8738075

[B172] O’Brien T. D. Reeves N. D. Baltzopoulos V. Jones D. A. Maganaris C. N. (2010). Mechanical properties of the patellar tendon in adults and children. J. Biomechanics 43 (6), 1190–1195. 10.1016/j.jbiomech.2009.11.028 20045111

[B173] Onorato J. W. Luscombe C. K. (2019). Morphological effects on polymeric mixed ionic/electronic conductors. Mol. Syst. Des. Eng. 4 (2), 310–324. 10.1039/c8me00093j

[B174] Pal R. K. Farghaly A. A. Collinson M. M. Kundu S. C. Yadavalli V. K. (2016). Photolithographic micropatterning of conducting polymers on flexible silk matrices. Adv. Mater. 28 (7), 1406–1412. 10.1002/adma.201504736 26641445

[B175] Palma M. Khoshnevis M. Lion M. Zenga C. Kefs S. Fallegger F. (2022). Chronic recording of cortical activity underlying vocalization in awake minipigs. J. Neurosci. Methods 366, 109427. 10.1016/j.jneumeth.2021.109427 34852254

[B176] Palmisano F. Malitesta C. Centonze D. Zambonin P. G. (1995). Correlation between permselectivity and chemical-structure of overoxidized polypyrrole membranes used in electroproduced enzyme biosensors. Anal. Chem. 67, 2207–2211. 10.1021/ac00109a046

[B177] Palumbo A. Li Z. Yang E. H. (2022). Trends on carbon nanotube-based flexible and wearable sensors via electrochemical and mechanical stimuli: a review. IEEE Sensors J. 22 (21), 20102–20125. 10.1109/jsen.2022.3198847

[B178] Pan Z. Ding J. (2012). Poly(lactide-co-glycolide) porous scaffolds for tissue engineering and regenerative medicine. Interface Focus 2 (3), 366–377. 10.1098/rsfs.2011.0123 23741612 PMC3363019

[B179] Park C. Kim M. S. Kim H. H. Sunwoo S. H. Jung D. J. Choi M. K. (2022a). Stretchable conductive nanocomposites and their applications in wearable devices. Appl. Phys. Rev. 9 (2). 10.1063/5.0093261

[B180] Park D.-W. Schendel A. A. Mikael S. Brodnick S. K. Richner T. J. Ness J. P. (2014a). Graphene-based carbon-layered electrode array technology for neural imaging and optogenetic applications. Nat. Commun. 5 (1), 5258. 10.1038/ncomms6258 25327513 PMC4218963

[B181] Park J. Jeon J. Kim B. Lee M. S. Park S. Lim J. (2020). Electrically conductive hydrogel nerve guidance conduits for peripheral nerve regeneration. Adv. Funct. Mater. 30 (39), 2003759. 10.1002/adfm.202003759

[B182] Park S. Abidian M. R. Majd S. (2017). “Micro-patterned films of bio-functionalized conducting polymers for cellular engineering,” in Proceedings of the 39th Annual International Conference of the IEEE Engineering in Medicine and Biology Society (EMBC), Jeju Island, South Korea, July 11-15, 2017, 1595–1598. 10.1109/EMBC.2017.8037143 29060187

[B183] Park S. Yang G. Madduri N. Abidian M. R. Majd S. (2014b). Hydrogel-mediated direct patterning of conducting polymer films with multiple surface chemistries. Adv. Mater. 26 (18), 2782–2787. 10.1002/adma.201306093 24623531 PMC5805559

[B184] Park Y. Chung T. S. Lee G. Rogers J. A. (2022b). Materials chemistry of neural interface technologies and recent advances in three-dimensional systems. Chem. Rev. 122 (5), 5277–5316. 10.1021/acs.chemrev.1c00639 34739219

[B185] Park Y. Franz C. K. Ryu H. Luan H. Cotton K. Y. Kim J. U. (2021). Three-dimensional, multifunctional neural interfaces for cortical spheroids and engineered assembloids. Sci. Adv. 7 (12), eabf9153. 10.1126/sciadv.abf9153 33731359 PMC7968849

[B186] Piarali S. Marlinghaus L. Viebahn R. Lewis H. Ryadnov M. G. Groll J. (2020). Activated polyhydroxyalkanoate meshes prevent bacterial adhesion and biofilm development in regenerative medicine applications. Front. Bioeng. Biotechnol. 8, 442. 10.3389/fbioe.2020.00442 32671021 PMC7326089

[B187] Puiggalí-Jou A. Cazorla E. Ruano G. Babeli I. Ginebra M.-P. García-Torres J. (2020). Electroresponsive alginate-based hydrogels for controlled release of hydrophobic drugs. ACS Biomaterials Sci. Eng. 6 (11), 6228–6240. 10.1021/acsbiomaterials.0c01400 33449669

[B188] Pyarasani R. D. Jayaramudu T. John A. (2019). Polyaniline-based conducting hydrogels. J. Mater. Sci. 54 (2), 974–996. 10.1007/s10853-018-2977-x

[B189] Rahimi R. Shams Es-haghi S. Chittiboyina S. Mutlu Z. Lelièvre S. A. Cakmak M. (2018). Laser-enabled processing of stretchable electronics on a hydrolytically degradable hydrogel. Adv. Healthc. Mater. 7 (16), 1800231. 10.1002/adhm.201800231 29947042

[B190] Rai R. Roether J. A. Boccaccini A. R. (2022). Polyaniline based polymers in tissue engineering applications: a review. Prog. Biomed. Eng. 4 (4), 042004. 10.1088/2516-1091/ac93d3

[B191] Rai R. Tallawi M. Grigore A. Boccaccini A. R. (2012). Synthesis, properties and biomedical applications of poly(glycerol sebacate) (PGS): a review. Prog. Polym. Sci. 37 (8), 1051–1078. 10.1016/j.progpolymsci.2012.02.001

[B192] Ramasamy S. M. Bhaskar R. Narayanan K. B. Purohit S. D. Park S. S. Manikkavel A. (2022). Combination of polydopamine and carbon nanomaterials coating enhances the piezoelectric responses and cytocompatibility of biodegradable PLLA nanofiber scaffolds for tissue engineering applications. Mater. Today Commun. 33, 104659. 10.1016/j.mtcomm.2022.104659

[B193] Ravichandran R. Martinez J. G. Jager E. W. H. Phopase J. Turner A. P. F. (2018). Type I collagen-derived injectable conductive hydrogel scaffolds as glucose sensors. ACS Appl. Mater. Interfaces 10 (19), 16244–16249. 10.1021/acsami.8b04091 29701457

[B194] Richardson-Burns S. M. Hendricks J. L. Foster B. Povlich L. K. Kim D.-H. Martin D. C. (2007). Polymerization of the conducting polymer poly(3,4-ethylenedioxythiophene) (PEDOT) around living neural cells. Biomaterials 28 (8), 1539–1552. 10.1016/j.biomaterials.2006.11.026 17169420 PMC1941689

[B195] Rivnay J. Wang H. Fenno L. Deisseroth K. Malliaras G. G. (2017). Next-generation probes, particles, and proteins for neural interfacing. Sci. Adv. 3 (6), e1601649. 10.1126/sciadv.1601649 28630894 PMC5466371

[B196] Robinson J. T. Jorgolli M. Shalek A. K. Yoon M.-H. Gertner R. S. Park H. (2012). Vertical nanowire electrode arrays as a scalable platform for intracellular interfacing to neuronal circuits. Nat. Nanotechnol. 7 (3), 180–184. 10.1038/nnano.2011.249 22231664 PMC4209482

[B197] Rogers Z. J. Zeevi M. P. Koppes R. Bencherif S. A. (2020). Electroconductive hydrogels for tissue engineering: current status and future perspectives. Bioelectricity 2 (3), 279–292. 10.1089/bioe.2020.0025 34476358 PMC8370338

[B198] Roshanbinfar K. Vogt L. Greber B. Diecke S. Boccaccini A. R. Scheibel T. (2018). Electroconductive biohybrid hydrogel for enhanced maturation and beating properties of engineered cardiac tissues. Adv. Funct. Mater. 28 (42), 1803951. 10.1002/adfm.201803951

[B199] Rosso G. Guck J. (2019). Mechanical changes of peripheral nerve tissue microenvironment and their structural basis during development. Apl. Bioeng. 3 (3), 036107. 10.1063/1.5108867 31893255 PMC6932855

[B200] Rousche P. J. Normann R. A. (1998). Chronic recording capability of the Utah Intracortical Electrode Array in cat sensory cortex. J. Neurosci. Methods 82 (1), 1–15. 10.1016/S0165-0270(98)00031-4 10223510

[B201] Roy S. David-Pur M. Hanein Y. (2017). Carbon nanotube-based ion selective sensors for wearable applications. ACS Appl. Mater. Interfaces 9 (40), 35169–35177. 10.1021/acsami.7b07346 28925684

[B202] Saghebasl S. Akbarzadeh A. Gorabi A. M. Nikzamir N. SeyedSadjadi M. Mostafavi E. (2022). Biodegradable functional macromolecules as promising scaffolds for cardiac tissue engineering. Polym. Adv. Technol. 33 (7), 2044–2068. 10.1002/pat.5669

[B203] Salehi M. Naseri-Nosar M. Ebrahimi-Barough S. Nourani M. Khojasteh A. Hamidieh A.-A. (2018). Sciatic nerve regeneration by transplantation of Schwann cells via erythropoietin controlled-releasing polylactic acid/multiwalled carbon nanotubes/gelatin nanofibrils neural guidance conduit. J. Biomed. Mater. Res. Part B Appl. Biomaterials 106 (4), 1463–1476. 10.1002/jbm.b.33952 28675568

[B204] Samanta S. Ylä-Outinen L. Rangasami V. K. Narkilahti S. Oommen O. P. (2022). Bidirectional cell-matrix interaction dictates neuronal network formation in a brain-mimetic 3D scaffold. Acta Biomater. 140, 314–323. 10.1016/j.actbio.2021.12.010 34902615

[B205] Sansiñena J. M. Olazábal V. Otero T. F. Sansiñena J. M. Polo da Fonseca C. N. De Paoli M. A. (1997). A solid state artificial muscle based on polypyrrole and a solid polymeric electrolyte working in air. Chem. Commun., 2217–2218. 10.1039/A705341J

[B206] Saravanan S. Sareen N. Abu-El-Rub E. Ashour H. Sequiera G. L. Ammar H. I. (2018). Graphene oxide-gold nanosheets containing chitosan scaffold improves ventricular contractility and function after implantation into infarcted heart. Sci. Rep. 8 (1), 15069. 10.1038/s41598-018-33144-0 30305684 PMC6180127

[B207] Sauter-Starace F. Torres-Martinez N. Agache V. Pudda C. Dijon J. Piallat B. (2011). “Epileptic seizure recordings of a non-human primate using carbon nanotube microelectrodes on implantable silicon shanks,” in 2011 5th International IEEE/EMBS Conference on Neural Engineering, Cancun, Mexico, April 2011.

[B208] Schemitsch E. H. (2017). Size matters: defining critical in bone defect size. J. Orthop. Trauma 31, S20–S22. 10.1097/bot.0000000000000978 28938386

[B209] Shafique H. de Vries J. Strauss J. Khorrami Jahromi A. Siavash Moakhar R. Mahshid S. (2023). Advances in the translation of electrochemical hydrogel-based sensors. Adv. Healthc. Mater. 12 (1), 2201501. 10.1002/adhm.202201501 36300601

[B210] Shaner S. Savelyeva A. Kvartuh A. Jedrusik N. Matter L. Leal J. (2023). Bioelectronic microfluidic wound healing: a platform for investigating direct current stimulation of injured cell collectives. Lab a Chip 23 (6), 1531–1546. 10.1039/d2lc01045c PMC10013350 36723025

[B211] Shin J. Yan Y. Bai W. Xue Y. Gamble P. Tian L. (2019). Bioresorbable pressure sensors protected with thermally grown silicon dioxide for the monitoring of chronic diseases and healing processes. Nat. Biomed. Eng. 3 (1), 37–46. 10.1038/s41551-018-0300-4 30932064

[B212] Shou Y. Teo X. Y. Wu K. Z. Bai B. Kumar A. R. K. Low J. (2023). Dynamic stimulations with bioengineered extracellular matrix-mimicking hydrogels for mechano cell reprogramming and therapy. Adv. Sci. 10, 2300670. 10.1002/advs.202300670 PMC10375194 37119518

[B213] Singh A. K. Srivastava J. K. Chandel A. K. Sharma L. Mallick N. Singh S. P. (2019). Biomedical applications of microbially engineered polyhydroxyalkanoates: an insight into recent advances, bottlenecks, and solutions. Appl. Microbiol. Biotechnol. 103 (5), 2007–2032. 10.1007/s00253-018-09604-y 30645689

[B214] Solazzo M. O'Brien F. Nicolosi V. Monaghan M. (2019). The rationale and emergence of electroconductive biomaterial scaffolds in cardiac tissue engineering. Apl. Bioeng. 3 (4), 041501. 10.1063/1.5116579 31650097 PMC6795503

[B215] Sordini L. Garrudo F. F. F. Rodrigues C. A. V. Linhardt R. J. Cabral J. M. S. Ferreira F. C. (2021). Effect of electrical stimulation conditions on neural stem cells differentiation on cross-linked PEDOT:PSS films. Front. Bioeng. Biotechnol. 9, 591838. 10.3389/fbioe.2021.591838 33681153 PMC7928331

[B216] Su W.-Y. Chen Y.-C. Lin F.-H. (2010). Injectable oxidized hyaluronic acid/adipic acid dihydrazide hydrogel for nucleus pulposus regeneration. Acta Biomater. 6 (8), 3044–3055. 10.1016/j.actbio.2010.02.037 20193782

[B217] Sun Y. Liu X. George M. N. Park S. Gaihre B. Terzic A. (2021). Enhanced nerve cell proliferation and differentiation on electrically conductive scaffolds embedded with graphene and carbon nanotubes. J. Biomed. Mater. Res. Part A 109 (2), 193–206. 10.1002/jbm.a.37016 32441388

[B218] Sunwoo S.-H. Ha K.-H. Lee S. Lu N. Kim D.-H. (2021). Wearable and implantable soft bioelectronics: device designs and material strategies. Annu. Rev. Chem. Biomol. Eng. 12 (1), 359–391. 10.1146/annurev-chembioeng-101420-024336 34097846

[B219] Sunwoo S.-H. Han S. I. Joo H. Cha G. D. Kim D. Choi S. H. (2020). Advances in soft bioelectronics for brain research and clinical neuroengineering. Matter 3 (6), 1923–1947. 10.1016/j.matt.2020.10.020

[B220] Surowiec R. K. Allen M. R. Wallace J. M. (2022). Bone hydration: how we can evaluate it, what can it tell us, and is it an effective therapeutic target? Bone Rep. 16, 101161. 10.1016/j.bonr.2021.101161 35005101 PMC8718737

[B221] Tao H. Hwang S.-W. Marelli B. An B. Moreau J. E. Yang M. (2014). Silk-based resorbable electronic devices for remotely controlled therapy and *in vivo* infection abatement. Proc. Natl. Acad. Sci. 111 (49), 17385–17389. 10.1073/pnas.1407743111 25422476 PMC4267401

[B222] Tebaldi M. L. Maia A. L. C. Poletto F. de Andrade F. V. Soares D. C. F. (2019). Poly(-3-hydroxybutyrate-co-3-hydroxyvalerate) (PHBV): current advances in synthesis methodologies, antitumor applications and biocompatibility. J. Drug Deliv. Sci. Technol. 51, 115–126. 10.1016/j.jddst.2019.02.007

[B223] Téllez-Soto C. A. Pereira Silva M. G. dos Santos L. de O. Mendes T. Singh P. (2021). *In vivo* determination of dermal water content in chronological skin aging by confocal Raman spectroscopy. Vib. Spectrosc. 112, 103196. 10.1016/j.vibspec.2020.103196

[B224] Thrivikraman G. Boda S. K. Basu B. (2018). Unraveling the mechanistic effects of electric field stimulation towards directing stem cell fate and function: a tissue engineering perspective. Biomaterials 150, 60–86. 10.1016/j.biomaterials.2017.10.003 29032331

[B225] Tran V. V. Lee S. Lee D. Le T.-H. (2022). Recent developments and implementations of conductive polymer-based flexible devices in sensing applications. Polymers 14 (18), 3730. 10.3390/polym14183730 36145876 PMC9504310

[B226] Tringides C. M. Boulingre M. Khalil A. Lungjangwa T. Jaenisch R. Mooney D. J. (2023). Tunable conductive hydrogel scaffolds for neural cell differentiation. Adv. Healthc. Mater. 12 (7), 2202221. 10.1002/adhm.202202221 PMC10359022 36495560

[B227] Tringides C. M. Mooney D. J. (2022). Materials for implantable surface electrode arrays: current status and future directions. Adv. Mater. 34 (20), 2107207. 10.1002/adma.202107207 34716730

[B228] Tringides C. M. Vachicouras N. de Lázaro I. Wang H. Trouillet A. Seo B. R. (2021). Viscoelastic surface electrode arrays to interface with viscoelastic tissues. Nat. Nanotechnol. 16 (9), 1019–1029. 10.1038/s41565-021-00926-z 34140673 PMC9233755

[B229] Tropp J. Rivnay J. (2021). Design of biodegradable and biocompatible conjugated polymers for bioelectronics. J. Mater. Chem. C 9 (39), 13543–13556. 10.1039/d1tc03600a

[B230] Turner B. Ramesh S. Menegatti S. Daniele M. (2022). Resorbable elastomers for implantable medical devices: highlights and applications. Polym. Int. 71 (5), 552–561. 10.1002/pi.6349

[B231] Ulery B. D. Nair L. S. Laurencin C. T. (2011). Biomedical applications of biodegradable polymers. J. Polym. Sci. Part B Polym. Phys. 49 (12), 832–864. 10.1002/polb.22259 PMC3136871 21769165

[B232] Ullah M. W. Fu L. Lamboni L. Shi Z. Yang G. (2019). “Chapter 3 - current trends and biomedical applications of resorbable polymers,” in Materials for biomedical engineering. Editors Grumezescu V. Grumezescu A. M. (Amsterdam, Netherlands: Elsevier), 41–86.

[B233] Vasvani S. Kulkarni P. Rawtani D. (2020). Hyaluronic acid: a review on its biology, aspects of drug delivery, route of administrations and a special emphasis on its approved marketed products and recent clinical studies. Int. J. Biol. Macromol. 151, 1012–1029. 10.1016/j.ijbiomac.2019.11.066 31715233

[B234] Veletić M. Apu E. H. Simić M. Bergsland J. Balasingham I. Contag C. H. (2022). Implants with sensing capabilities. Chem. Rev. 122 (21), 16329–16363. 10.1021/acs.chemrev.2c00005 35981266

[B235] Vijayavenkataraman S. (2020). Nerve guide conduits for peripheral nerve injury repair: a review on design, materials and fabrication methods. Acta Biomater. 106, 54–69. 10.1016/j.actbio.2020.02.003 32044456

[B236] Vogt L. Ruther F. Salehi S. Boccaccini A. R. (2021). Poly(Glycerol sebacate) in biomedical applications—a review of the recent literature. Adv. Healthc. Mater. 10 (9), 2002026. 10.1002/adhm.202002026 PMC11468981 33733604

[B237] Wang C. Xia K. Zhang Y. Kaplan D. L. (2019a). Silk-based advanced materials for soft electronics. Accounts Chem. Res. 52 (10), 2916–2927. 10.1021/acs.accounts.9b00333 31536330

[B238] Wang C. Yokota T. Someya T. (2021). Natural biopolymer-based biocompatible conductors for stretchable bioelectronics. Chem. Rev. 121 (4), 2109–2146. 10.1021/acs.chemrev.0c00897 33460327

[B239] Wang C. H. Dong Y. Q. Sengothi K. Tan K. L. Kang E. T. (1999). *In-vivo* tissue response to polyaniline. Synth. Met. 102 (1), 1313–1314. 10.1016/S0379-6779(98)01006-6

[B240] Wang K. Tian L. Wang T. Zhang Z. Gao X. Wu L. (2019b). Electrodeposition of alginate with PEDOT/PSS coated MWCNTs to make an interpenetrating conducting hydrogel for neural interface. Compos. Interfaces 26 (1), 27–40. 10.1080/09276440.2018.1465766

[B241] Wang L. Wu Y. Hu T. Guo B. Ma P. X. (2017a). Electrospun conductive nanofibrous scaffolds for engineering cardiac tissue and 3D bioactuators. Acta Biomater. 59, 68–81. 10.1016/j.actbio.2017.06.036 28663141

[B242] Wang M. Chen Y. Khan R. Liu H. Chen C. Chen T. (2019c). A fast self-healing and conductive nanocomposite hydrogel as soft strain sensor. Colloids Surfaces A Physicochem. Eng. Aspects 567, 139–149. 10.1016/j.colsurfa.2019.01.034

[B243] Wang Q. Ling S. Liang X. Wang H. Lu H. Zhang Y. (2019d). Self-healable multifunctional electronic tattoos based on silk and graphene. Adv. Funct. Mater. 29 (16), 1808695. 10.1002/adfm.201808695

[B244] Wang S. Sun C. Guan S. Li W. Xu J. Ge D. (2017b). Chitosan/gelatin porous scaffolds assembled with conductive poly(3,4-ethylenedioxythiophene) nanoparticles for neural tissue engineering. J. Mater. Chem. B 5 (24), 4774–4788. 10.1039/c7tb00608j 32264320

[B245] Wang Z. Wei H. Huang Y. Wei Y. Chen J. (2023). Naturally sourced hydrogels: emerging fundamental materials for next-generation healthcare sensing. Chem. Soc. Rev. 52 (9), 2992–3034. 10.1039/d2cs00813k 37017633

[B246] Wei L. Wang S. Shan M. Li Y. Wang Y. Wang F. (2023). Conductive fibers for biomedical applications. Bioact. Mater. 22, 343–364. 10.1016/j.bioactmat.2022.10.014 36311045 PMC9588989

[B247] Won S. M. Koo J. Crawford K. E. Mickle A. D. Xue Y. Min S. (2018). Natural wax for transient electronics. Adv. Funct. Mater. 28 (32), 1801819. 10.1002/adfm.201801819

[B248] Wu T. Cui C. Huang Y. Liu Y. Fan C. Han X. (2020). Coadministration of an adhesive conductive hydrogel patch and an injectable hydrogel to treat myocardial infarction. ACS Appl. Mater. Interfaces 12 (2), 2039–2048. 10.1021/acsami.9b17907 31859471

[B249] Xia T. Kovochich M. Liong M. Mädler L. Gilbert B. Shi H. (2008). Comparison of the mechanism of toxicity of zinc oxide and cerium oxide nanoparticles based on dissolution and oxidative stress properties. ACS Nano 2 (10), 2121–2134. 10.1021/nn800511k 19206459 PMC3959800

[B250] Xu J. Tsai Y.-L. Hsu S.-h. (2020). Design strategies of conductive hydrogel for biomedical applications. Molecules 25 (22), 5296. 10.3390/molecules25225296 33202861 PMC7698101

[B251] Xu K. Li S. Dong S. Zhang S. Pan G. Wang G. (2019a). Bioresorbable electrode array for electrophysiological and pressure signal recording in the brain. Adv. Healthc. Mater. 8 (15), 1801649. 10.1002/adhm.201801649 31168937

[B252] Xu X. Wang L. Jing J. Zhan J. Xu C. Xie W. (2022). Conductive collagen-based hydrogel combined with electrical stimulation to promote neural stem cell proliferation and differentiation. Front. Bioeng. Biotechnol. 10, 912497. 10.3389/fbioe.2022.912497 35782495 PMC9247657

[B253] Xu Y. Patino Gaillez M. Rothe R. Hauser S. Voigt D. Pietzsch J. (2021). Conductive hydrogels with dynamic reversible networks for biomedical applications. Adv. Healthc. Mater. 10 (11), 2100012. 10.1002/adhm.202100012 PMC11468162 33930246

[B254] Xu Y. Patsis P. A. Hauser S. Voigt D. Rothe R. Günther M. (2019b). Cytocompatible, injectable, and electroconductive soft adhesives with hybrid covalent/noncovalent dynamic network. Adv. Sci. 6 (15), 1802077. 10.1002/advs.201802077 PMC6685503 31406658

[B255] Xu Y. Yang X. Thomas A. K. Patsis P. A. Kurth T. Kräter M. (2018). Noncovalently assembled electroconductive hydrogel. ACS Appl. Mater. Interfaces 10 (17), 14418–14425. 10.1021/acsami.8b01029 29644843

[B256] Yadid M. Feiner R. Dvir T. (2019). Gold nanoparticle-integrated scaffolds for tissue engineering and regenerative medicine. Nano Lett. 19 (4), 2198–2206. 10.1021/acs.nanolett.9b00472 30884238

[B257] Yamaoka T. Tabata Y. Ikada Y. (1994). Distribution and tissue uptake of poly(ethylene glycol) with different molecular weights after intravenous administration to mice. J. Pharm. Sci. 83 (4), 601–606. 10.1002/jps.2600830432 8046623

[B258] Yang B. Yao F. Ye L. Hao T. Zhang Y. Zhang L. (2020). A conductive PEDOT/alginate porous scaffold as a platform to modulate the biological behaviors of brown adipose-derived stem cells. Biomaterials Sci. 8 (11), 3173–3185. 10.1039/c9bm02012h 32367084

[B259] Yang C. DelRio F. W. Ma H. Killaars A. R. Basta L. P. Kyburz K. A. (2016). Spatially patterned matrix elasticity directs stem cell fate. Proc. Natl. Acad. Sci. 113 (31), E4439–E4445. 10.1073/pnas.1609731113 27436901 PMC4978284

[B260] Yang G. Kampstra K. L. Abidian M. R. (2014). High performance conducting polymer nanofiber biosensors for detection of biomolecules. Adv. Mater. 26 (29), 4954–4960. 10.1002/adma.201400753 24719293 PMC4351750

[B261] Yang Q. Peng J. Xiao H. Xu X. Qian Z. (2022). Polysaccharide hydrogels: functionalization, construction and served as scaffold for tissue engineering. Carbohydr. Polym. 278, 118952. 10.1016/j.carbpol.2021.118952 34973769

[B262] Yao B. Wang H. Zhou Q. Wu M. Zhang M. Li C. (2017). Ultrahigh-conductivity polymer hydrogels with arbitrary structures. Adv. Mater. 29 (28), 1700974. 10.1002/adma.201700974 28513994

[B263] Yao G. Kang L. Li C. Chen S. Wang Q. Yang J. (2021). A self-powered implantable and bioresorbable electrostimulation device for biofeedback bone fracture healing. Proc. Natl. Acad. Sci. 118 (28), e2100772118. 10.1073/pnas.2100772118 34260393 PMC8285966

[B264] Yu K. J. Kuzum D. Hwang S.-W. Kim B. H. Juul H. Kim N. H. (2016). Bioresorbable silicon electronics for transient spatiotemporal mapping of electrical activity from the cerebral cortex. Nat. Mater. 15 (7), 782–791. 10.1038/nmat4624 27088236 PMC4919903

[B265] Yuk H. Wu J. Zhao X. (2022). Hydrogel interfaces for merging humans and machines. Nat. Rev. Mater. 7 (12), 935–952. 10.1038/s41578-022-00483-4

[B266] Zelikin A. N. Lynn D. M. Farhadi J. Martin I. Shastri V. Langer R. (2002). Erodible conducting polymers for potential biomedical applications. Angew. Chem. Int. Ed. 41 (1), 141–144. 10.1002/1521-3773(20020104)41:1<141::aid-anie141>3.0.co;2-v 12491465

[B267] Zhang S. Dong J. Pan R. Xu Z. Li M. Zang R. (2023). Structures, properties, and bioengineering applications of alginates and hyaluronic acid. Polymers 15 (9), 2149. 10.3390/polym15092149 37177293 PMC10181120

[B268] Zhang W. Wang R. Sun Z. Zhu X. Zhao Q. Zhang T. (2020). Catechol-functionalized hydrogels: biomimetic design, adhesion mechanism, and biomedical applications. Chem. Soc. Rev. 49 (2), 433–464. 10.1039/c9cs00285e 31939475 PMC7208057

[B269] Zhang Y. Chen S. Xiao Z. Liu X. Wu C. Wu K. (2021). Magnetoelectric nanoparticles incorporated biomimetic matrix for wireless electrical stimulation and nerve regeneration. Adv. Healthc. Mater. 10 (16), 2100695. 10.1002/adhm.202100695 34176235

[B270] Zhang Y. Zhou J. Zhang Y. Zhang D. Yong K. T. Xiong J. (2022). Elastic fibers/fabrics for wearables and bioelectronics. Adv. Sci. 9 (35), 2203808. 10.1002/advs.202203808 PMC9762321 36253094

[B271] Zhang Y. Zhou M. Dou C. Ma G. Wang Y. Feng N. (2019). Synthesis and biocompatibility assessment of polyaniline nanomaterials. J. Bioact. Compatible Polym. 34 (1), 16–24. 10.1177/0883911518809110

[B272] Zhao G. Feng Y. Xue L. Cui M. Zhang Q. Xu F. (2022). Anisotropic conductive reduced graphene oxide/silk matrices promote post-infarction myocardial function by restoring electrical integrity. Acta Biomater. 139, 190–203. 10.1016/j.actbio.2021.03.073 33836222

[B273] Zhao G. Zhou H. Jin G. Jin B. Geng S. Luo Z. (2022b). Rational design of electrically conductive biomaterials toward excitable tissues regeneration. Prog. Polym. Sci. 131, 101573. 10.1016/j.progpolymsci.2022.101573

[B274] Zhu R. Sun Z. Li C. Ramakrishna S. Chiu K. He L. (2019). Electrical stimulation affects neural stem cell fate and function *in vitro* . Exp. Neurol. 319, 112963. 10.1016/j.expneurol.2019.112963 31125549

[B275] Zhu T. Ni Y. Biesold G. M. Cheng Y. Ge M. Li H. (2023a). Recent advances in conductive hydrogels: classifications, properties, and applications. Chem. Soc. Rev. 52 (2), 473–509. 10.1039/d2cs00173j 36484322

[B276] Zhu W. Zhang J. Wei Z. Zhang B. Weng X. (2023b). Advances and progress in self-healing hydrogel and its application in regenerative medicine. Materials 16 (3), 1215. 10.3390/ma16031215 36770226 PMC9920416

[B277] Zou Y. Qin J. Huang Z. Yin G. Pu X. He D. (2016). Fabrication of aligned conducting PPy-PLLA fiber films and their electrically controlled guidance and orientation for neurites. ACS Appl. Mater. Interfaces 8 (20), 12576–12582. 10.1021/acsami.6b00957 27172537

